# Integrated Experimental and Computational Investigation of Fluorinated Benzoylthiourea Derivatives: Structural Characterization, Hirshfeld Surface Analysis, Antioxidant Activity, and MAO–B Inhibitory Potential for Parkinson’s Disease

**DOI:** 10.1021/acsomega.6c07002

**Published:** 2026-09-28

**Authors:** Lana Gul, Ummuhan Solmaz, Hakan Arslan

**Affiliations:** † 52983 Mersin University, Faculty of Science, Department of Chemistry, Mersin 33343, Türkiye; ‡ 52983 Mersin University, Technical Science Vocational School, Department of Chemistry and Chemical Processing Technologies, Mersin 33343, Türkiye

## Abstract

Three novel fluorinated benzoylthiourea derivatives, namely FBTU1–FBTU3, were successfully synthesized in 79–86% yields and fully characterized using spectroscopic techniques including FT–IR, ^1^H, ^13^C and ^19^F NMR, UV–Vis spectroscopy, elemental analysis, and single–crystal X–ray diffraction (SCXRD). All three compounds crystallize in the monoclinic system (*P*2_1_/*c* for FBTU1 and FBTU3; *P*2_1_/*n* for FBTU2) with *Z* = 4. The crystallographic analysis revealed well–defined molecular geometries stabilized by intramolecular and intermolecular interactions. Hirshfeld surface and energy framework analyses indicate that the crystal packing is primarily governed by centrosymmetric N–H···S hydrogen bonds (D···A = 3.432–3.483 Å), together with supporting π···π stacking and C–H···π interactions. The calculated total lattice energies were −211.9, −220.6, and −216.7 kJ/mol for FBTU1–FBTU3, respectively. The density functional theory (DFT) calculations were performed, and the results showed good agreement with the experimental structural parameters. Frontier molecular orbital analysis indicated significant electronic delocalization across the benzoylthiourea framework, influenced by the position of the fluorine substituent. The compounds showed moderate DPPH radical-scavenging activity. In addition, ADMET prediction studies indicated favorable pharmacokinetic profiles, including acceptable absorption characteristics and overall drug–likeness behavior, suggesting their potential suitability for further biological evaluation. Molecular docking studies against monoamine oxidase–B (MAO–B, PDB ID: 1GOS) identified FBTU1 as the compound with the most favorable calculated binding energy (Δ*G* = −8.12 kcal/mol, *K*_i_ = 1.13 μM).

## Graphical abstract

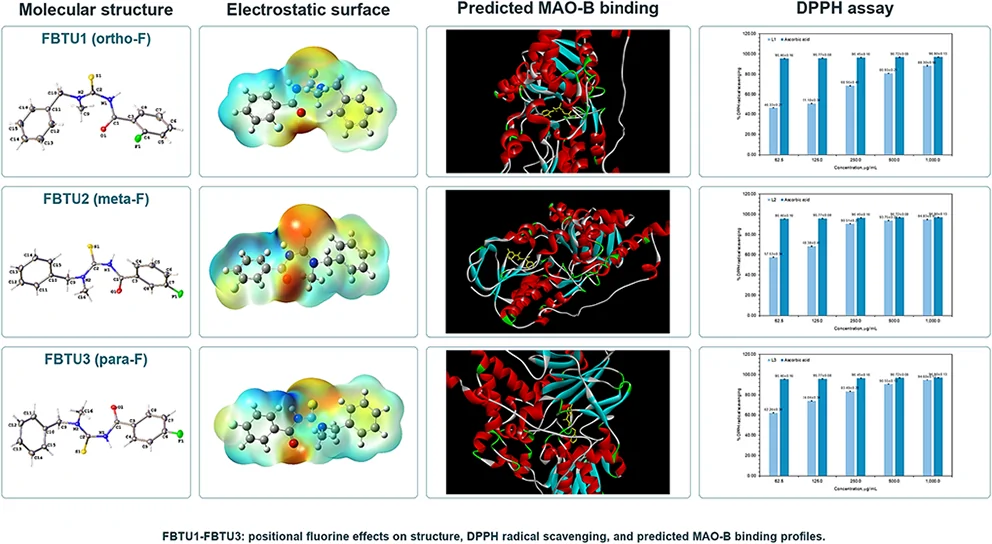

## 1 Introduction

Parkinson’s disease (PD) is a progressive neurodegenerative disorder characterized by the gradual loss of dopamine–producing neurons in the central nervous system.^1^ It manifests with clinical symptoms such as impaired motor control, tremor, muscle rigidity, and facial dyskinesia.^2^ Affecting millions of people worldwide, PD is predominantly observed in elderly individuals and significantly reduces quality of life. Current treatment strategies, primarily based on dopamine replacement, can only temporarily alleviate symptoms and remain insufficient in halting disease progression or reversing neurodegeneration.^3^ This limitation makes the discovery of new and effective therapeutic molecules for PD a critical area of research.^4–8^

Benzoylthiourea derivatives have recently attracted considerable attention in the fields of organic chemistry and biochemistry.^9–13^ Due to the presence of carbonyl (C=O) and thiourea (C=S) functional groups, these compounds exhibit high reactivity and can participate in various chemical reactions.^14^ Furthermore, their sulfur– and nitrogen–containing structures enable them to form complexes with metal ions, making them applicable in catalytic processes, selective sensing systems, and biological applications.^10,15–17^ Benzoylthiourea derivatives are considered biologically active small molecules and are notable for their neuroprotective, antioxidant, and enzyme inhibitory properties.^15,18–20^ These characteristics position them as valuable candidates in both fundamental research and drug development studies.^19,21,22^ Although previous studies have demonstrated various biological activities of benzoylthiourea derivatives,^23^ a comprehensive evaluation in the context of Parkinson’s disease has not yet been conducted. The relationship between their structural features and biological activities remains insufficiently understood, particularly regarding the role of structural modifications on their biological effects. This gap limits the exploration of their potential applications in the treatment of neurodegenerative diseases.

One of the key therapeutic strategies in Parkinson’s disease involves the inhibition of monoamine oxidase B (MAO–B), an enzyme involved in dopamine metabolism.^24^ Although currently available MAO–B inhibitors demonstrate a certain level of efficacy, their long–term use is associated with side effects and limited effectiveness, necessitating the development of safer alternatives.^25^ MAO–B-directed assessment of acyl­(benzoyl)-thiourea derivatives remains comparatively limited. In this context, although sulfonyl-thiourea derivatives, including benzo­[*d*]­thiazole-based sulfonyl thioureas, have recently been reported as MAO-A/MAO-B inhibitors,^26^ MAO-B-directed evaluation of acyl­(benzoyl)-thiourea derivatives such as the present fluorinated benzoylthioureas remains comparatively unexplored. Therefore, evaluating these compounds in relation to Parkinson’s disease would contribute to the scientific literature and may facilitate the identification of novel therapeutic candidates.^27^ In addition to MAO–B inhibition, oxidative stress is recognized as one of the major pathological factors contributing to the progression of Parkinson’s disease. MAO–B catalyzes the oxidative deamination of dopamine, generating hydrogen peroxide and other reactive oxygen species (ROS) as byproducts, which may promote oxidative damage and dopaminergic neuronal degeneration.^28,29^ Consequently, compounds combining experimentally observed radical-scavenging activity with predicted MAO-B binding may provide a rational basis for further neuroprotective evaluation.

Previous studies from our group have examined structurally related thiourea/selenourea systems using crystallographic, Hirshfeld surface, DFT, and bioactivity-oriented analyses. Joha et al.^18^ reported fluoro-substituted benzoylthioureas bearing an *N*-(4-methoxy-2-methylphenyl)­carbamothioyl substituent (T1-T3), while other companion studies focused on selenourea derivatives or a Ru­(II)-benzoylthiourea complex.^30,31^ Building upon these findings, the present study was designed to determine whether these fluorine-position effects are preserved or altered when the same fluorinated benzoylthiourea framework is combined with a different *N*-substituent. By retaining the complete ortho-, meta-, and para-fluorinated isomeric series while replacing the *N*-(4-methoxy-2-methylphenyl) group with an *N*-benzyl-*N*-methyl substituent, the influence of fluorine substitution could be systematically re-evaluated in a distinct molecular environment and further investigated in the context of Parkinson’s disease through antioxidant evaluation and predicted MAO-B binding. In this context, the present study extends this series to a distinct neutral fluorinated acyl­(benzoyl)-thiourea family, FBTU1-FBTU3, containing an *N*-benzyl-*N*-methylcarbamothioyl fragment. Earlier fluorinated acylthiourea isomeric series showed that positional fluorine substitution generally preserved the nearly planar acylthiourea core, the preferred antiperiplanar orientation of the C=O and C=S groups, and the recurrent N–H···S-linked centrosymmetric dimer motif, while altering aromatic-ring orientations and other conformational or vibrational features.^32,34^ In a broader fluorinated benzoylthiourea library, antimicrobial activity was also dependent on the positions of fluorine on the aroyl and aryl rings, although the preferred substitution pattern varied among the tested microorganisms and did not establish a universal ortho/meta/para activity order.^33^ The present FBTU1–FBTU3 series follows the same general structural pattern by retaining the common acylthiourea framework and dominant N–H···S association, while fluorine positional isomerism modulates secondary packing contacts, electronic descriptors, and radical-scavenging behavior, with the meta- and para-fluoro derivatives displaying higher DPPH activity than the ortho-fluoro analogue.^32–34^ In the earlier fluoro-substituted benzoylthiourea study, single-crystal X-ray characterization was reported for T1, whereas the present work provides SCXRD-based structural characterization for all three positional isomers FBTU1-FBTU3.^18^ Likewise, the Ru­(II)-benzoylthiourea study used glutathione reductase as the docking target, whereas the present metal-free FBTU series is evaluated with respect to MAO-B binding and BBB-oriented ADMET properties.^31^ The structures of the synthesized compounds, namely *N*–(benzyl­(methyl)­carbamothioyl)–2–fluorobenzamide (FBTU1), *N*–(benzyl­(methyl)­carbamothioyl)–3–fluorobenzamide (FBTU2), and *N*–(benzyl­(methyl)­carbamothioyl)–4–fluorobenzamide (FBTU3), were elucidated using elemental analysis, FT–IR spectroscopy, UV–vis, ^1^H and ^13^C NMR spectroscopy, and mass spectrometry. Furthermore, suitable single crystals of the compounds were analyzed by single–crystal X–ray diffraction techniques to determine their molecular and crystal structures. Based on the obtained experimental and theoretical data, the relationship between the structural properties and biological activities of these compounds was elucidated, and the aim was to identify structurally characterized scaffolds suitable for further experimental assessment in Parkinson’s disease-oriented studies.

## 2 Experimental Section

### 2.1 Materials

All reagents and solvents were commercially available and used as received without further purification. Potassium thiocyanate, fluorobenzoyl chlorides (2–, 3–, and 4–fluorobenzoyl chloride), and *N*–methylbenzylamine were employed as starting materials for the synthesis of the target compounds. Acetone, dichloromethane, ethanol, and hydrochloric acid of analytical grade were used throughout the experimental procedures. Reaction progress was monitored by thin–layer chromatography (TLC) on silica gel–coated aluminum sheets. The crude products were purified by recrystallization, while crystalline materials suitable for subsequent analyses were obtained by slow evaporation from appropriate solvent systems.

### 2.2 Instrumentation

Various spectroscopic and analytical techniques were employed to elucidate the structural properties of the synthesized compounds. ^1^H and ^13^C NMR spectra were recorded on a Bruker Avance III UltrashieldPlus spectrometer operating at 400 MHz. FT–IR spectra were obtained using a PerkinElmer Frontier FT–IR/FIR/NIR spectrometer equipped with an ATR accessory. Elemental analyses (C, H, and N) were performed on a Carlo Erba 1106 elemental analyzer. Electronic absorption spectra were recorded on a Shimadzu UV–3100 UV–Vis spectrophotometer. Melting points were determined using a Mettler Toledo MP90 melting point apparatus and are reported uncorrected. Single–crystal X–ray diffraction data were collected on a Bruker APEX–II diffractometer using suitable crystals obtained by slow evaporation of the corresponding solvent systems.

### 2.3 Synthesis of Thiourea Derivatives

Compounds FBTU1–FBTU3 were synthesized according to the classical acyl isothiocyanate method originally described by Douglass and Dains,^35^ which is widely employed for the preparation of acyl thiourea derivatives. In this procedure, the corresponding fluorobenzoyl isothiocyanate intermediate was generated in situ via the reaction of fluorobenzoyl chloride (*o*–, *m*–, or *p*–fluorobenzoyl chloride) with potassium thiocyanate, followed by nucleophilic addition of *N*–methylbenzylamine. Initially, potassium thiocyanate (0.972 g, 10.0 mmol) was dissolved in anhydrous acetone (50 mL), and the appropriate fluorobenzoyl chloride (1.586 g, 10.0 mmol) was added dropwise under continuous stirring. The reaction mixture was refluxed at 50 °C for approximately 1 h to afford the corresponding fluorobenzoyl isothiocyanate intermediate. After cooling to room temperature, *N*–methylbenzylamine (1.212 g, 10.0 mmol) was added dropwise, and the resulting mixture was stirred for an additional 12 h. Upon completion of the reaction, the mixture was poured into 0.1 M HCl solution (750 mL). The resulting precipitate was collected by filtration and washed with distilled water to remove residual impurities. The crude products were purified by recrystallization through slow evaporation from a dichloromethane/ethanol (2:1, v/v) solvent system. The general synthetic route employed for the preparation of compounds FBTU1–3 is illustrated in Scheme 1.

**Scheme 1:**
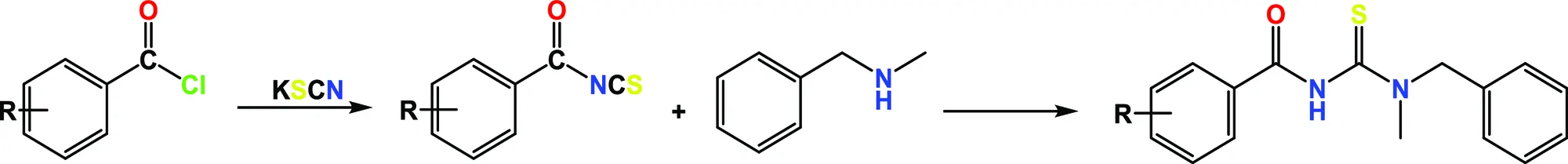
Synthesis of Compounds FBTU1–FBTU3 (R = *o*–, *m*–, and *p*–fluoro)

#### 2.3.1 *N*–(Benzyl­(methyl)­carbamothioyl)–2–fluorobenzamide (FBTU1)

Color: Yellow. Yield: 83%. Melting point: 153.5–154.9 °C. ^1^H NMR (400 MHz, DMSO–*d*_6_, δ, ppm): 11.10 (s, 1H, NH), 7.68 (t, *J* = 7.6 Hz, 1H, Ar–H), 7.61–7.57 (m, 1H, Ar–H), 7.40 (dd, *J* = 7.4, 2.8 Hz, 3H, Ar–H), 7.32 (dd, *J* = 12.0, 4.5 Hz, 4H, Ar–H), 5.27 (s, 2H, Ph–CH_2_–N), 3.15 (s, 3H, N–CH_3_). ^13^C NMR (100 MHz, DMSO–*d*_6_, δ, ppm): 181.78 (C=S), 162.42 (C=O), 159.88 (d, *J* = 250.3 Hz, C–F), 135.81, 133.98, 130.78, 129.15 (d, *J* = 23.6 Hz), 128.20, 127.87 (d, *J* = 10.8 Hz), 124.95, 123.55, 116.67 (d, *J* = 22.0 Hz), 58.12 (N–CH_2_), 41.86 (N–CH_3_). ^19^F NMR (376.5 MHz, DMSO–*d*_6_, δ, ppm): −113.48 (s, 1F, Ar–F). FT–IR (ATR, ν, cm^–1^): 3174 νNH, 3072, 3030, 3009 (νAr–H), 2985, 2920, 2896, 2862 (νAl–H), 1702 (νC=O), 1541 (C=C), 1208 (νC=S), 1056 (νC–F). UV–Vis (DCM, λ_max_, nm): 240.5, 287.5, 356.5.

#### 2.3.2 *N*–(Benzyl­(methyl)­carbamothioyl)–3–fluorobenzamide (FBTU2)

Color: White. Yield: 86%. Melting point: 134.5–135.5 °C. ^1^H NMR (400 MHz, DMSO–*d*_6_, δ, ppm): 11.01 (s, 1H, NH), 7.80 (dd, *J* = 16.8, 8.7 Hz, 2H, Ar–H), 7.56 (dd, *J* = 11.1, 3.6 Hz, 1H, Ar–H), 7.46–7.31 (m, 6H, Ar–H), 5.30 (s, 2H, Ph–CH_2_–N), 3.10 (s, 3H, N–CH_3_). ^13^C NMR (100 MHz, DMSO–*d*_6_, δ, ppm): 182.43 (C = S), 163.50 (C = O), 162.16 (d, *J* = 221.0 Hz, C–F), 135.77, 135.47, 131.11 (d, *J* = 8.1 Hz), 129.12 (d, *J* = 19.5 Hz), 128.17, 127.89 (d, *J* = 6.2 Hz), 125.09, 119.88 (d, *J* = 21.3 Hz), 115.71 (d, *J* = 23.1 Hz), 58.29 (N–CH_2_), 42.11 (N–CH_3_). ^19^F NMR (376.5 MHz, DMSO–*d*_6_, δ, ppm): −112.57 (s, 1F, Ar–F). FT–IR (ATR, ν, cm^–1^): 3177 (νNH), 3083, 3036, 3011 (νAr–H), 2975, 2935, 2913 (νAl–H), 1684 (νC=O), 1613 (νC=C), 1195 (νC=S), 1065 (νC–F). UV–Vis (DCM, λ_max_, nm): 238.5, 284.0, 350.5.

#### 2.3.3 *N*–(Benzyl­(methyl)­carbamothioyl)–4–fluorobenzamide (FBTU3)

Color: White. Yield: 79%. Melting point: 161.7–162.5 °C. ^1^H NMR (400 MHz, DMSO–*d*_6_, δ, ppm): 10.94 (s, 1H, NH), 8.06–8.03 (m, 2H, Ar–H), 7.46 (d, *J* = 7.4 Hz, 2H, Ar–H), 7.37 (dd, *J* = 16.8, 8.1 Hz, 5H, Ar–H), 5.31 (s, 2H, Ph–CH_2_–N), 3.09 (s, 3H, N–CH_3_). ^13^C NMR (100 MHz, DMSO–*d*_6_, δ, ppm): 182.70 (C=S), 165.08 (d, *J* = 250.2 Hz, C–F), 163.55 (C=O), 135.82, 131.80 (d, *J* = 9.4 Hz), 129.43 (d, *J* = 44.5 Hz), 129.01, 128.15, 127.87, 115.91 (d, *J* = 22.3 Hz), 58.29 (N–CH_2_), 42.08 (N–CH_3_). ^19^F NMR (376.5 MHz, DMSO–*d*_6_, δ, ppm): *-107.25* (s, 1F, Ar–F). FT–IR (ATR, ν, cm^–1^): 3188 (νNH), 3064 (νAr–CH), 2986, 2949, 2932, 2902 (νAl–CH), 1682 (νC=O), 1601 (νC=C), 1157 (νC=S), 1050 (νC–F). UV–Vis (DCM, λ_max_, nm): 235.5, 283.5, 348.5.

### 2.4 Crystal Structure Analysis

Suitable single crystals of FBTU1–FBTU3 were obtained by slow evaporation of their corresponding solutions at room temperature and were used for single–crystal X–ray diffraction studies. Single–crystal X–ray diffraction data were collected at 298.15 K using a Bruker APEX–II CCD diffractometer. Mo Kα radiation (λ = 0.71073 Å) was used for FBTU1 and FBTU2, whereas Cu Kα radiation (λ = 1.54178 Å) was employed for FBTU3. Data collection, integration, and absorption corrections were carried out using the Bruker APEX III software package.^36^ The crystal structures were solved by intrinsic phasing with SHELXT^37^ and refined by full–matrix least–squares refinement on F^2^ using SHELXL,^38^ as implemented in Olex2.^39^ All non–hydrogen atoms were refined anisotropically. Hydrogen atoms were placed in geometrically calculated positions and refined using a riding model with isotropic displacement parameters set to 1.2Ueq of the parent atoms. Molecular structures and graphical representations were generated using Olex2.^39^ Additional structural analyses, including intermolecular interactions and packing diagrams, were performed using Mercury software.^40^ Least–squares plane calculations and visualization of supramolecular features were carried out with standard crystallographic tools.^41^ Crystallographic data and structure refinement parameters for FBTU1–FBTU3 are summarized in Table 1.

**Table 1:** Crystal Data and Structure Refinement for FBTU1–FBTU3

Identification code	FBTU1	FBTU2	FBTU3
Empirical formula	C_16_H_15_FN_2_OS	C_16_H_15_FN_2_OS	C_16_H_15_FN_2_OS
Formula weight (g/mol)	302.36	302.36	302.36
Temperature (K)	298.15	298.15	298.15
Crystal system	Monoclinic	Monoclinic	Monoclinic
Space group	*P*2_1_/*c*	*P*2_1_/*n*	*P*2_1_/*c*
*a* (Å)	5.7768(3)	10.7482(6)	11.1451(16)
*b* (Å)	12.8401(7)	4.9333(3)	4.9011(7)
*c* (Å)	19.9756(12)	28.2811(17)	26.968(4)
α (°)	90	90	90
β (°)	94.128(2)	95.511(2)	91.829(7)
γ (°)	90	90	90
Volume (Å^3^)	1477.84(14)	1492.65(15)	1472.3(4)
*Z*	4	4	4
ρ_calc_ (g/cm^3^)	1.359	1.345	1.364
μ (mm^–1^)	0.230	0.227	2.056
F(000)	632.0	632.0	632.0
Crystal size (mm^3^)	0.323 × 0.166 × 0.143	0.216 × 0.063 × 0.03	0.187 × 0.116 × 0.084
Radiation	Mo Kα (λ = 0.71073)	Mo Kα (λ = 0.71073)	Cu Kα (λ = 1.54178)
2Θ range for data collection(°)	6.346 to 52.028	5.788 to 55.08	6.558 to 153.264
Index ranges	–7 ≤ *h* ≤ 6, –15 ≤ *k* ≤ 15, –24 ≤ *l* ≤ 24	–13 ≤ *h* ≤ 13, –6 ≤ *k* ≤ 6, –36 ≤ *l* ≤ 36	–13 ≤ *h* ≤ 13, –5 ≤ *k* ≤ 6, –33 ≤ *l* ≤ 33
Reflections collected	38,267	26,195	26,457
Independent reflections	2915 [*R*_int_ = 0.0708, *R*_sigma_ = 0.0267]	3429 [*R*_int_ = 0.0886, *R*_sigma_ = 0.0606]	2964 [*R*_int_ = 0.0713, *R*_sigma_ = 0.0510]
Data/restraints/parameters	2915/0/196	3429/0/195	2964/0/196
Goodness–of–fit on F^2^	1.046	1.035	1.087
Final *R* indexes [*I* ≥ 2σ (I)]	*R*_1_ = 0.0416, w*R*_2_ = 0.0889	*R*_1_ = 0.0597, w*R*_2_ = 0.1024	*R*_1_ = 0.0390, w*R*_2_ = 0.1010
Final R indexes [all data]	*R*_1_ = 0.0666, w*R*_2_ = 0.1010	*R*_1_ = 0.1241, w*R*_2_ = 0.1208	*R*_1_ = 0.0667, w*R*_2_ = 0.1092
Largest diff. peak/hole (e·Å^–3^)	0.18/–0.19	0.19/–0.29	0.18/–0.22

### 2.5 Hirshfeld Surface Analysis

Hirshfeld surface analyses of FBTU1–FBTU3 were performed using CrystalExplorer 17.5 software^42,43^ based on the corresponding single–crystal X–ray diffraction data. The crystallographic information files (CIFs) were imported into the program and used for the generation and visualization of molecular Hirshfeld surfaces, enabling a detailed investigation of intermolecular interactions within the crystal packing. Surface mappings, including normalized contact distance (*d*_norm_), shape index, and curvedness, were employed to identify and characterize specific intermolecular contacts. The associated fingerprint plots were generated to quantitatively analyze the contributions of intermolecular interactions through the internal (*d*_i_) and external (*d*_e_) contact distances. In addition, intermolecular interaction energies were calculated using the TONTO–based energy model integrated within CrystalExplorer, in combination with the Gaussian 16 package.^44,45^ All calculations were performed using the B3LYP functional with the 6–31G­(d,p) basis set. Energy framework diagrams were generated to visualize the topology and strength of intermolecular interactions in the crystal structures.^44^

### 2.6 DFT Calculations

Density functional theory (DFT) calculations for all synthesized compounds were carried out using the Gaussian 16 software package at the B3LYP/6–311G­(d,p) level of theory.^45^ The B3LYP functional combined with the 6–311G­(d,p) basis set was selected due to its well–established reliability and its balanced accuracy–computational cost performance in describing the structural and electronic properties of thiourea derivatives.^47^ In particular, the inclusion of polarization functions on both heavy atoms and hydrogen atoms at this level provides an appropriate description of the nonuniform electron distribution around the C=O, C=S, and N–H functionalities characteristic of the acylthiourea framework.^48,49^ All calculations were performed in the gas phase without inclusion of solvent effects in order to evaluate the intrinsic electronic properties of the molecules, consistent with standard comparative DFT methodologies.^50^ Geometry optimizations were followed by the extraction of key quantum chemical descriptors, including dipole moment, electronegativity, chemical potential, global hardness, softness, and HOMO–LUMO energy gap.^51^ Frontier molecular orbitals were visualized using GaussView 6 software.^52^ In addition, Mulliken population analysis was performed to investigate the distribution of atomic charges across the molecular framework of the studied compounds.

### 2.7 ADMET Analysis

The pharmacokinetic properties of the synthesized compounds were evaluated using in silico ADME (Absorption, Distribution, Metabolism, and Excretion) prediction tools, including SwissADME^53^ and pkCSM web platforms.^54^ These complementary computational approaches were employed to obtain a comprehensive assessment of the drug–likeness and pharmacokinetic behavior of the investigated molecules. A range of key physicochemical descriptors, including lipophilicity (logP), topological polar surface area (TPSA), molecular weight, hydrogen bond donor and acceptor counts, and rotatable bond numbers, were calculated to evaluate oral bioavailability and compliance with established drug–likeness rules. The obtained parameters were interpreted according to Lipinski,^55^ Veber,^56^ Egan,^57^ Ghose,^58^ and Muegge^59^ criteria. In addition, pkCSM was used to predict pharmacokinetic end points such as intestinal absorption, volume of distribution, blood–brain barrier permeability, and cytochrome P450 enzyme interaction profiles. A comprehensive toxicity profiling was performed using the ProTox–III web server,^60^ covering multiple toxicity end points, including hepatotoxicity, neurotoxicity, nephrotoxicity, respiratory toxicity, cardiotoxicity, carcinogenicity, immunotoxicity, mutagenicity, cytotoxicity, blood–brain barrier toxicity, ecotoxicity, clinical toxicity, and nutritional toxicity. These predictions were based on machine–learning–driven models and structural similarity–based toxicological inference. These integrated in silico analyses provided a preliminary evaluation of the pharmacokinetic behavior and safety profiles of the synthesized compounds prior to experimental biological studies.

### 2.8 Molecular Docking Studies

Molecular docking studies were performed to investigate the binding interactions and predicted binding profiles of the synthesized benzoylthiourea derivatives against monoamine oxidase–B (MAO–B, PDB ID: 1GOS), a key enzyme associated with Parkinson’s disease.^61–63^ PDB ID 1GOS was selected because it represents an experimentally determined human MAO–B structure with a structurally characterized inhibitor-binding region and well-defined entrance and substrate cavities, providing an appropriate receptor model for the exploratory evaluation of the predicted binding modes of FBTU1–FBTU3. The crystal structure of MAO–B was retrieved from the Protein Data Bank and prepared prior to docking by removing crystallographic water molecules and co–crystallized ligands, followed by the addition of hydrogen atoms and assignment of appropriate protonation states. Energy minimization was applied to obtain a stable receptor conformation suitable for docking simulations. Docking calculations were carried out using AutoDock 4.2.6 software^64^ employing the Lamarckian Genetic Algorithm (LGA). A total of 1000 docking runs were generated for each ligand, and the resulting conformations were clustered using an RMSD tolerance of 2.0 Å. The remaining LGA parameters were maintained at their default settings. The grid box was centered on the active binding pocket of MAO–B, defined based on catalytically important residues reported in the literature.^61,65–67^ The grid box dimensions were set to *x* × *y* × *z* = 60 × 60 × 60 Å^3^, with center coordinates defined as *x* = 56.967, *y* = 142.136, and *z* = 17.963, ensuring complete coverage of the active site region. For each ligand, multiple conformations were generated, and the best binding poses were selected based on binding free energy (Δ*G*) values and interaction profiles. Protein–ligand interactions, including hydrogen bonding, hydrophobic interactions, and π–π stacking, were analyzed using Discovery Studio Visualizer v25.1.^68^ The docking results were used to evaluate binding affinity and to provide insights into the structure–activity relationships of the synthesized compounds.

### 2.9 DPPH Free Radical Scavenging Assay

The DPPH radical scavenging assay was carried out based on previously reported methods with minor modifications.^30,69–71^ Stock solutions of the tested compounds were prepared in ethanol (1 mg/mL), and serial dilutions were obtained to yield final concentrations of 1000, 500, 250, 125, and 62.5 μg/mL. A DPPH solution (0.019 g in 100 mL ethanol) was freshly prepared and protected from light by wrapping in aluminum foil. Equal volumes of sample solutions and DPPH solution were mixed and incubated in the dark at room temperature for 1 h. A control sample containing only DPPH solution in ethanol was used as a reference. All measurements were performed in triplicate. After incubation, absorbance was measured at 517 nm using a UV–Vis spectrophotometer (Shimadzu UV–3100). Ascorbic acid was used as a positive control. Radical scavenging activity was calculated as percentage inhibition using eq 1:^72,73^

1 $$\%\mathrm{Inhibition}=\frac{A_{\mathrm{control}}-A_{\mathrm{sample}}}{A_{\mathrm{control}}}\times 100\%$$

where *A*_control_ represents the absorbance of the negative control and *A*_sample_ represents the absorbance of the test sample. In this way, the DPPH radical scavenging capacity of each compound was quantitatively determined.

## 3 Results and Discussion

### 3.1 Synthesis and Spectroscopic Characterization

In this study, three novel benzoylthiourea derivatives, namely *N*–(benzyl­(methyl)­carbamothioyl)–2–fluorobenzamide (FBTU1), *N*–(benzyl­(methyl)­carbamothioyl)–3–fluorobenzamide (FBTU2), and *N*–(benzyl­(methyl)­carbamothioyl)–4–fluorobenzamide (FBTU3), were successfully synthesized in moderate–to–good yields (79–86%) as yellow or white crystalline solids via condensation of the corresponding fluorobenzoyl isothiocyanates with *N*–benzylmethylamine in acetone. The fluorine substituent was positioned at the *ortho*–, *meta*–, and *para*–positions of the benzene ring, respectively. Structural characterization was carried out using proton and carbon nuclear magnetic resonance spectroscopy (^1^H NMR and ^13^C NMR), Fourier–transform infrared spectroscopy (FT–IR), ultraviolet–visible (UV–Vis) spectroscopy, and elemental analysis (Figures S1–S15). The obtained spectroscopic data showed excellent agreement with the proposed molecular structures of the synthesized compounds. Furthermore, single–crystal X–ray diffraction (SCXRD) analyses were performed for FBTU1–FBTU3, enabling detailed elucidation of their molecular conformations and crystal packing arrangements.

The ^1^H and ^13^C NMR spectra of the synthesized compounds were recorded in DMSO–*d*_6_ at frequencies of 400 and 100 MHz, respectively. The obtained chemical shift values (δ) were fully consistent with the proposed structures, and characteristic signals corresponding to the key proton and carbon environments were clearly identified in all derivatives. In the ^1^H NMR spectra, a singlet resonance observed at δ 10.94–11.10 ppm was assigned to the amide N–H proton. The downfield position of this signal is consistent with the participation of the amide proton in hydrogen–bonding interactions. Aromatic proton resonances appeared as multiplets within the range of δ 7.31–8.06 ppm. Minor variations in the distribution and chemical shifts of the aromatic proton signals were observed among the three derivatives, reflecting the influence of the fluorine substitution pattern on the electronic environment of the aromatic ring.^74,75^ The singlet signals observed at δ 5.27–5.31 ppm were attributed to the methylene (−C*H*_2_−) protons of the benzyl group, whereas the singlet resonances at δ 3.09–3.15 ppm corresponded to the N–methyl (−C*H*_3_) protons.^76,77^ The positions and multiplicities of these proton resonances strongly support the successful formation of the target benzyl­(methyl)­carbamothioyl fluorobenzamide derivatives.

The ^13^C NMR spectra further confirmed the proposed molecular structures. The thiocarbonyl (C=S) carbon resonances were observed at δ 181.78, 182.43, and 182.70 ppm for FBTU1, FBTU2, and FBTU3, respectively.^78^ These values are characteristic of thiocarbamoyl carbon atoms and are in good agreement with those reported for related thiourea derivatives.^76^ The carbonyl (C=O) carbon resonances appeared at δ 162.42, 163.50, and 163.55 ppm, confirming the presence of the amide functionality.^78^ Characteristic carbon–fluorine coupling was observed for the fluorine-bearing aromatic carbon at δ 159.88 (^1^ *J*C–F = 250.3 Hz), 162.16 (^1^ *J*C–F = 221.0 Hz), and 165.08 ppm (^1^ *J*C–F = 250.2 Hz) for FBTU1, FBTU2, and FBTU3, respectively. The remaining aromatic carbon resonances exhibited the characteristic C–F coupling pattern expected for fluorinated benzamide derivatives.^79,80^ For the para-substituted derivative (FBTU3), the fluorine-bearing aromatic carbon resonated at lower field than the carbonyl carbon, which can be attributed to the pronounced deshielding of the ipso aromatic carbon arising from direct C–F bonding in the para-fluorinated aromatic system.^80^ These observations indicate that the position of the fluorine substituent influences the electronic environment of the aromatic carbons and contributes to the differences observed in both the ^1^H and ^13^C NMR spectra. The methylene carbon of the benzyl group resonated at approximately δ 58 ppm, while the *N*–methyl carbon appeared near δ 42 ppm in all compounds. The ^13^C NMR data are fully consistent with the proposed structures and confirm the successful synthesis of the target fluorinated benzoylthiourea derivatives.

The fluorinated nature of the synthesized derivatives was further confirmed by ^19^F NMR spectroscopy (Figures S7–S9). Each compound exhibited a single sharp resonance at δ −113.48, −112.57, and −107.25 ppm for FBTU1, FBTU2, and FBTU3, respectively, consistent with the presence of one chemically distinct aromatic fluorine atom in each molecule. The relatively similar chemical shifts observed for FBTU1 and FBTU2 indicate comparable electronic environments around the fluorine nucleus, whereas the para-substituted derivative (FBTU3) exhibited a noticeable downfield shift, reflecting the influence of the substitution pattern on the electronic environment of the fluorine atom through inductive and resonance effects. Such substitution-dependent variations in ^19^F chemical shifts are well documented for fluorinated aromatic compounds.^81,82^ These results provide additional spectroscopic evidence supporting the successful synthesis and structural assignment of the three positional fluorinated benzoylthiourea isomers.

The FT–IR spectra of the synthesized compounds were recorded in ATR mode to verify the presence of the characteristic functional groups. In all compounds, N–H stretching vibrations were observed at 3174, 3177, and 3188 cm^–1^ for FBTU1, FBTU2, and FBTU3, respectively.^83–85^ These bands confirm the presence of the amide N–H functionality and are consistent with hydrogen–bonding interactions observed in related thiourea derivatives.^74^ Aromatic C–H stretching vibrations appeared in the region of 3083–3009 cm^–1^, while aliphatic C–H stretching vibrations were observed between 2986 and 2862 cm^–1^.^74^ The carbonyl stretching bands were detected at 1702, 1684, and 1682 cm^–1^ for FBTU1, FBTU2, and FBTU3, respectively, which fall within the characteristic range reported for amide carbonyl groups.^76^ Aromatic ring stretching vibrations appeared at 1541, 1613, and 1601 cm^–1^ for FBTU1, FBTU2, and FBTU3, respectively. The characteristic thiocarbonyl (C=S) stretching vibrations were observed in the range of 1157–1208 cm^–1^, confirming the presence of the thiocarbamoyl moiety.^74^ In addition, C–F stretching vibrations were detected at approximately 1050–1065 cm^–1^, supporting the incorporation of fluorine–substituted aromatic rings into the molecular framework. The data obtained from the FT–IR analysis indicate that all expected characteristic bands are present in each of the three compounds, confirming the successful formation of both carbonyl and thiocarbonyl functionalities within the synthesized structures. Minor variations in the vibrational frequencies were observed among the three derivatives depending on the position of the fluorine substituent (*ortho*, *meta*, and *para*). Nevertheless, the characteristic absorption bands associated with the principal functional groups remained essentially unchanged, indicating that fluorine substitution does not significantly alter the fundamental structural framework of the molecules.

To investigate the electronic transition behavior of the synthesized FBTU1, FBTU2, and FBTU3 compounds, UV–Vis spectra were recorded in dichloromethane as the solvent. The maximum absorption wavelengths (λ_max_) were observed at 240.5, 287.5, and 356.5 nm for FBTU1; 238.5, 284.0, and 350.5 nm for FBTU2; and 235.5, 283.5, and 348.5 nm for FBTU3. These absorption bands can be assigned to different electronic transitions associated with the molecular framework. The high–energy bands observed in the region of approximately 235–241 nm are attributed to π → π* transitions of the aromatic rings, consistent with the presence of conjugated benzene systems.^86,87^ The bands appearing in the range of 283–287 nm may be associated with *n* → π* and π → π* transitions involving the carbonyl (C=O) and thiocarbonyl (C=S) functionalities of the thiourea moiety.^74,88^ In addition, a weak and broad absorption band observed around 348–356 nm is tentatively assigned to intramolecular charge transfer (ICT) transitions within the conjugated system.^31,86^ This band may reflect electronic interactions between electron–rich and electron–deficient centers in the molecular structure, particularly involving the carbonyl and thiocarbonyl groups.^18^ Minor shifts in the absorption maxima among FBTU1–FBTU3 suggest that the position of the fluorine substituent (*ortho*, *meta*, and *para*) slightly influences the electronic distribution within the aromatic system. However, the overall spectral profiles remain similar, indicating that the fundamental π–conjugated framework is preserved across all derivatives. The UV–Vis results confirm that all compounds possess conjugated chromophoric systems, where electronic transitions are governed by both the aromatic rings and the thiourea–based functional groups. These features are consistent with structurally related thiourea derivatives reported in the literature.^74^

### 3.2 Crystal Structure Analysis

The molecular structures of FBTU1, FBTU2, and FBTU3 were determined by single–crystal X–ray diffraction analysis, and their ORTEP representations are presented in Figure 1a–c. All three compounds are isostructural in the sense that they share the same molecular formula (C_16_H_15_FN_2_OS, MW = 302.36 g/mol) and crystallize in the monoclinic crystal system with comparable unit cell volumes (∼1472–1493 Å^3^, *Z* = 4). FBTU1 and FBTU3 adopt the *P*2_1_/*c* space group, while FBTU2 crystallizes in *P*2_1_/*n*. The crystallographic analysis reveals that all three compounds exhibit well–defined molecular geometries consistent with the proposed acylthiourea framework. The bond lengths are summarized in Table S1. In all structures, the thiocarbonyl (C=S) bond distances were found to be 1.6744(19) Å (FBTU1), 1.678(2) Å (FBTU2), and 1.6820(17) Å (FBTU3), respectively. These values are consistent with typical C=S double–bond character observed in thiourea derivatives and indicate significant π–electron delocalization within the thiocarbonyl fragment.^89–91^ The carbonyl (C=O) bond lengths were determined as 1.212(2) Å for FBTU1, 1.217(3) Å for FBTU2, and 1.222(2) Å for FBTU3, confirming strong double–bond character of the amide functionality.^89,91^ The slightly increasing trend from FBTU1 to FBTU3 suggests minor electronic modulation induced by the fluorine substituent position.^89^ The N–C­(S) and N–C­(O) bond distances fall in the ranges of 1.321(2)–1.329(2) Å and 1.373(3)–1.384(2) Å, respectively. These shortened C–N bond lengths compared to standard single bonds indicate partial double–bond character arising from resonance delocalization over the O=C–N–C=S framework.^89^ This confirms the presence of extended conjugation within the acylthiourea backbone. In all three structures, aromatic C–C bond lengths within the benzene rings were observed in the range of approximately 1.355–1.397 Å, consistent with delocalized π–electron systems.^89^ The C–F bond distances were determined as 1.351(2) Å (FBTU1), 1.357(3) Å (FBTU2), and 1.355(3) Å (FBTU3), which are in good agreement with standard values reported for aryl fluorides 11,.^89,92,93^ The alkyl–related C–C bond lengths, particularly those associated with the methylene and aliphatic segments, were found in the range of 1.511–1.513 Å, consistent with normal single–bond character.^89^ In particular, the C10–C11/C15/C16 fragments confirm the integrity of the benzyl–linked substituent across all structures. The structural parameters obtained from SCXRD analysis clearly demonstrate that all three compounds share a common acylthiourea backbone with significant electron delocalization. The slight variations in bond lengths among FBTU1–FBTU3 indicate that the position of the fluorine substituent induces subtle electronic effects without disrupting the overall molecular framework.

**Figure 1:**
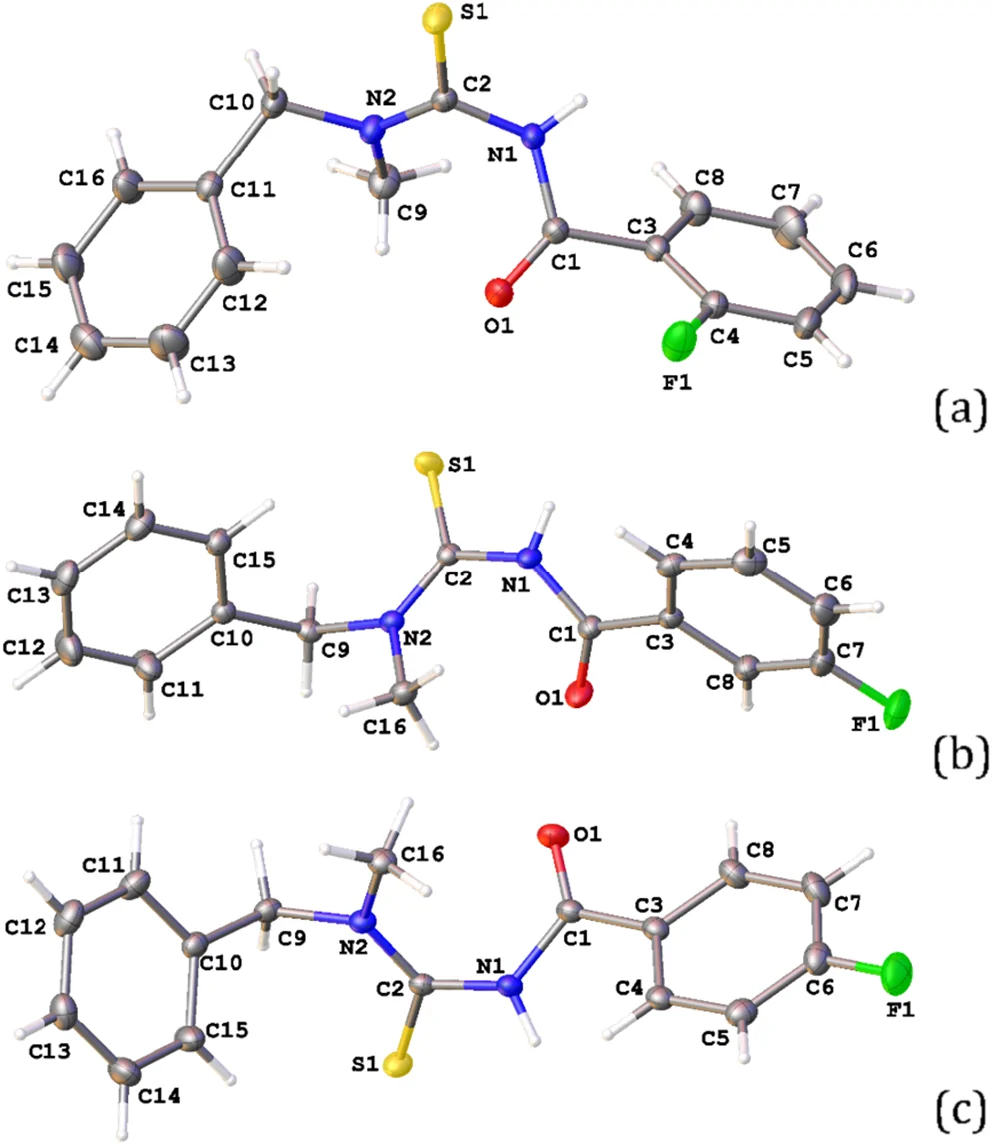
ORTEP representations of compounds (a) FBTU1, (b) FBTU2 and (c) FBTU3.

The bond angle values observed for FBTU1, FBTU2, and FBTU3 are consistent with the expected geometrical features of acyl thiourea derivatives and support the presence of a conjugated O=C–N–C=S framework (Table S2). In all three structures, the atoms surrounding the thiocarbonyl carbon (C2) exhibit a distorted trigonal planar geometry, indicating partial *sp* ^2^ character and delocalization within the thiourea backbone.^94^ For FBTU1, the N1–C2–S1 and N2–C2–S1 bond angles were determined as 118.49(14)° and 125.24(14)°, respectively, while the N1–C2–N2 angle was 116.19(17)°. In FBTU2, the corresponding angles were found to be 118.90(17)° (N1–C2–S1), 124.47(18)° (N2–C2–S1), and 116.6(2)° (N1–C2–N2). Similarly, for FBTU3, the N1–C2–S1, N2–C2–S1, and N1–C2–N2 angles were 118.32(13)°, 125.03(14)°, and 116.58(15)°, respectively. The observed widening of the N–C–S angles and corresponding compression of the N–C–N angles indicate significant electron delocalization within the thiourea moiety, consistent with resonance stabilization of the O=C–N–C=S fragment.^74,95^ The carbonyl carbon environments (C1) also display nearly ideal trigonal planar geometry.^96^ In FBTU1, the O1–C1–N1 and O1–C1–C3 angles were 123.63(17)° and 123.75(17)°, respectively, while the N1–C1–C3 angle was 112.61(16)°. Similar values were observed for FBTU2 [O1–C1–N1 = 122.5(2)°, O1–C1–C3 = 121.9(2)°, N1–C1–C3 = 115.6(2)°] and FBTU3 [O1–C1–N1 = 122.05(18)°, O1–C1–C3 = 123.02(17)°, N1–C1–C3 = 114.94(16)°]. These values confirm the *sp* ^2^ hybridization of the carbonyl carbon atoms and the delocalized nature of the amide functionality.^97^ Within the aromatic rings, the C–C–C bond angles generally range between 118–123°, consistent with planar hexagonal benzene geometry.^98^ Minor deviations from the ideal 120° angle are attributed to the influence of substituents, including fluorine atoms and amide linkages, which slightly perturb the electron distribution in the aromatic system. In FBTU1, for example, the F1–C4–C3 and F1–C4–C5 angles are 118.80(17)° and 117.75(19)°, respectively, indicating a small local distortion induced by the fluorine substituent. The N2–C9–C10 angle in all three structures (114.94° in FBTU1, 112.68° in FBTU2, and 111.11° in FBTU3) reflects slight geometric compression due to the presence of the amide–linked side chain, consistent with steric and electronic effects within the molecular framework.^90^ The bond angle parameters clearly demonstrate that all three compounds retain a highly conjugated acyl thiourea core with partial double–bond character distributed over the O=C–N–C=S fragment. The observed small variations among FBTU1–FBTU3 indicate that the position of the fluorine substituent induces only minor geometric perturbations without significantly disrupting the overall molecular planarity and conjugation.

### 3.3 Supramolecular Structure

The supramolecular architectures of FBTU1–FBTU3 are governed by a synergistic combination of N–H···S, C–H···O, C–H···N, C–H···S, C–H···π and π···π interactions, which collectively direct the self–assembly of the molecules into distinct higher–order crystal packing motifs. Detailed analysis of Figures 2–4 together with the hydrogen–bond geometries summarized in Table 2 reveals that subtle variations in secondary intermolecular interactions significantly influence the resulting supramolecular topology.^99,100^ The supramolecular features observed for FBTU1–FBTU3 are in accordance with the established crystal engineering principles of thioamide and acylthiourea systems, where intermolecular N–H···S hydrogen bonds constitute one of the predominant structure-directing synthons.^101–103^ Unlike sulfur-centered halogen-bonded assemblies reported for appropriate halogen-bond donor systems, the S···F separations in FBTU1–FBTU3 are beyond the range generally associated with significant C=S···halogen interactions.^104–106^ Consequently, the influence of fluorine substitution in the present series is manifested through modulation of weak intermolecular contacts rather than the formation of structure-directing sulfur-centered halogen bonds.

**Figure 2:**
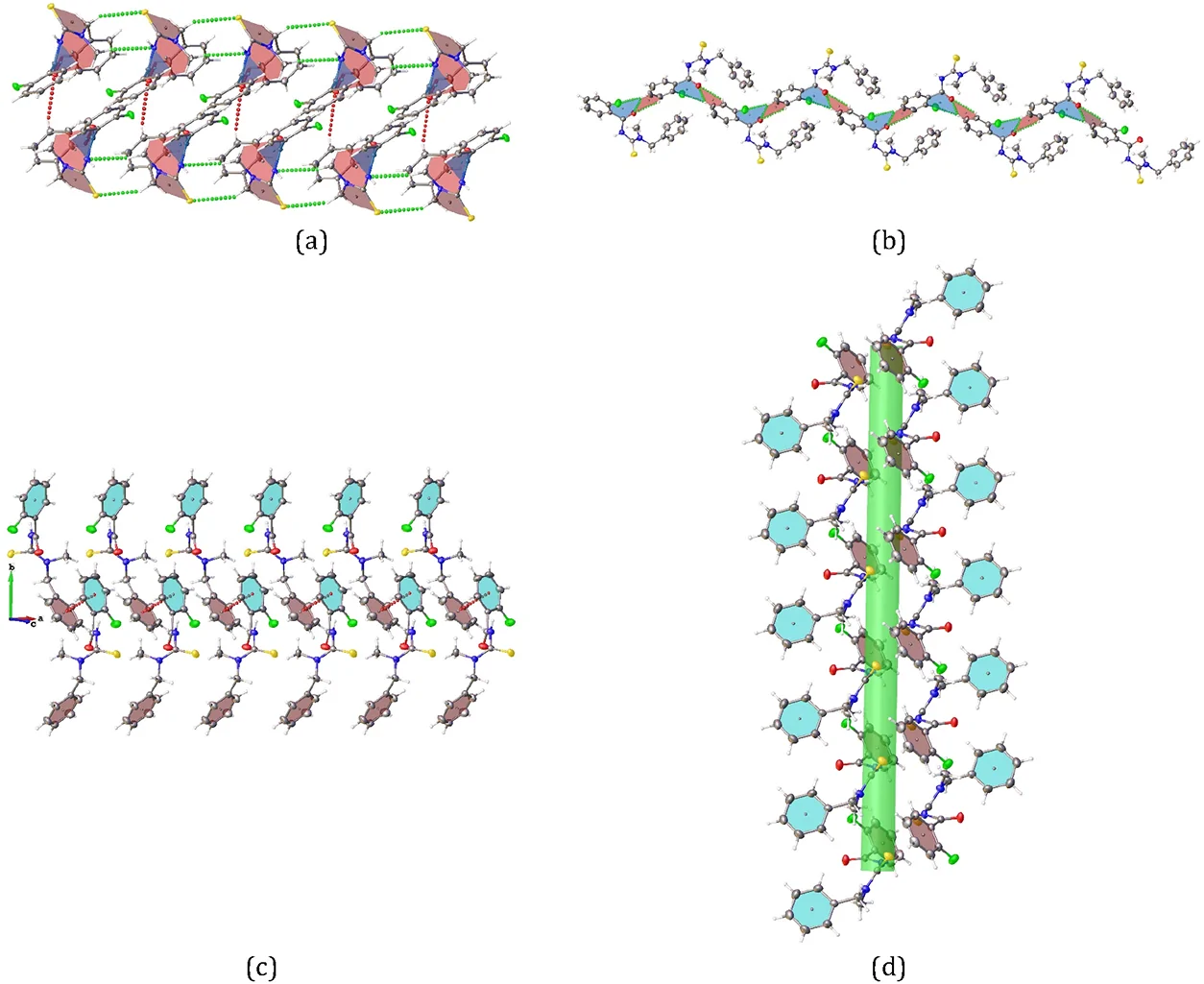
Crystal packing features of FBTU1 showing (a) *R*_2_^2^(8) supramolecular synthons, (b) fused intramolecular ring motifs, (c) aromatic π···π stacking interactions, and (d) the resulting helically organized supramolecular assembly.

**Table 2:** Hydrogen–Bond Geometry of Compounds FBTU1–FBTU3

No	D–H···A	Symmetry code	D–H (Å)	H···A (Å)	D···A (Å)	∠D–H···A (°)
FBTU1						
1	N1–H1···S1	2 – *x*, 1 – *y*, 1 – *z*	0.86(2)	2.63(2)	3.4320(18)	156.5(19)
2	C6–H6···F1	2 – *x*, 1/2 + *y*, 1/2 – *z*	0.93	2.45	3.363(3)	166
3	C9–H9C···O1	–	0.96	2.47	2.957(3)	111
4	C10–H10B···S1	–	0.97	2.57	3.065(2)	112
FBTU2						
1	N1–H1···S1	2 – *x*, −*y*, 1 – *z*	0.82(2)	2.64(2)	3.425(2)	161.3(18)
2	C9–H9B···S1	–	0.97	2.56	3.057(2)	112
3	C16–H16A···O1	–	0.96	2.43	2.870(3)	108
4	C16–H16C···N1	–	0.96	2.44	2.780(3)	100
FBTU3						
1	N1–H1···S1	2 – *x*, 2 – *y*, 1 – *z*	0.87(2)	2.64(2)	3.4825(17)	166(2)
2	C9–H9A···S1	–	0.97	2.60	3.0897(19)	112
3	C9–H9B···O1	1 – *x*, 2 – *y*, 1 – *z*	0.97	2.50	3.412(2)	156
4	C16–H16A···O1	–	0.96	2.45	2.898(3)	108
5	C16–H16B···N1	–	0.96	2.43	2.772(3)	100

For FBTU1, the crystal packing is primarily controlled by hydrogen–bonded synthons and aromatic stacking interactions (Figure 2). As illustrated in Figure 2a, intermolecular C9–H9B···N1 (3.179 Å) and C10–H10A···S1 (3.170 Å) interactions are arranged in a parallel fashion and generate centrosymmetric *R*_2_^2^(8) synthons. These motifs constitute the primary supramolecular building blocks within the lattice and organize neighboring molecules into an ordered hydrogen–bonding framework. Furthermore, intermolecular C14–H14···O1 interactions (3.037 Å) propagate approximately perpendicular to the *R*_2_^2^ (8) motif, resulting in an orthogonally interconnected network that further reinforces crystal stability. The importance of sulfur–centered interactions is supported by the crystallographic N1–H1···S1 hydrogen bond listed in Table 2, which exhibits an H···S distance of 2.63(2) Å, a D···A separation of 3.4320(18) Å, and a relatively linear N–H···S angle of 156.5(19)°, indicating a significant structure–directing contribution. The molecular conformation of FBTU1 is further stabilized through intramolecular contacts (Figure 2b). The C8–H8···N1 (2.750 Å) and C10–H10···S1 (2.570 Å) interactions generate fused five–membered pseudo–rings, whereas the C9–H9C···O1 interaction (2.469 Å) forms a seven–membered fused motif. These fused ring systems rigidify the molecular backbone and propagate throughout the crystal lattice, producing a characteristic wave–like supramolecular arrangement.^100,107^ Similar conformational locking effects have frequently been reported for benzoylthiourea derivatives, where intramolecular hydrogen bonds preorganize molecular geometry and facilitate crystal growth.^31,90,108,109^ As shown in Figure 2c, additional stabilization arises from π···π interactions between adjacent aromatic rings with a centroid–to–centroid distance of 4.221 Å, leading to a *face–*to*–face* stacking arrangement. Although weaker than classical hydrogen bonds, these interactions contribute substantially to packing efficiency through cumulative dispersion effects. The combined influence of *R*_2_^2^ (8) synthons, fused intramolecular ring motifs, and aromatic stacking interactions ultimately results in the formation of a helical supramolecular organization (Figure 2d). This helically propagated architecture represents one of the most distinctive features of FBTU1 and demonstrates how weak noncovalent interactions collectively direct the formation of higher–order crystal packing topologies.^108^

The supramolecular assembly of FBTU2 exhibits a markedly different organization pattern dominated by C–H···π, C–H···O, and sulfur–centered interactions (Figure 3). In Figure 3a, a C5–H5···Cg(1) interaction with an H···Cg distance of 3.532 Å establishes a significant C–H···π contact between neighboring aromatic fragments. Such interactions are widely recognized as auxiliary structure–directing forces that contribute to crystal stabilization and molecular association in aromatic thiourea systems.^108,110^ A more prominent feature of the FBTU2 crystal structure is observed in Figure 3b. The intramolecular C16–H16C···O1 (2.854 Å) and intermolecular C16–H16A···O1 (2.625 Å) interactions cooperate to generate consecutive *R*_2_^4^(6) synthons. These motifs are arranged periodically throughout the crystal lattice, giving rise to characteristic quadrangular supramolecular units. The corresponding intramolecular C16–H16A···O1 interaction identified crystallographically in Table 2 exhibits an H···O separation of 2.43 Å and D···A distance of 2.870(3) Å, confirming the importance of oxygen–centered interactions in conformational stabilization. Additional supramolecular organization arises from sulfur–centered interactions (Figure 3c). Intramolecular C15–H15···S1 (3.241 Å) and N1–H1···S1 (2.641 Å) contacts, together with intermolecular C15–H15···S1 (3.179 Å) and N1–H1···S1 (3.919 Å) interactions, generate centrosymmetric *R*_4_^4^ (4) synthons that assemble into quadrangular arrangements throughout the lattice. The significance of sulfur as a supramolecular acceptor is further supported by the crystallographic N1–H1···S1 interaction listed in Table 2 (H···S = 2.64(2) Å, D···A = 3.425(2) Å, ∠N–H···S = 161.3(18)°). Consequently, the combined influence of C–H···π interactions and oxygen– and sulfur–centered synthons produces a densely interconnected supramolecular framework characterized by repetitive quadrangular motifs.^111^

**Figure 3:**
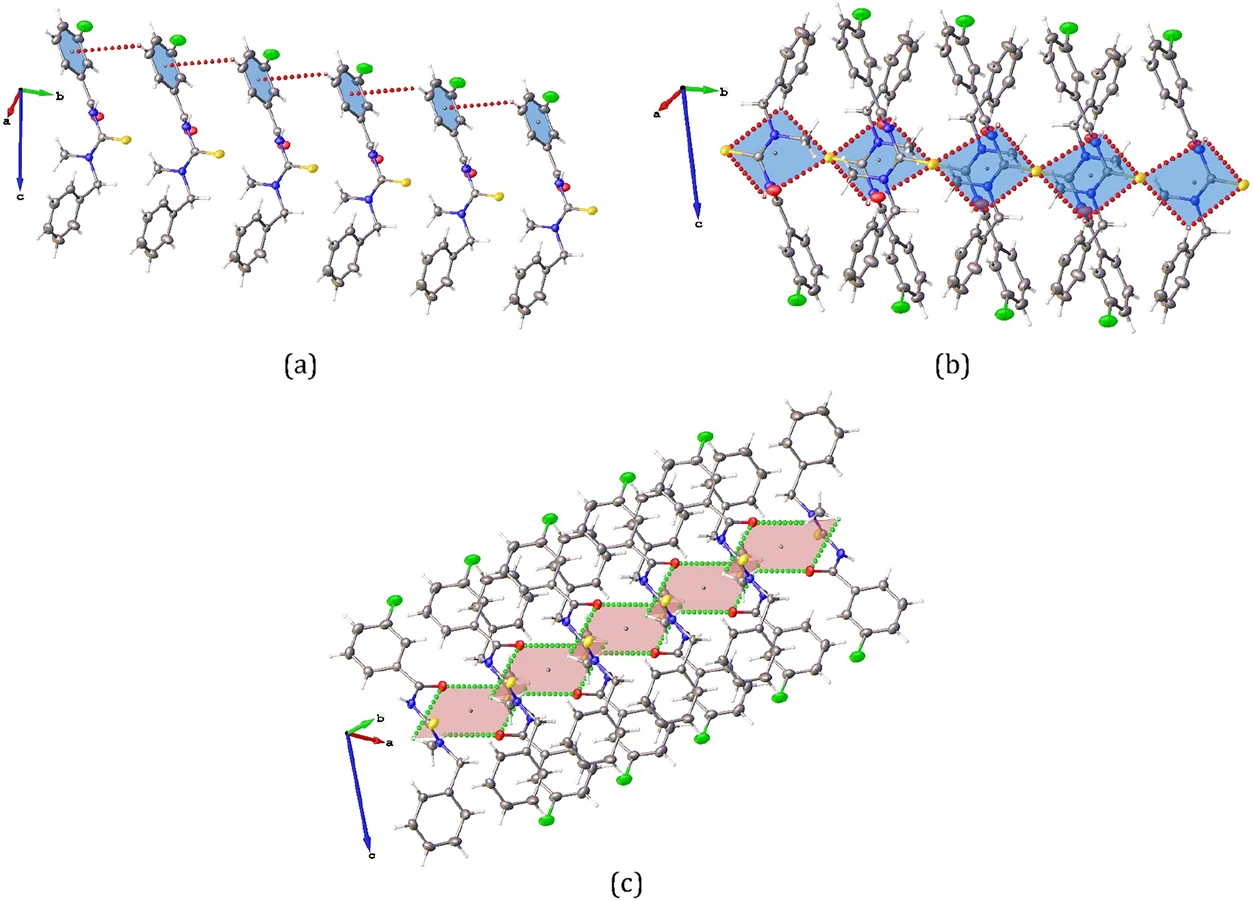
Supramolecular organization of FBTU2: (a) C–H···π interactions contributing to crystal stabilization, (b) formation of *R*_4_^4^(6) sulfur–centered synthons generating quadrangular supramolecular units, and (c) sequential *R*_2_^4^ (6)­oxygen–centered synthons producing an extended hydrogen–bonded framework.

Among the investigated compounds, FBTU3 displays the most elaborate supramolecular topology (Figure 4). As illustrated in Figure 4a, intermolecular N1–H1···S1 interactions (2.635 Å) generate centrosymmetric *R*_2_^2^(8) synthons resembling hexagonal supramolecular units. The corresponding crystallographic geometry (H···S = 2.64(2) Å, D···A = 3.4825(17) Å, ∠N–H···S = 166(2)°) indicates a highly directional hydrogen bond that effectively propagates the crystal packing. The most striking structural feature of FBTU3 is observed in Figure 4b. Intramolecular C8–H8···O1 (2.603 Å), C4–H4···N1 (2.561 Å), C9–H9A···S1 (2.598 Å), and C16–H16B···N1 (2.432 Å) interactions cooperatively generate fused five–membered pseudo–rings that rigidify the molecular framework. The periodic repetition of these fused motifs throughout the crystal lattice results in the formation of a well–defined helical chain.^112^ Such helically propagated architectures are relatively uncommon among fluorinated benzoylthiourea derivatives and emphasize the crucial role of conformational preorganization in directing crystal growth and supramolecular self–assembly.^18^ Further stabilization is provided by oxygen–centered interactions shown in Figure 4c. The intermolecular C11–H11···O1 (3.122 Å) and C9–H9B···O1 (2.504 Å) contacts generate *R*_2_^1^ (6) synthons that function as secondary supramolecular connectors between neighboring helical chains. Table 2 additionally confirms the significance of the C9–H9B···O1 interaction, which exhibits an H···O distance of 2.50 Å, a D···A separation of 3.412(2) Å, and a C–H···O angle of 156°, indicative of an effective directional interaction. The coexistence of *R*_2_^2^ (8) and *R*_2_^1^ (6) motifs therefore produce a highly interconnected three–dimensional supramolecular network.^113^

**Figure 4:**
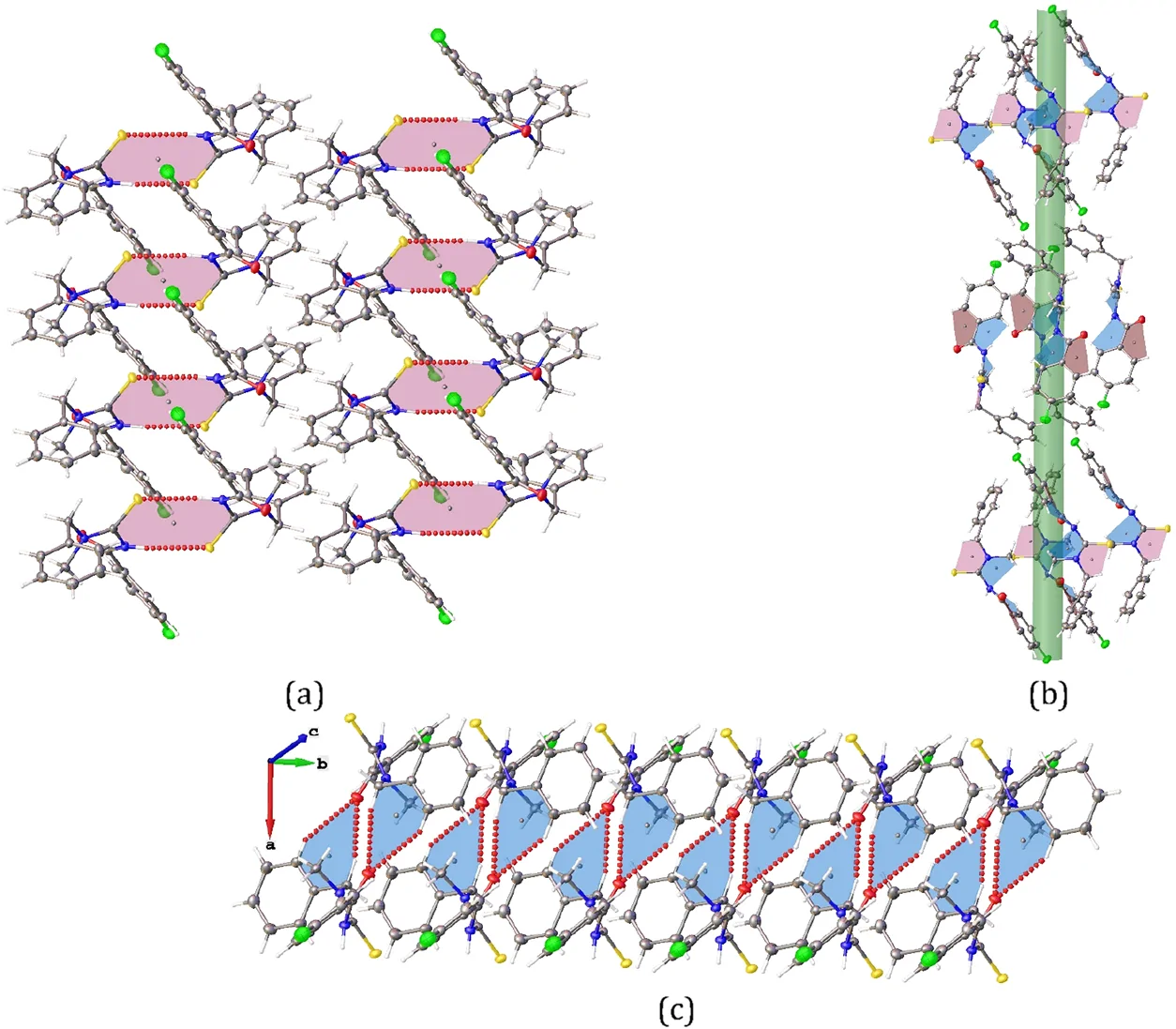
Crystal packing features of FBTU3 showing (a) *R*_2_^2^(6) supramolecular synthons, (b) helical chain assembly generated by fused intramolecular ring motifs, and (c) *R*_2_^1^ (6) hydrogen–bonded motifs reinforcing the supramolecular framework.

The solid–state organization of fluorinated benzoylthiourea derivatives is highly sensitive to subtle variations in intermolecular interactions, leading to the formation of distinct supramolecular architectures with different packing arrangements. Crystal engineering studies have demonstrated that structurally related molecules may adopt markedly different supramolecular topologies depending on the balance between hydrogen bonding, aromatic stacking, and other weak noncovalent interactions, which can ultimately influence their physicochemical and biological properties.^18^

In the present study, FBTU1–FBTU3 exhibit significantly different crystal packing motifs despite sharing the same benzoylthiourea backbone. The positional variation of the fluorine substituent also influences the relative orientation of neighboring molecules within the crystal lattice, as reflected in the distinct helical, quadrangular, and hydrogen-bonded packing arrangements observed for the three isomers. These observations highlight the remarkable structural diversity that can arise from subtle changes in molecular substitution patterns and demonstrate the ability of fluorinated benzoylthiourea derivatives to function as versatile supramolecular building blocks. Such differences in crystal packing are expected to influence intermolecular recognition processes, Hirshfeld surface contact distributions, and potentially the biological behavior of these compounds, including their antioxidant properties and MAO–B inhibitory activity relevant to Parkinson’s disease. Consistent with contemporary crystal–engineering concepts, the present results further demonstrate that small variations in noncovalent interaction patterns can direct the formation of substantially different supramolecular topologies and packing arrangements in the solid state.^114,115^

### 3.4 Hirshfeld Surface Analysis

Hirshfeld surface analysis, including *d*_norm_, *d*_i_, *d*_e_, shape index, curvedness, and fragment patch mapping, was performed using CrystalExplorer to provide a quantitative assessment of the intermolecular contacts governing the crystal packing of FBTU1–FBTU3 (Figure 5). In the *d*_norm_ maps, intense red spots are observed around the N–H donor and thiocarbonyl sulfur acceptor regions in all three compounds, confirming that N–H···S hydrogen bonds constitute the principal intermolecular interactions responsible for crystal stabilization. Additional localized red regions reflect C–H···F contacts in FBTU1 and C–H···O contacts in FBTU3, whereas the surface features of FBTU2 are predominantly associated with the sulfur-centered contacts.^46,96^ Although the overall distribution of interaction sites is similar for FBTU1–FBTU3, slight variations in the intensity and localization of the red patches suggest that the positional isomerism of the fluorine substituent influences the packing environment and the spatial arrangement of neighboring molecules. The *d*_i_ and *d*_e_ surfaces further highlight the thiocarbonyl sulfur, amide oxygen, amide nitrogen, and fluorine–substituted aromatic regions as the primary sites involved in short intermolecular contacts. The complementary features observed on the de surfaces indicate efficient donor–acceptor matching between adjacent molecules and are fully consistent with the hydrogen–bonding motifs identified by single–crystal X–ray diffraction analysis. The shape index maps display alternating red and blue surface features distributed over the aromatic rings, suggesting the presence of weak π–associated intermolecular contacts.^96^ Correspondingly, the curvedness surfaces exhibit extended flat green regions over the aromatic and benzoylthiourea fragments, which are characteristic of relatively planar molecular surfaces capable of supporting efficient crystal packing.^90,116^ Furthermore, the fragment patch representations clearly demonstrate that the thiocarbonyl sulfur atom, amide carbonyl group, fluorinated phenyl ring, and aromatic π–surfaces serve as the major interaction domains involved in constructing the three–dimensional supramolecular architectures of FBTU1–FBTU3. The Hirshfeld surface analysis confirms that strong N–H···S hydrogen bonds act as the primary packing force, while weaker C–H···O and π–related interactions provide additional stabilization to the crystal structures.^117^

**Figure 5:**
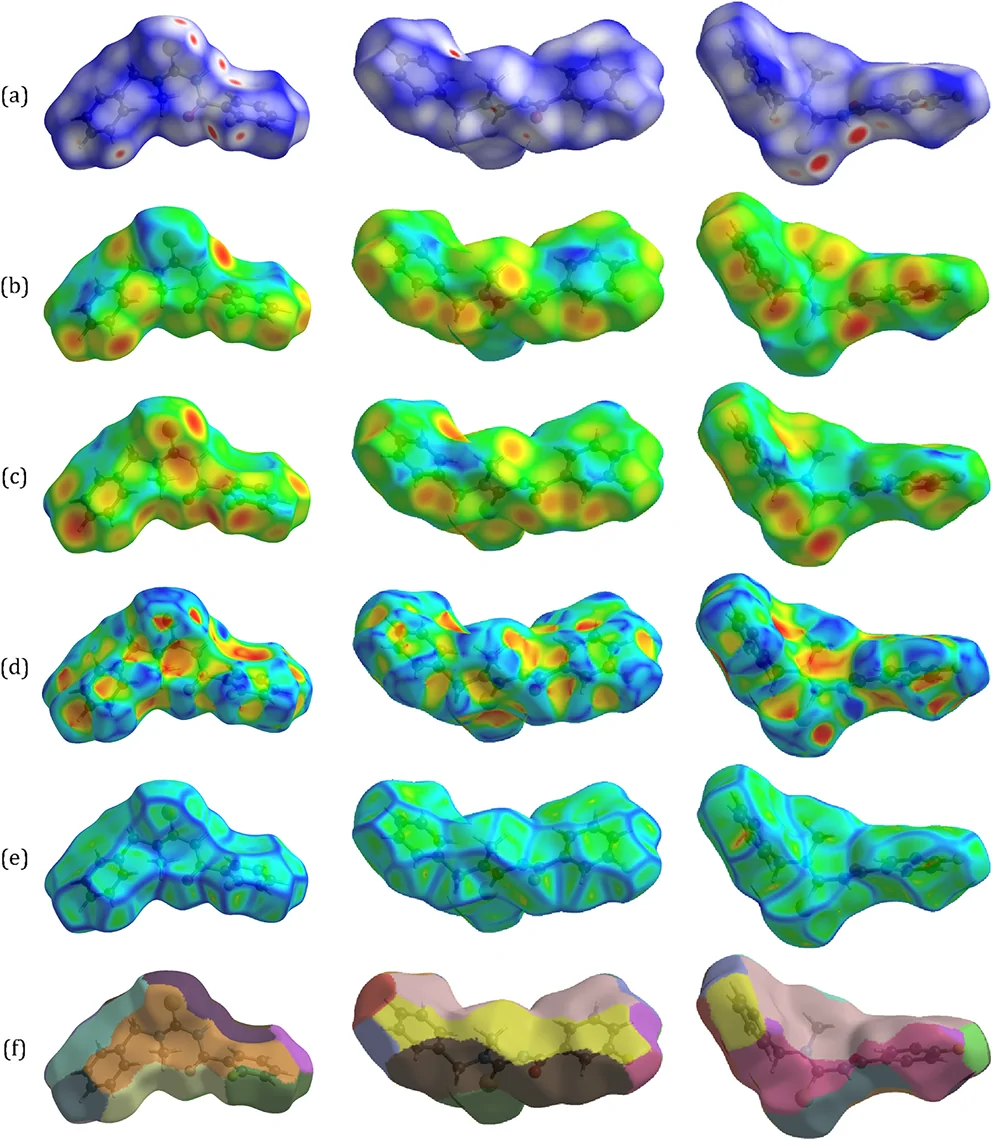
Hirshfeld surfaces of compounds FBTU1–FBTU3 mapped over (a) *d*_norm_, (b) *d*_i_, (c) *d*_e_, (d) shape index, (e) curvedness, and (f) fragment patch surfaces.

The two–dimensional fingerprint plots derived from the Hirshfeld surface analysis provide quantitative insight into the intermolecular interactions governing the crystal packing of FBTU1–FBTU3 (Figures S16–S18). In all three compounds, H···H contacts constitute the largest contribution to the Hirshfeld surfaces, accounting for 43.1% in FBTU1, 41.6% in FBTU2, and 41.1% in FBTU3 (Figure 6). The predominance of these contacts indicates that dispersion–driven van der Waals interactions make a substantial contribution to crystal stabilization and molecular packing efficiency, a feature commonly observed in molecular crystals dominated by weak intermolecular interactions. The second most significant contribution arises from C···H/H···C contacts, representing 17.5, 23.7, and 23.5% of the Hirshfeld surfaces of FBTU1, FBTU2, and FBTU3, respectively (Figure 6). These contacts generate the characteristic *wing*–like features in the fingerprint plots and are generally associated with C–H···π interactions involving neighboring aromatic rings. The noticeably higher contributions observed for FBTU2 and FBTU3 suggest that aromatic interactions become increasingly important upon relocation of the fluorine substituent from the ortho position to the meta and para positions. This observation is fully consistent with the crystal structure analyses, which revealed the participation of aromatic fragments in the formation of supramolecular motifs and extended packing arrangements.

**Figure 6:**
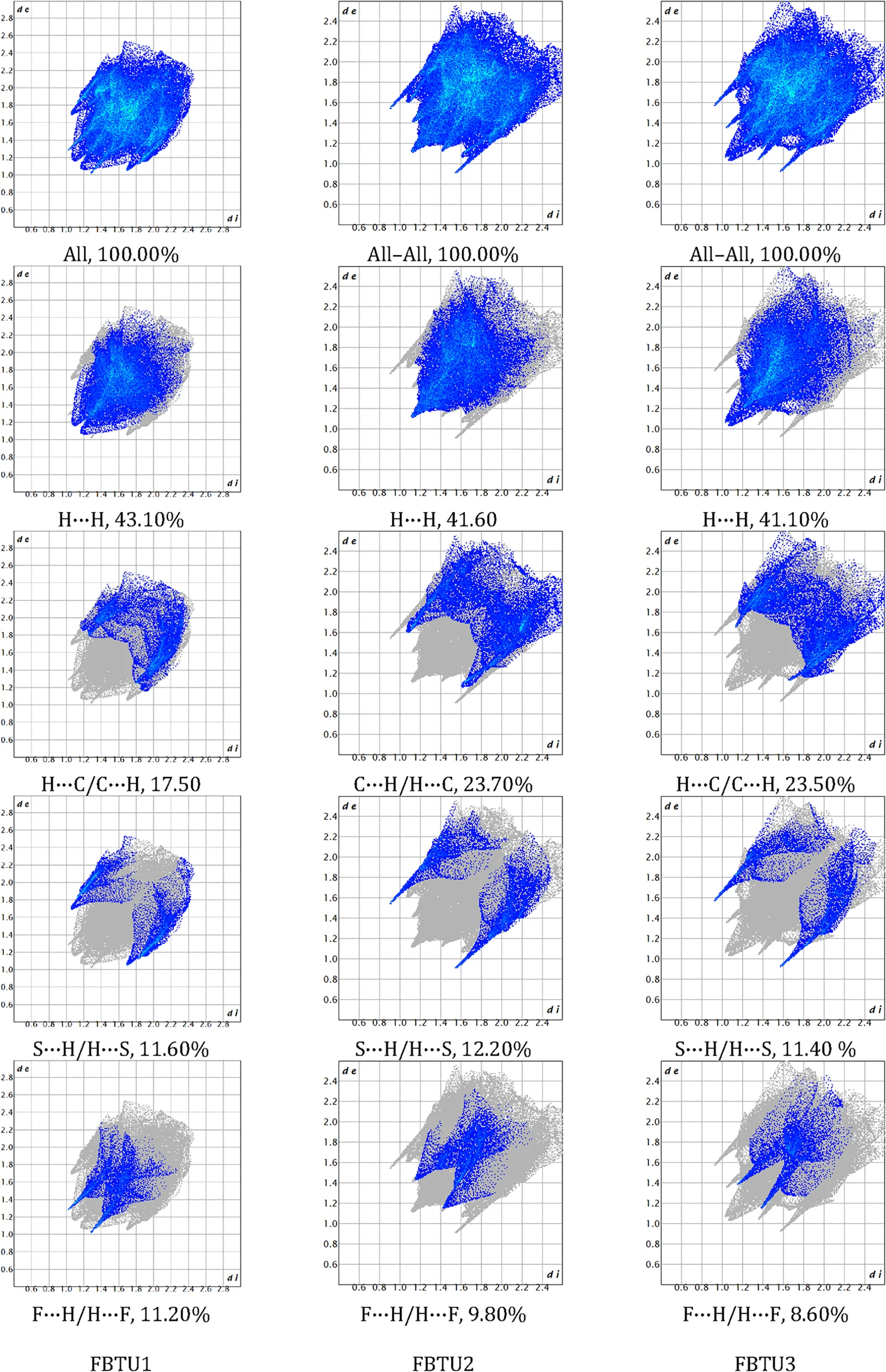
Two–dimensional fingerprint plots showing the major intermolecular contacts contributing to the Hirshfeld surfaces of FBTU1–FBTU3.

A significant contribution is also observed for S···H/H···S contacts, accounting for 11.6, 12.2, and 11.4% in FBTU1, FBTU2, and FBTU3, respectively (Figure 6). These contacts arise primarily from intermolecular N–H···S hydrogen bonds involving the thiocarbonyl sulfur atom and appear as characteristic spikes at short contact distances in the fingerprint plots. The comparable percentages observed throughout the series indicate that the sulfur–centered hydrogen–bonding network remains largely unaffected by the positional variation of the fluorine substituent. The S···H/H···S contributions observed for FBTU1–FBTU3 (11.4–12.2%) are comparable to those reported for related acylthiourea crystals (9.5–12.8%), supporting the persistence of sulfur-centered hydrogen bonding as a characteristic packing feature.^105^ In contrast, the H···H contributions in the present series vary only slightly (41.1–43.1%), whereas substantially broader variations have been reported for related acylthiourea systems, indicating that fluorine positional isomerism produces only limited changes in the overall dispersion-dominated contact environment.^118^

The contribution of F···H/H···F contacts gradually decreases from 11.2% in FBTU1 to 9.8% in FBTU2 and 8.6% in FBTU3 (Figure 6). Although fluorine is generally considered a weak hydrogen–bond acceptor, these contacts indicate that the fluorine substituent participates in short intermolecular interactions that contribute to crystal stabilization through a combination of electrostatic and van der Waals effects. The progressive decrease in F···H/H···F contributions accompanied by the increase in C···H/H···C contacts suggests a subtle redistribution of intermolecular interactions across the series, whereby aromatic contacts become increasingly favored relative to fluorine–associated interactions (Figure 6). The O···H/H···O contacts contribute between 7.4 and 7.8% of the Hirshfeld surfaces and originate from interactions involving the amide carbonyl oxygen atom. The relatively constant contributions observed for these contacts indicate that the role of the carbonyl group in the intermolecular interaction landscape remains essentially unchanged throughout the series.

Consequently, the Hirshfeld surface analyses demonstrate that the crystal packing of FBTU1–FBTU3 is governed by a cooperative interplay of dispersion–driven H···H contacts, aromatic C–H···π interactions, N–H···S hydrogen bonds, fluorine–associated contacts, and secondary O···H interactions. Despite the positional variation of the fluorine substituent, the overall interaction profiles remain remarkably similar, indicating that the dominant supramolecular framework is preserved across the series while only subtle differences in the balance of aromatic and fluorine–centered interactions are introduced.

The interaction energy frameworks computed using the B3LYP model are presented in Figures S19–S21 and the corresponding energy components are tabulated in Tables S3–S5. In all three compounds, dispersion energy (*E*_dis_) is the dominant stabilizing contribution, with total dispersion energies of −204.249, −214.301, and −218.503 kJ/mol for FBTU1, FBTU2, and FBTU3, respectively. The predominance of dispersion interactions is consistent with the Hirshfeld fingerprint analyses, which revealed H···H and C···H/H···C contacts as the major contributors to the intermolecular interaction landscape. Accordingly, the total energy frameworks closely resemble the dispersion frameworks, indicating that van der Waals and π–related interactions play a central role in crystal stabilization. The total lattice energies for FBTU1, FBTU2, and FBTU3 were calculated as −211.928, −220.556, and −216.666 kJ/mol, respectively, indicating comparable crystal stability across the three isomers, with FBTU2 displaying the highest lattice cohesion. The slightly more negative total interaction energy obtained for FBTU2 suggests a marginally more efficient packing arrangement, which is in agreement with the enhanced contribution of C···H/H···C contacts observed in its fingerprint plot. Nevertheless, the relatively small differences between the total energies indicate that the positional variation of the fluorine substituent exerts only a limited influence on the overall energetic stability of the crystal structures.

Analysis of the individual color–coded molecular pairs reveals that the strongest interactions occur at relatively short intermolecular separations (*R* ≈ 5–7 Å). For FBTU1, the most stabilizing interaction corresponds to the dark–blue molecular pair at *R* = 6.15 Å, with *E*_tot_ = −44.84 kJ/mol, followed by the purple–colored pair at *R* = 6.84 Å with *E*_tot_ = −41.16 kJ/mol (Figure 7a and Table S3). Similarly, in FBTU2, the strongest interaction is associated with the blue–colored molecular pair located at *R* = 6.21 Å, exhibiting an interaction energy of *E*_tot_ = −47.08 kJ/mol, while the green–colored pair at *R* = 4.93 Å also contributes significantly to crystal stabilization with *E*_tot_ = −44.42 kJ/mol (Figure 7b and Table S4). In the case of FBTU3, the red–colored molecular pair at *R* = 4.90 Å (*E*_tot_ = −49.65 kJ/mol) and the blue pair at *R* = 6.10 Å (*E*_tot_ = −49.66 kJ/mol) represent the most stabilizing interactions in the crystal structure (Figure 7c and Table S5). In all three compounds, these highly stabilizing molecular pairs are characterized by substantial dispersion energy contributions, confirming that short–range dispersive interactions dominate the crystal packing energetics. Visual inspection of the energy frameworks further confirms that the dispersion networks (green cylinders) dominate the crystal packing, whereas the electrostatic contributions (red cylinders) are primarily associated with the directional N–H···S hydrogen–bonding interactions identified by single–crystal X–ray diffraction analysis. Consequently, the total energy frameworks (blue cylinders) closely resemble the dispersion frameworks, demonstrating that van der Waals and π–related interactions provide the principal energetic contribution to crystal stabilization, while sulfur–centered hydrogen bonds act as key structure–directing interactions within the supramolecular assemblies.

**Figure 7:**
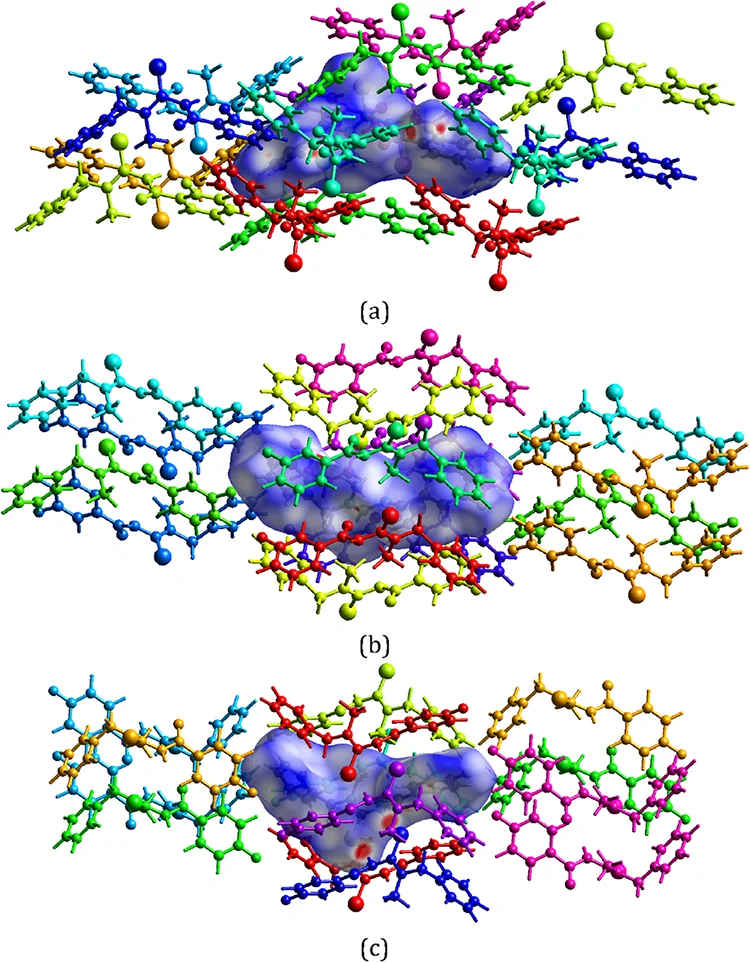
Three–dimensional visualization of the intermolecular energy framework around the reference molecule in FBTU1–FBTU3 (a–c).

### 3.5 DFT Analysis

Density functional theory (DFT) calculations were carried out at the B3LYP/6–311G­(d,p) level of theory to investigate the electronic structure and global chemical reactivity of FBTU1–FBTU3. The optimized geometries are shown in Figure S22, and the calculated global chemical reactivity descriptors are summarized in Table 3. The calculated geometries exhibited good agreement with the experimental X–ray diffraction data, with RMSD values of 0.460 Å for FBTU1, 0.388 Å for FBTU2, and 0.585 Å for FBTU3, indicating the reliability of the applied theoretical model (Figure 8). The frontier molecular orbital (FMO) distributions are illustrated in Figure 9. The HOMO and LUMO densities are primarily distributed over the benzoylthiourea scaffold and partially delocalized onto the benzyl phenyl ring, indicating effective π–conjugation throughout the molecular framework. Such orbital delocalization is expected to facilitate intramolecular charge transfer and contribute to the electronic communication between the thiourea core and the aromatic substituents. The HOMO–LUMO energy gaps were calculated as 3.9805 eV for FBTU1, 3.9424 eV for FBTU2, and 4.0597 eV for FBTU3. Accordingly, FBTU2 possesses the smallest energy gap, suggesting enhanced electronic polarizability and a greater propensity for charge transfer, whereas FBTU3 exhibits the largest energy gap and therefore the highest kinetic stability. The calculated global reactivity descriptors support this trend. FBTU2 displays the lowest chemical hardness (η = 1.9712 eV), the highest softness (σ = 0.2537 eV^–1^), and the highest electrophilicity index (ω = 4.0614 eV), indicating a more reactive electronic structure. In contrast, FBTU3 exhibits the highest hardness (η = 2.0298 eV) and the lowest softness (σ = 0.2463 eV^–1^), reflecting a greater resistance to electronic perturbation. The experimental DPPH radical–scavenging activities follow the order FBTU2 > FBTU3 > FBTU1, which is partially consistent with the calculated reactivity descriptors. The superior antioxidant activity of FBTU2 correlates well with its smallest HOMO–LUMO gap, highest softness, and lowest hardness, suggesting that enhanced charge–transfer capability may facilitate radical–scavenging processes. However, the comparable antioxidant activities of FBTU2 and FBTU3 despite their different HOMO–LUMO gaps indicate that antioxidant behavior cannot be explained solely by frontier orbital energies. Besides electron transfer, the efficiency of DPPH radical quenching is strongly influenced by the stability of the radical intermediate formed after hydrogen atom transfer, the extent of spin–density delocalization, molecular conformation, and solvent–solute interactions.^119,120^ Therefore, although the calculated reactivity descriptors successfully explain the superior performance of FBTU2, the high antioxidant activity of FBTU3 suggests that additional structural and conformational factors also contribute to radical stabilization. In this regard, the *meta*– and *para*–fluoro derivatives appear to provide a more favorable environment for radical stabilization than the *ortho*–substituted analogue.^119,121^

**Figure 8:**
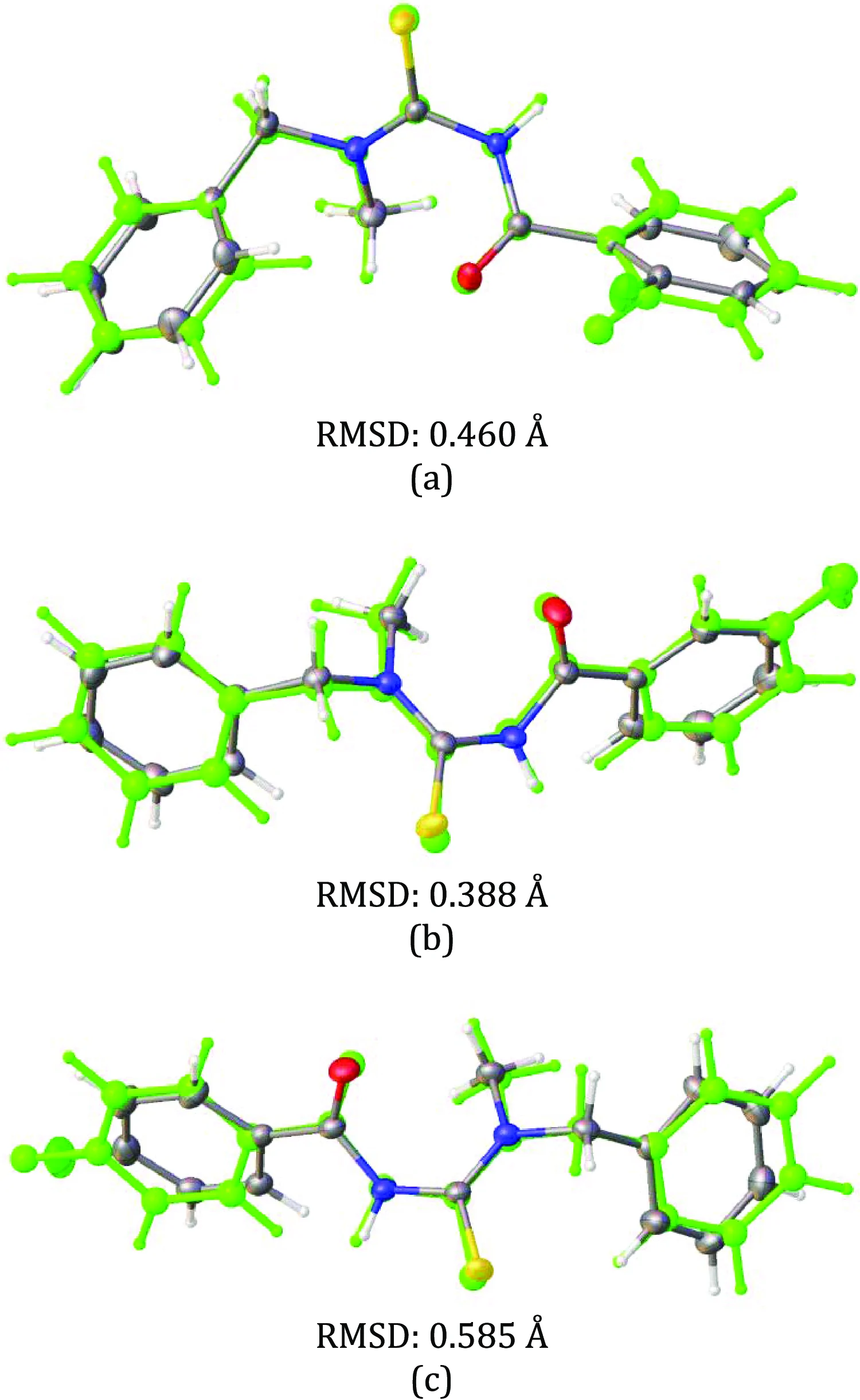
Superposition of the experimental single–crystal X–ray structures (gray) and the DFT–optimized geometries (green) of (a) FBTU1, (b) FBTU2, and (c) FBTU3.

**Figure 9:**
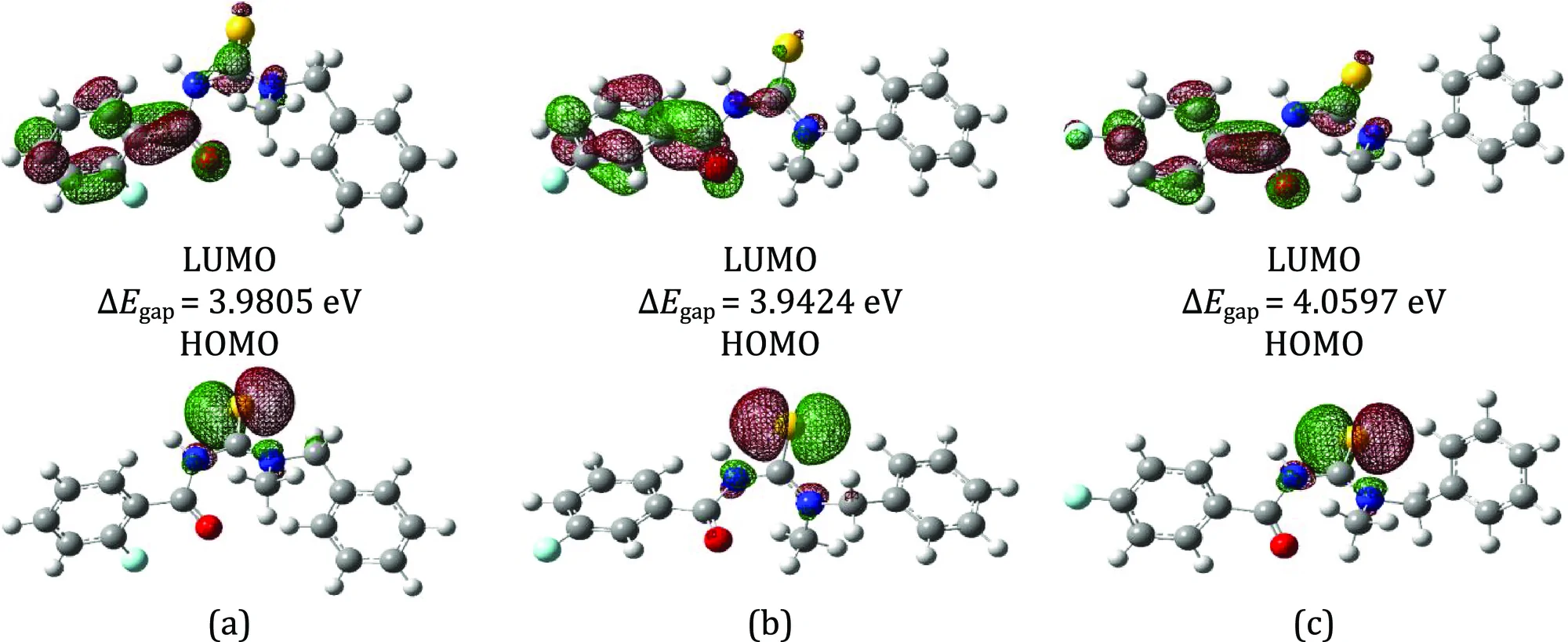
Frontier molecular orbital (HOMO and LUMO) distributions of (a) FBTU1, (b) FBTU2, and (c) FBTU3.

**Table 3:** Global Chemical Reactivity Descriptors and Energy Values Calculated at the B3LYP/6-311G­(d,p) Level for FBTU1–FBTU3

Parameters	FBTU1	FBTU2	FBTU3
SCF energy (a.u.)	–1301.76	–1301.77	–1301.77
Total energy (Thermal) *E*_total_ (kcal/mol)	188.41	188.41	188.47
Heat capacity at const. volume, *C*_v_ (cal/mol·K)	71.268	71.260	71.206
Vibrational energy, *E*_vib_ (kcal/mol)	186.635	186.632	186.696
Zero–point vibrational energy, *E*_o_ (kcal/mol)	176.620	176.676	176.762
Entropy (cal/mol·K)			
Translational	43.013	43.013	43.013
Rotational	34.772	34.885	34.915
Vibrational	68.945	69.185	68.290
Total	146.730	147.083	146.219
Rotational constant (GHz)			
*A*	0.54566	0.80990	0.82950
*B*	0.14148	0.10430	0.10095
*C*	0.12414	0.10124	0.09909
Dipole moment (Debye)			
μ_*x*_	–2.6648	0.0198	–0.0364
μ_*y*_	–0.5624	–0.7586	–2.0182
μ_*z*_	4.0478	4.0426	2.9949
μ_Total_	4.8787	4.1133	3.6116
LUMO energy (eV)	–1.9121	–2.0302	–1.8893
HOMO energy (eV)	–5.8926	–5.9726	–5.9490
Energy gap (eV)	3.9805	3.9424	4.0597
Ionization potential (eV)	5.8926	5.9726	5.9490
Electron affinity (eV)	1.9121	2.0302	1.8893
Chemical hardness (eV)	1.9902	1.9712	2.0298
Global softness (1/eV)	0.2512	0.2537	0.2463
Electronegativity (eV)	3.9024	4.0014	3.9191
Chemical potential (eV)	–3.9024	–4.0014	–3.9191
Electrophilicity (eV)	3.8258	4.0614	3.7834

The Mulliken atomic charge distributions of FBTU1–FBTU3 were calculated at the DFT level, and the results are summarized in Table S6. In all three compounds, the most negative charge is consistently located on the amide nitrogen atom (N4), with calculated charges of −0.3994, −0.4076, and −0.4090 for FBTU1, FBTU2, and FBTU3, respectively. This pronounced negative charge reflects the high electron density associated with the amide–thiourea conjugated framework and highlights N4 as one of the most electron–rich centers within the molecules. The carbonyl oxygen atom (O3) and thiocarbonyl sulfur atom (S1) also carry substantial negative charges, confirming their roles as the primary electron–accepting sites and potential interaction centers in both intermolecular recognition and hydrogen–bond formation.^78^ Among the positively charged atoms, the carbon atom bonded to the heteroatom environment exhibits the highest positive charge in each structure, corresponding to C7 in FBTU1 (+0.4062), C6 in FBTU2 (+0.4253), and C6 in FBTU3 (+0.4214). The pronounced positive character of these carbon atoms originates from the strong electron–withdrawing influence of the adjacent carbonyl and thiocarbonyl functionalities, making them the most electrophilic centers within the molecular frameworks.

The fluorine substituent retains a negative charge in all three isomers, with values ranging from −0.2001 to −0.2251. The slightly more negative charge observed for fluorine in FBTU2 and FBTU3 suggests a modest influence of substitution position on the local electronic environment; however, the differences remain relatively small, indicating that fluorine preserves a similar electronic character throughout the series.^122^ Likewise, the sulfur, oxygen, and nitrogen atoms display only minor variations in charge distribution among the three isomers, demonstrating that positional fluorine substitution exerts only a limited effect on the overall electron–density distribution of the benzoylthiourea skeleton. All hydrogen atoms possess positive charges, as expected, with the most electropositive hydrogen atoms corresponding to H5 in FBTU1 (+0.2727), H36 in FBTU2 (+0.2682), and H5 in FBTU3 (+0.2672). These highly positive hydrogen atoms are associated with heteroatom–containing functional groups and therefore represent the most probable hydrogen–bond donor sites within the molecules. The substantial positive charge localized on these atoms is consistent with the intermolecular N–H···S and N–H···O interactions observed in the crystal structures.

The molecular electrostatic potential (MEP) maps of FBTU1–FBTU3 are presented in Figure 10 and provide a visual representation of the electron–density distribution across the molecular surfaces. In all three compounds, the most negative electrostatic potential regions (red to orange) are localized around the carbonyl oxygen and thiocarbonyl sulfur atoms, identifying these heteroatoms as the most electron–rich sites and the preferred regions for electrophilic attack and intermolecular hydrogen–bond acceptance. Conversely, the most positive electrostatic potential regions (blue) are concentrated around the N–H functionality, indicating its role as the primary hydrogen–bond donor site. These observations are in excellent agreement with the Mulliken population analysis, which identified O3, S1, and N4 as the most negatively charged atoms and the N–H proton as one of the most electropositive centers in the molecules. Although the overall electrostatic potential distributions are similar for the three positional isomers, subtle differences can be observed in the degree of charge separation. Among the investigated compounds, FBTU2 exhibits the most pronounced differentiation between electron–rich and electron–deficient regions, suggesting a greater degree of electronic polarization across the molecular framework.^123^ Such enhanced polarization may facilitate charge redistribution during radical–scavenging processes and contribute to its superior DPPH activity. In contrast, FBTU1 exhibits a comparatively more uniform electrostatic potential distribution, which may be associated with its lower antioxidant performance. Despite displaying a slightly less pronounced charge separation than FBTU2, FBTU3 maintains high radical–scavenging activity, indicating that molecular conformation and radical stabilization effects also contribute significantly to antioxidant efficiency.

**Figure 10:**
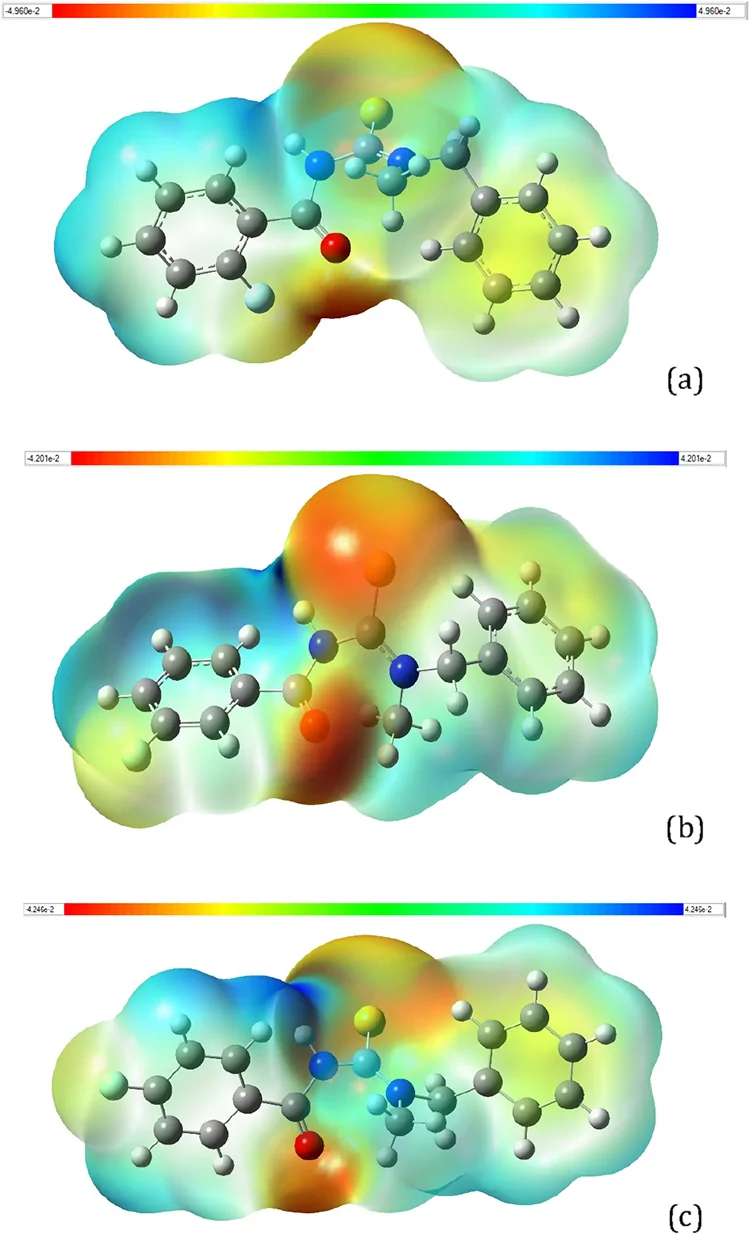
DFT–calculated molecular electrostatic potential (MEP) surfaces of (a) FBTU1, (b) FBTU2, and (c) FBTU3.

The localization of negative electrostatic potential around the O=C–N–C=S fragment further confirms that the benzoylthiourea core serves as the principal reactive region of the molecules. This conclusion is supported by the frontier molecular orbital analysis, where both HOMO and LUMO densities are predominantly distributed over the same conjugated framework. Therefore, the combined MEP and FMO results indicate that the electronic properties of FBTU1–FBTU3 are governed primarily by the benzoylthiourea moiety, while the positional variation of the fluorine substituent modulates the degree of charge polarization rather than altering the location of the principal reactive sites.

### 3.6 ADMET Analysis and Drug–Likeness Evaluation

The in silico pharmacokinetic, physicochemical, and toxicity profiles of the synthesized fluorinated benzoylthiourea derivatives (FBTU1–FBTU3) were comprehensively evaluated using SwissADME^53^ and ProTox–II platforms^60^ to assess their potential as CNS–active drug–like scaffolds targeting monoamine oxidase–B (MAO–B)–related neurodegenerative disorders^28^ (Table 4). Since MAO–B is predominantly localized in the central nervous system and its catalytic activity contributes to oxidative stress through the generation of reactive oxygen species, favorable brain exposure and antioxidant potential are desirable characteristics for prospective anti–Parkinson agents. All compounds exhibited identical global physicochemical descriptors, indicating that fluorine positional isomerism does not significantly affect bulk drug–likeness parameters, but is more likely to influence local electronic distribution and target–specific binding behavior.

**Table 4:**
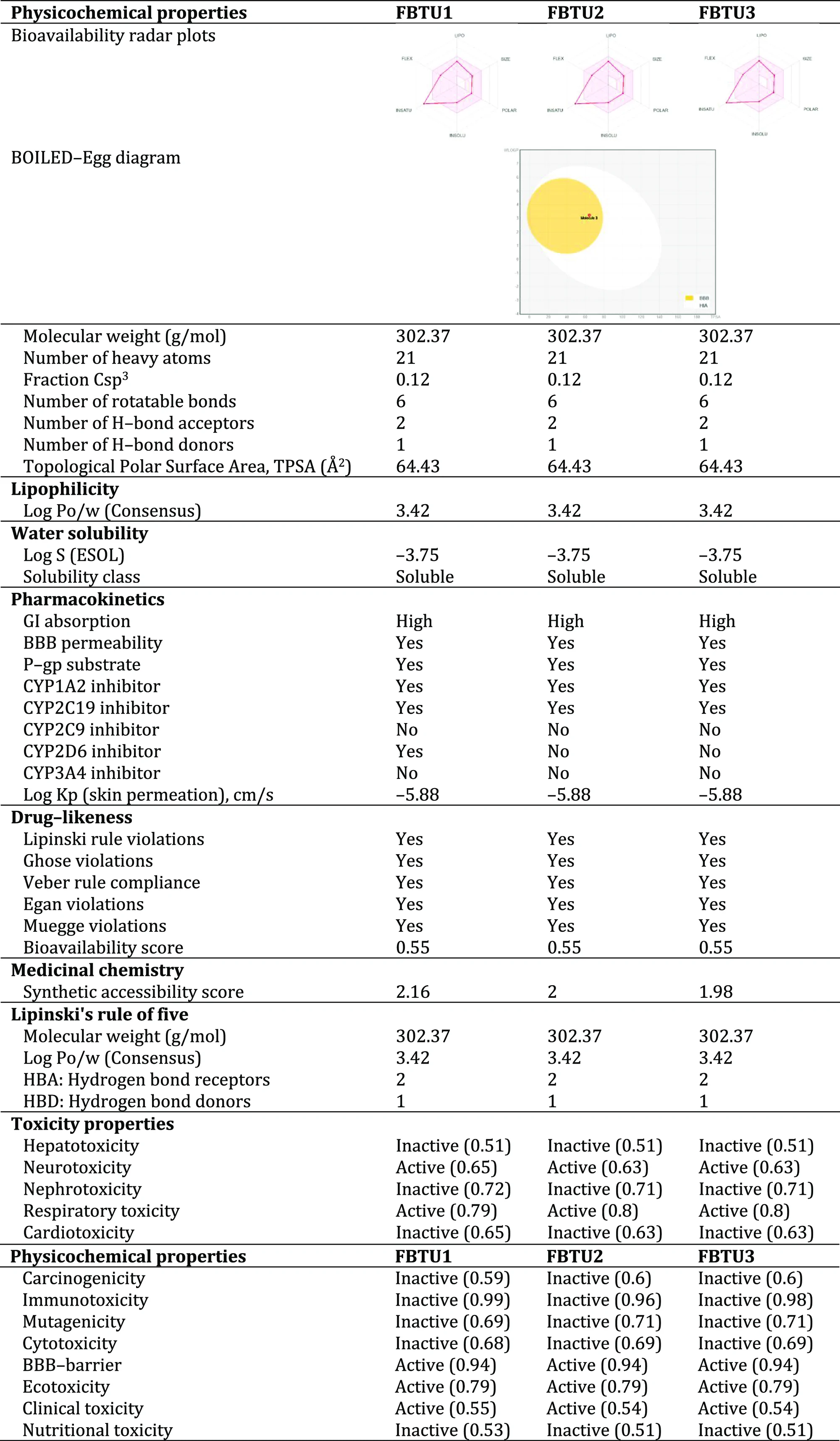
ADMET Properties of FBTU1–FBTU3

The bioavailability radar plots of FBTU1–FBTU3 (Table 4) further support this observation. All three compounds display nearly identical radar profiles, indicating that positional fluorine substitution exerts only a minor influence on their overall physicochemical properties. The red polygons are largely confined within the optimal region defined by the SwissADME model, indicating general agreement with several physicochemical ranges implemented in the SwissADME model. In particular, lipophilicity (LIPO), molecular size (SIZE), polarity (POLAR), flexibility (FLEX), and solubility (INSOLU) fall within the recommended ranges, whereas only a slight deviation is observed for the unsaturation parameter (INSATU), reflecting the highly aromatic and conjugated nature of the benzoylthiourea scaffold. Consistent with these findings, the BOILED–Egg model places all compounds within the BBB–permeable region, suggesting predicted compatibility with BBB penetration within the limitations of the computational model. These predictions should, however, be regarded as preliminary and require experimental pharmacokinetic validation.

The molecular weight (302.37 g/mol), hydrogen–bond donor/acceptor counts (HBD = 1, HBA = 2), rotatable bond number (6), and consensus Log *P* value (3.42) fall within the generally accepted ranges commonly considered in physicochemical drug–likeness assessment.^124^ The TPSA value of 64.43 Å^2^ also falls within a range generally associated with passive membrane permeability.^56^ These findings are highly relevant in the context of Parkinson’s disease because compounds considered for MAO–B-oriented follow-up studies should have properties compatible with brain exposure. The compounds were also predicted to possess acceptable aqueous solubility (Log *S* = −3.75) and a bioavailability score of 0.55, providing additional computational descriptors for their physicochemical profiles. Although all derivatives were identified as P–glycoprotein substrates, which may partially reduce intracellular retention through efflux mechanisms,^125^ the biological significance of these predictions cannot be established without experimental pharmacokinetic studies. Cytochrome P450 predictions indicate inhibition of CYP1A2 and CYP2C19, whereas CYP2C9 and CYP3A4 inhibition was not predicted. Interestingly, only FBTU1 was identified as a potential CYP2D6 inhibitor, suggesting that fluorine substitution position may subtly influence the predicted metabolic interaction profiles.

Toxicity prediction results provided preliminary information on potential toxicity-related end points.^60,126^ All compounds are predicted to be non–hepatotoxic, non–nephrotoxic, non–carcinogenic, non–mutagenic, and non–immunotoxic, within the applied computational model. However, all derivatives show predicted neurotoxicity and respiratory toxicity signals, which may be associated with their predicted BBB permeability and lipophilic–aromatic character. The concurrent BBB positivity (∼0.94 probability) further supports CNS exposure, providing a plausible explanation for the observed neurotoxicity alerts and highlighting the need for experimental validation. Additionally, ecotoxicity predictions indicate potential environmental risk (active ∼0.79), suggesting that although the compounds are potentially useful in early stage pharmacological screening, their environmental persistence and off–target effects should be further investigated in future studies.^54,127^ Other toxicity end points, including cardiotoxicity, hepatotoxicity, and carcinogenicity, remain inactive across all derivatives, in the prediction model.

Taken together, the ADMET calculations provide preliminary computational information on the physicochemical, permeability, metabolic, and toxicity-related characteristics of FBTU1–FBTU3. These predictions are complementary to the experimental antioxidant results and molecular docking analysis but should not be interpreted as evidence of pharmacokinetic suitability or therapeutic potential. Experimental pharmacokinetic, toxicity, and target-based biological studies would be required to establish the biological relevance of these compounds.

### 3.7 Molecular Docking Analysis

Molecular docking studies were performed to investigate the binding interactions and binding affinity profiles of the synthesized benzoylthiourea derivatives against monoamine oxidase–B (MAO–B, PDB ID: 1GOS), an enzyme involved in the oxidative metabolism of monoamines and implicated in the pathophysiology of Parkinson’s disease (PD),^61,63^ to provide a preliminary computational assessment of the possible binding modes and interactions of the synthesized benzoylthiourea derivatives within the MAO–B active site. In this context, the crystal structure of MAO–B was retrieved from the Protein Data Bank and prepared for docking by removing crystallographic water molecules and co–crystallized ligands, followed by the addition of hydrogen atoms and assignment of appropriate protonation states for the docking calculations. The docking protocol was applied to evaluate the binding modes of FBTU1–FBTU3 within the active site of the enzyme. The docking results, including binding energies and docking-derived estimated inhibition constants, are summarized in Table 5, while the detailed protein–ligand interaction profiles are presented in Table S7. The three–dimensional binding poses and corresponding two–dimensional interaction diagrams are illustrated in Figure S23. The results indicate that all synthesized ligands are predicted to accommodate the MAO–B active site through a combination of hydrogen bonding, hydrophobic interactions, halogen contacts, and π–related noncovalent interactions.

**Table 5:** Molecular Docking and Binding Energy Analysis of the Studied Ligands against 1GOS

Ligand	Run	Cluster Rank	Cluster Size (n)	Binding Energy (kcal/mol)	Calculated *K*_i_ (μM)	Intermolecular Energy (kcal/mol)	vdW + Hbond + Desolv (kcal/mol)	Electrostatic (kcal/mol)	Torsional Energy (kcal/mol)
FBTU1	47	1	19	–8.12	1.13	–9.61	–9.48	–0.13	1.49
FBTU2	74	1	10	–7.62	2.61	–9.11	–8.92	–0.19	1.49
FBTU3	90	1	3	–7.56	2.89	–9.05	–9.04	–0.01	1.49

Among the studied compounds, FBTU1 exhibited the most favorable calculated docking energy of −8.12 kcal/mol and an estimated inhibition constant (*K*_i_) of 1.13 μM. In contrast, FBTU2 and FBTU3 showed less favorable calculated docking energies of −7.62 and −7.56 kcal/mol, respectively, suggesting that subtle structural variations influence their predicted binding modes within the enzyme pocket.^64,128^

For all three compounds, the dominant contribution to intermolecular energy arose from van der Waals, hydrogen–bonding, and desolvation terms (−9.05 to −9.61 kcal/mol), with minor electrostatic contributions (−0.01 to −0.19 kcal/mol), consistent with a primarily hydrophobic binding mode. FBTU1 exhibited hydrogen-bonding interactions with SER59, LYS296, and GLY58, together with π-associated contacts involving TYR60 and TYR435. FBTU2 showed a simpler interaction profile dominated by hydrogen bonding with GLU34 and SER394, whereas FBTU3 formed hydrogen bonds with ARG42 and TYR398 together with a halogen-related contact with LEU56. These differences indicate that the fluorine substitution position influences the predicted interaction pattern within the MAO–B binding site.

The docking results indicate that the positional variation of fluorine affects the predicted interaction profiles of FBTU1–FBTU3 within the MAO–B active site, consistent with the recognized influence of ligand substitution and binding orientation on protein–ligand interactions.^82,129,130^ The ortho-fluorine position in FBTU1 may facilitate an orientation that allows both hydrogen-bonding and halogen-related interactions within the modeled active site.^131^ However, these docking results represent a preliminary computational assessment and should not be interpreted as evidence of MAO–B inhibition or therapeutic activity. Experimental enzyme inhibition studies are required to determine whether the predicted binding differences translate into measurable MAO–B activity.

### 3.8 DPPH Free Radical Scavenging Assay

In this study, the DPPH (1,1–diphenyl–2–picrylhydrazyl) assay was employed to evaluate the antioxidant potential of the newly synthesized thiourea derivatives. DPPH is a stable free radical that can be neutralized through hydrogen atom or electron donation from antioxidant molecules, resulting in a decrease in absorbance that reflects the radical–quenching capacity of the tested compounds.^132,133^ Therefore, the obtained results provide information regarding chemical radical–scavenging ability rather than direct antioxidant activity under biological conditions. The free radical scavenging activities of the compounds against DPPH radicals were assessed at five different concentrations: 1000, 500, 250, 125, and 62.5 μg/mL. Ascorbic acid, a well–known antioxidant, was used as a positive control under identical experimental conditions for comparative evaluation.^134^

As presented in Figure 11, all tested compounds exhibited dose–dependent radical scavenging activity with increasing concentration.^72^ At the highest concentration tested (1000 μg/mL), FBTU1, FBTU2, and FBTU3 showed inhibition percentages of 88.1, 94.7, and 94.6%, respectively. Under the same conditions, ascorbic acid displayed the strongest antioxidant activity, with an inhibition value of 96.9%. At 500 μg/mL, the inhibition percentages were 81.0% for FBTU1, 93.7% for FBTU2, 90.6% for FBTU3, and 96.8% for ascorbic acid. At 250 μg/mL, FBTU1, FBTU2, and FBTU3 exhibited inhibition values of 68.6, 90.6, and 83.4%, respectively, whereas ascorbic acid maintained its high activity with an inhibition percentage of 96.4%. Although a slight decrease in the activity of ascorbic acid was observed at lower concentrations (95.8%), it remained superior to all tested compounds. In contrast, at 125 μg/mL, FBTU1, FBTU2, and FBTU3 showed inhibition percentages of 51.1, 68.3, and 74.4%, respectively, while at 62.5 μg/mL, the inhibition values were 46.6, 57.9, and 62.6%, respectively.

**Figure 11:**
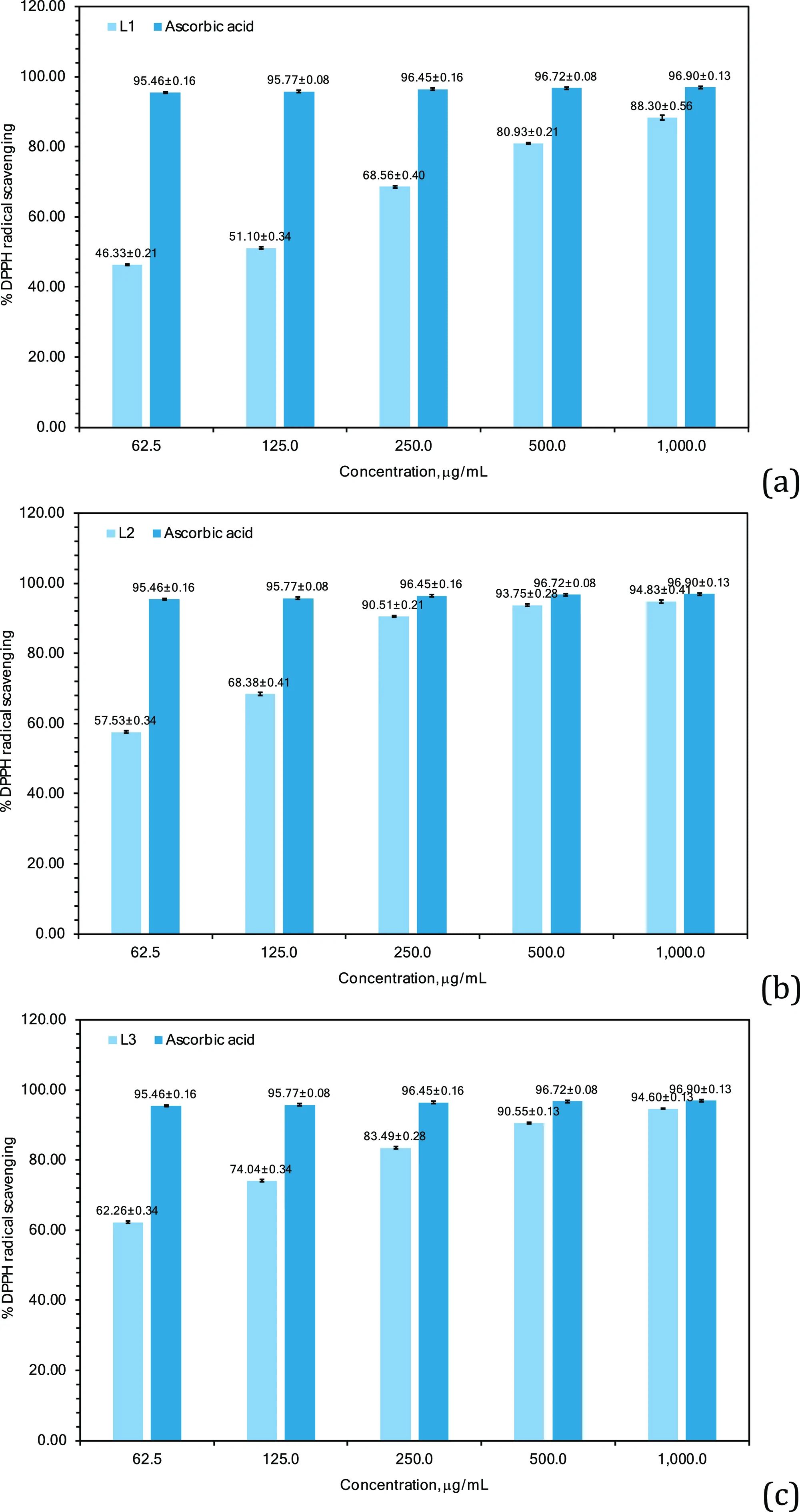
Concentration–dependent DPPH radical scavenging activities of FBTU1 (a), FBTU2 (b), FBTU3 (c), and the reference antioxidant ascorbic acid.

The antioxidant properties are primarily governed by the common *N*–(benzyl­(methyl)­carbamothioyl)­fluorobenzamide framework, whereas the position of the fluorine substituent fine–tunes the radical–scavenging efficiency. The very similar activities of FBTU2 and FBTU3 indicate that *meta*– and *para*–fluoro substitution provide comparable environments for radical stabilization. In contrast, the lower activity of the *ortho*–substituted derivative (FBTU1) may arise from steric effects associated with the proximity of the fluorine atom to the benzoylthiourea core, which can slightly reduce the accessibility of the active hydrogen–donor sites and limit efficient stabilization of the resulting radical species.^135–137^

Comparison with previously reported thiourea and fluorinated thiourea derivatives further highlights the promising antioxidant performance of the present compounds. Structurally related thiourea derivatives generally exhibit moderate–to–good DPPH radical–scavenging activities, typically ranging from 55 to 85% inhibition at comparable concentrations.^18,31,138^ In contrast, FBTU2 and FBTU3 displayed radical–scavenging efficiencies exceeding 90% at higher concentrations and compare favorably with related fluorinated thiourea systems reported in the literature.^72,139^ These findings further support the fluorinated benzoylthiourea scaffold as a promising platform for the development of antioxidant agents. Considering that MAO–B–mediated dopamine metabolism generates reactive oxygen species that contribute to oxidative stress and neuronal damage in Parkinson’s disease, the pronounced radical–scavenging activity observed for FBTU2 and FBTU3 may provide complementary neuroprotective benefits alongside the predicted MAO–B binding properties of this compound series. Such a combined antioxidant/predicted MAO–B-binding profile may justify further biological testing in Parkinson’s disease-oriented models.

The DPPH trend observed for FBTU1-FBTU3 can be further considered in relation to the fluoro-substituted benzoylthioureas reported by Joha et al.^18^ In that study, the *N*-(4-methoxy-2-methylphenyl)­carbamothioyl series showed a clear positional dependence in the DPPH assay: T2 was the most active derivative, reaching 94.3% inhibition at 1000 μM, followed closely by T3 (94.0%), whereas the ortho-fluoro derivative T1 was less active (76.2%). The same order was retained at lower concentrations, for example at 500 μM (T1/T2/T3 = 53.1/89.4/75.6%) and 250 μM (36.4/75.6/57.5%). The two series show a broadly similar positional trend, with the *ortho*-fluoro derivative being the least active and the *meta*/*para*-fluoro derivatives exhibiting stronger radical-scavenging activity. In the present *N*-benzyl-*N*-methyl FBTU series, FBTU2 and FBTU3 display the highest DPPH inhibition values (94.7 and 94.6% at 1000 μg/mL; 93.7 and 90.6% at 500 μg/mL), while FBTU1 remains the least active member of the series. Accordingly, this comparison is most informative for evaluating positional trends within each series. Nevertheless, the parallel decrease in activity for the *ortho*-fluoro derivative supports the view that steric effects near the benzoylthiourea core can reduce the accessibility or effectiveness of radical-quenching sites. At the same time, the relatively high activity retained by FBTU1 compared with T1 suggests that replacing the aryl *N*-substituent with an *N*-benzyl-*N*-methyl fragment modifies the steric and electronic environment around the thiourea unit, leading to a narrower activity difference among the three positional isomers. Thus, this comparison indicates that both the fluorine position and the thiourea *N*-substitution pattern contribute to the antioxidant response of fluorinated benzoylthiourea derivatives.

Based on the present experimental and computational findings, a proposed structure–activity relationship (SAR) interpretation is summarized in Figure 12. Within this proposed interpretation, the observed antioxidant behavior and predicted MAO–B-related profiles appear to be influenced by the cooperative contribution of four key structural motifs. The fluorinated aromatic ring modulates the electronic distribution and molecular conformation through positional fluorine substitution, thereby influencing crystal packing, molecular recognition, and MAO–B binding orientation. The central acylthiourea core (O=C–NH–C=S) represents an important structural motif, providing an extended electron-delocalized framework together with hydrogen-bond donor and acceptor sites that may contribute to radical stabilization and predicted enzyme recognition. The thiourea NH group acts as a hydrogen-bond donor, contributing to the formation of intermolecular N–H···S interactions in the crystal lattice while also supporting the ligand–protein hydrogen-bonding pattern predicted within the MAO–B active site and contributing to radical-scavenging behavior. In contrast, the benzyl–*N*–methyl fragment increases the hydrophobic character of the molecules, promoting favorable hydrophobic interactions within the MAO–B binding cavity while supporting lipophilicity and predicted blood–brain barrier permeability. Collectively, these structural features provide a balanced combination of crystal stability, electronic delocalization, antioxidant activity, and predicted MAO–B inhibitory potential, demonstrating that positional fluorination fine-tunes biological performance without altering the common benzoylthiourea pharmacophore.

**Figure 12:**
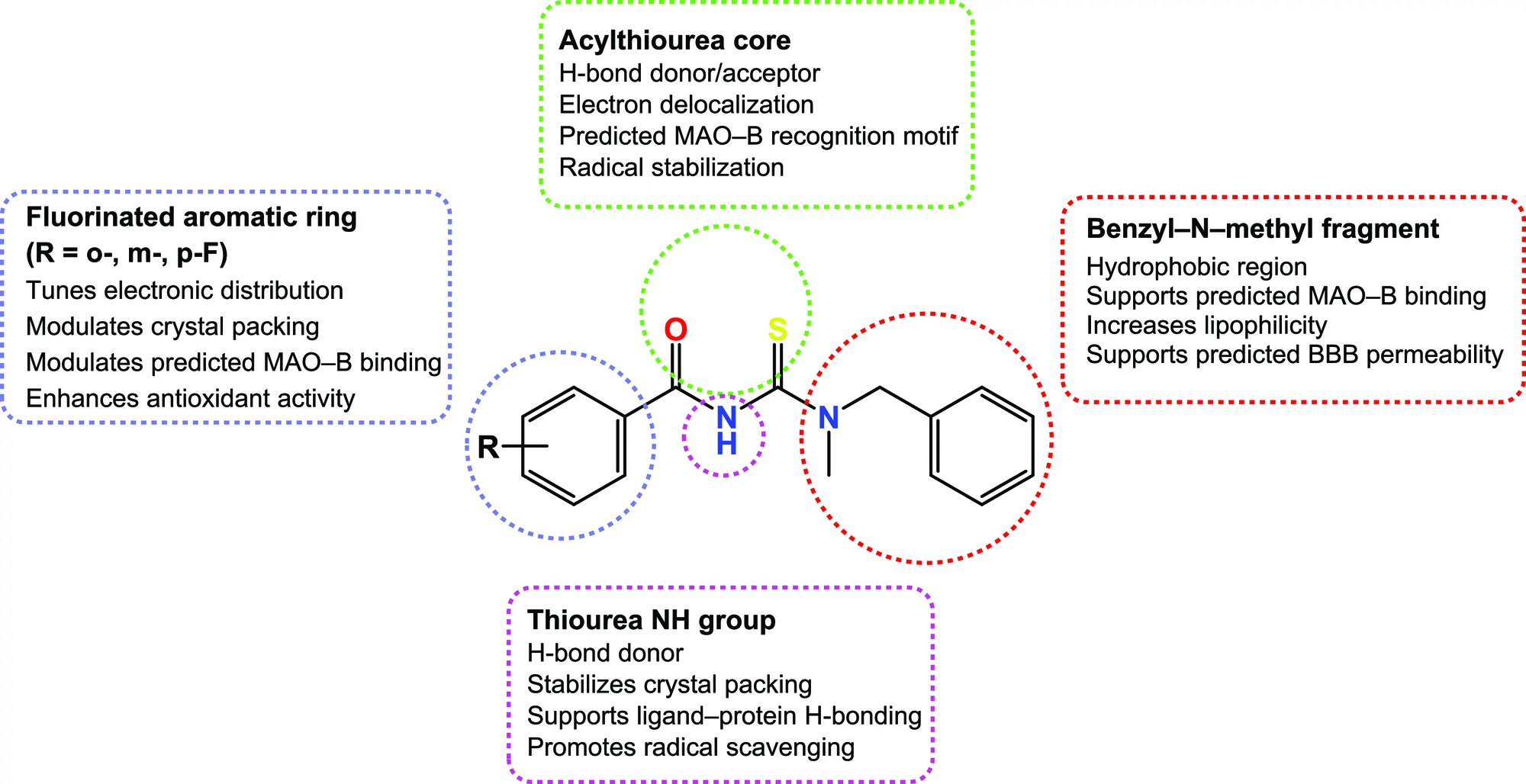
Proposed structure–activity relationship summarizing the possible influence of key structural motifs on the antioxidant activity and predicted MAO-B-related binding profile of fluorinated benzoylthiourea derivatives.

## 4 Conclusion

In summary, three novel fluorinated benzoylthiourea derivatives (FBTU1–FBTU3) were successfully synthesized and comprehensively characterized by spectroscopic techniques and single–crystal X–ray diffraction. All three compounds crystallize in the monoclinic system and exhibit closely related structural features, with the crystal packing primarily stabilized by N–H···S hydrogen bonds together with auxiliary π···π and C–H···π interactions. Hirshfeld surface and energy framework analyses revealed that dispersion forces constitute the dominant contribution to crystal stabilization, with FBTU2 exhibiting the highest lattice cohesion. DFT calculations reproduced the experimental geometries with good accuracy and indicated that FBTU2 possesses the most favorable electronic reactivity profile, as reflected by its smallest HOMO–LUMO energy gap, lowest hardness, and highest softness values. Considering the recognized contribution of oxidative stress to Parkinson’s disease progression, the antioxidant properties of the synthesized compounds were also evaluated. FBTU2 and FBTU3 displayed superior DPPH radical–scavenging activity compared with FBTU1, suggesting that the *meta*– and *para*–fluoro substitution patterns provide a more favorable environment for radical stabilization. ADMET analysis provided computationally predicted physicochemical properties consistent with oral exposure, gastrointestinal absorption, and blood–brain barrier permeability, supporting further experimental evaluation in a CNS-oriented context. Molecular docking studies against monoamine oxidase–B (MAO–B) revealed that FBTU1 showed the most favorable calculated docking energy, whereas FBTU2 demonstrated the most favorable antioxidant and electronic reactivity characteristics. Taken together, the combined experimental and theoretical findings suggest that fluorinated benzoylthiourea derivatives represent structurally characterized scaffolds warranting further experimental evaluation, with FBTU1 standing out as a candidate for further MAO–B-focused experimental validation and FBTU2 emerging as the most attractive derivative from antioxidant and electronic reactivity perspectives. Future studies should first determine the experimental MAO–B inhibitory activities of FBTU1–FBTU3 and evaluate their effects in relevant cellular models to establish whether the predicted binding differences translate into measurable biological responses. Systematic modification of the *N*-benzyl-*N*-methyl fragment, including the evaluation of alternative *N*-substitution patterns as well as mono-, di-, and trifluorinated analogues with different fluorine substitution patterns, could help clarify how the number and position of fluorine atoms influence antioxidant activity, physicochemical properties, and MAO–B recognition. Derivatives showing sufficient activity in enzyme and cellular assays could subsequently be investigated through experimental permeability and blood–brain barrier transport studies, pharmacokinetic evaluation, and preliminary in vivo safety and efficacy assessments.

## Supplementary Material



## Data Availability

Samples of the compounds are available from the author.

## Notes

Single–crystal X–ray diffraction (SC–XRD) data, ^1^H NMR, ^13^C NMR, UV–Vis and FT–IR spectra, together with supplementary molecular docking, Hirshfeld surface analysis and DFT calculation results associated with this article, are available in the online version (PDF)

## References

[1] Poewe W., Seppi K., Tanner C. M., Halliday G. M., Brundin P., Volkmann J., Schrag A.–E., Lang A. E. (2017). Parkinson Disease. Nat. Rev. Dis. Primers.

[2] Lee H., Elkamhawy A., Rakhalskaya P., Lu Q., Nada H., Quan G., Lee K. (2024). Small Molecules in Parkinson’s
Disease Therapy: From Dopamine Pathways to New Emerging Targets. Pharmaceuticals.

[3] Bloem B. R., Okun M. S., Klein C. (2021). Parkinson’s
Disease. Lancet.

[4] Kalia L. V., Asis A., Arbour N., Bar–Or A., Bove R., Di Luca D. G., Fon E. A., Fox S., Gan–Or Z., Gommerman J. L., Kang U. J., Klawiter E. C., Koch M., Kolind S., Lang A. E., Lee K. K., Lincoln M. R., MacDonald P. A., McKeown M. J., Mestre T. A., Miron V. E., Ontaneda D., Rousseaux M. W. C., Schlossmacher M. G., Schneider R., Stoessl A. J., Oh J. (2024). Disease–Modifying Therapies
for Parkinson Disease: Lessons from Multiple Sclerosis. Nat. Rev. Neurol..

[5] Hu B., Wang X., Lu S., Ying X. (2025). A Study of Bidirectional
Control of Parkinson’s Beta Oscillations by Basal Ganglia. Chaos, Solit. Fractals.

[6] Du D., Fu W., Su S., Mao X., Yang L., Xu M., Yuan Y., Gao Y., Geng Z., Chen Y., Zhao M., Fu Y., Yin F., Han H. (2025). Remote Regulation
of Molecular Diffusion in Extracellular Space of Parkinson’s
Disease Rat Model by Subthalamic Nucleus Deep Brain Stimulation. Cyborg Bionic Syst..

[7] Pei G., Hu M., Ouyang J., Jin Z., Wang K., Meng D., Wang Y., Chen K., Wang L., Cao L.-Z., Funahashi S., Yan T., Fang B. (2025). Enhancing Attention
Network Spatiotemporal Dynamics for Motor Rehabilitation in Parkinson’s
Disease. Cyborg Bionic Syst..

[8] Hu B., Ye P., Zhang Y., Nan T., Shi X. (2026). Multimodal Bifurcation
Control of Parkinsonian Beta Oscillations by Pedunculopontine Nucleus
Pathways: A Unified Computational Framework Revealing Dynamic Therapeutic
Targets. Electron. Res. Arch..

[9] Zahra U., Saeed A., Abdul Fattah T., Flörke U., Erben M. F. (2022). Recent Trends in Chemistry, Structure,
and Various
Applications of 1–Acyl–3–Substituted Thioureas:
A Detailed Review. RSC Adv..

[10] Ullah S. A., Saeed A., Azeem M., Haider M. B., Erben M. F. (2024). Exploring
the Latest Trends in Chemistry, Structure, Coordination, and Diverse
Applications of 1–Acyl–3–Substituted Thioureas:
A Comprehensive Review. RSC Adv..

[11] Souldozi A., Ramazani A., Dadrass A. R., Ślepokura K., Lis T. (2012). Efficient One-Pot Synthesis of Alkyl
2-(Dialkylamino)-4-Phenylthiazole-5-Carboxylates
and Single-Crystal X-Ray Structure of Methyl 2-(Diisopropylamino)-4-Phenylthiazole-5-Carboxylate. Helv. Chim. Acta.

[12] Rauf M. K., Talib A., Badshah A., Zaib S., Shoaib K., Shahid M., Flörke U., Imtiaz-ud-Din, Iqbal J. (2013). Solution-Phase Microwave
Assisted Parallel Synthesis of N,N’-Disubstituted Thioureas
Derived from Benzoic Acid: Biological Evaluation and Molecular Docking
Studies. Eur. J. Med. Chem..

[13] Singh R. K., Mandal T., Balsubramanian N., Viaene T., Leedahl T., Sule N., Cook G., Srivastava D. K. (2011). Histone
Deacetylase Activators: N-Acetylthioureas Serve as Highly Potent and
Isozyme Selective Activators for Human Histone Deacetylase-8 on a
Fluorescent Substrate. Bioorg. Med. Chem. Lett..

[14] Saeed S., Rashid N., Jones P. G., Ali M., Hussain R. (2010). Synthesis,
Characterization and Biological Evaluation of Some Thiourea Derivatives
Bearing Benzothiazole Moiety as Potential Antimicrobial and Anticancer
Agents. Eur. J. Med. Chem..

[15] Deshmukh B. (2021). Drug Design
and Medicinal Chemistry of Thiourea Derivatives: A Review. Int. J. Sci. Res. Sci. Technol..

[16] Agili F. A. (2024). Biological
Applications of Thiourea Derivatives: Detailed Review. Chemistry.

[17] Saeed A., Mustafa M. N., Zain-ul-Abideen M., Shabir G., Erben M. F., Flörke U. (2019). Current Developments
in Chemistry, Coordination, Structure
and Biological Aspects of 1–(Acyl/Aroyl)–3– (Substituted)­Thioureas:
Advances Continue. J. Sulphur Chem..

[18] Joha T., Solmaz U., Dogen A., Arslan H. (2026). Fluoro–Substituted
Benzoylthiourea Derivatives: Synthesis, Structural Characterization,
and Evaluation of Antioxidant and Antimicrobial Activities. J. Mol. Struct..

[19] Zborovskii Y., Orysyk V., Orysyk S., Vovk M. (2025). Functionalized Thioureas
in Medicinal Chemistry: Binding Ability of Thiourea Derivatives to
Biological Targets, Focused on Their Anticancer Activity. Biointerface Res. Appl. Chem..

[20] Zaman H., Saeed A., Ismail H., Rashid M. (2025). Unveiling the Cyclopropyl
Appended Acyl Thiourea Derivatives as Antimicrobial, α–Amylase
and Proteinase K Inhibitors: Design, Synthesis, Biological Evaluation,
Molecular Docking, DFT and ADMET Studies. Arch.
Biochem. Biophys..

[21] Dnkm A., Al-Abbassi A., Erhayem M. (2024). Synthesis and Characterization
of
Silver Nanoparticles Using Amberlite LA–2 Functionalized with
Benzoyl Thiourea as a Capping Agent. Chem. Afr..

[22] Tüfekyapan T., Bastemur G. Y., Bıçak B., Akpinar R., Sanli N. O., Gündüz S. K., Ozkorucuklu S. P., Karipcin F. (2025). Syntheses of New Ternary Complexes
with 1–Benzoyl–3–(Benzimidazoyl)­Thiourea)
and Amino Acids: Experimental, Theoretical, Electrochemical, Antioxidant,
Antibacterial, and Molecular Docking Studies. Inorg. Chem. Commun..

[23] Saleem
Khan M., Asif Nawaz M., Jalil S., Rashid F., Hameed A., Asari A., Mohamad H., Ur Rehman A., Iftikhar M., Iqbal J., Al–Rashida M. (2022). Deep Eutectic
Solvent Mediated Synthesis of 3,4–Dihydropyrimidin–2­(1H)–Ones
and Evaluation of Biological Activities Targeting Neurodegenerative
Disorders. Bioorg. Chem..

[24] Chew Z. X., Lim C. L., Ng K. Y., Chye S. M., Ling A. P. K., Koh R. Y. (2023). The Role of Monoamine
Oxidase B Inhibitors in the Treatment
of Parkinson’s Disease – an Update. CNS Neurol. Disord. Drug Targets.

[25] Salauddin, Zaidi S. A. A., Ubaid M., Shamim S., Naim M. J., Khanna S., Alam O. (2026). Parkinson’s
Disease: A Progressive Neurodegenerative Disorder and Structure–Activity
Relationship of MAO Inhibitor Scaffolds as an Important Therapeutic
Regimen. CNS Neurol. Disord. Drug Targets.

[26] Dinh
Thanh N., Son Hai D., Ngoc Toan V., Thi Kim Van H., Thi Kim Giang N., Minh Tri N. (2024). Sulfonyl Thioureas
with a Benzo­[d]­Thiazole Ring as Dual Acetylcholinesterase/Butyrylcholinesterase
and Human Monoamine Oxidase A and B Inhibitors: An in Vitro and in
Silico Study. Arch. Pharm..

[27] Meng L.-C., Chen J.-Y., Xu L.-Y., Wang J., Jiang Z.-G., Jin Z., Zhan C.-H. (2026). Schiff
Base-Wrapped Co, Zn-Based Clusters with Hierarchical
Chiral Structures for Therapeutic Application in Parkinson’s
Disease. Inorg. Chem..

[28] Youdim M. B. H., Edmondson D., Tipton K. F. (2006). The Therapeutic Potential of Monoamine
Oxidase Inhibitors. Nat. Rev. Neurosci..

[29] Ostadkarampour M., Putnins E. E. (2021). Monoamine
Oxidase Inhibitors: A Review of Their Anti–Inflammatory
Therapeutic Potential and Mechanisms of Action. Front. Pharmacol..

[30] Yilmaz E. A., Solmaz U., Arslan H. (2026). Experimental and Theoretical
Analyses
of the Structural Composition and Supramolecular Structures of Selenoureas:
Evaluating Antioxidant Activity. J. Mol. Struct..

[31] Solmaz U. (2026). Design of
rutheniumIIN–Arylthiourea Complex: Structural Elucidation,
Supramolecular Assembly, Hirshfeld Analysis, Antioxidant Evaluation,
and Molecular Docking with Glutathione Reductase. J. Mol. Struct..

[32] Saeed A., Erben M. F., Flörke U. (2010). Effect of
Fluorine Substitution on
the Crystal Structures and Vibrational Properties of Phenylthiourea
Isomers. J. Mol. Struct..

[33] Saeed A., Shaheen U., Hameed A., Naqvi S. Z. H. (2009). Synthesis, Characterization
and Antimicrobial Activity of Some New 1-(Fluorobenzoyl)-3-(Fluorophenyl)­Thioureas. J. Fluor. Chem..

[34] Saeed A., Erben M. F., Shaheen U., Flörke U. (2011). Synthesis,
Structural and Vibrational Properties of 1-(4-Fluorobenzoyl)-3-(Isomeric
Fluorophenyl)­Thioureas. J. Mol. Struct..

[35] Douglass I. B., Dains F. B. (1934). Some Derivatives
of Benzoyl and Furoyl Isothiocyanates
and Their Use in Synthesizing Heterocyclic Compounds1. J. Am. Chem. Soc..

[36] Bruker . APEX III; Bruker AXS Inc.: Madison, Wisconsin, USA, 2019.

[37] Sheldrick G. M. (2015). SHELXT-Integrated
Space–Group and Crystal–Structure Determination. Acta Crystallogr. Sect. A Found. Adv..

[38] Sheldrick G. M. (2015). Crystal
Structure Refinement with SHELXL. Acta Crystallogr.
Sect. C Struct. Chem..

[39] Dolomanov O. V., Bourhis L. J., Gildea R. J., Howard J. A. K., Puschmann H. (2009). *OLEX2*: A Complete Structure Solution,
Refinement and Analysis Program. J. Appl. Crystallogr..

[40] Macrae C. F., Edgington P. R., McCabe P., Pidcock E., Shields G. P., Taylor R., Towler M., van de Streek J. (2006). *Mercury*: Visualization and Analysis of Crystal Structures. J. Appl. Crystallogr..

[41] Macrae C. F., Sovago I., Cottrell S. J., Galek P. T. A., McCabe P., Pidcock E., Platings M., Shields G. P., Stevens J. S., Towler M., Wood P. A. (2020). Mercury
4.0: From Visualization to
Analysis, Design and Prediction. J. Appl. Crystallogr..

[42] Spackman P. R., Turner M. J., McKinnon J. J., Wolff S. K., Grimwood D. J., Jayatilaka D., Spackman M. A. (2021). CrystalExplorer: A Program for Hirshfeld
Surface Analysis, Visualization and Quantitative Analysis of Molecular
Crystals. J. Appl. Crystallogr..

[43] McKinnon J. J., Jayatilaka D., Spackman M. A. (2007). Towards Quantitative Analysis of
Intermolecular Interactions with Hirshfeld Surfaces. Chem. Commun..

[44] Jayatilaka, D. ; Grimwood, D. J. Tonto: A FORTRAN Based Object–Oriented System for Quantum Chemistry and Crystallography. In Lecture Notes in Computer Science; Springer: Berlin, Heidelberg, 2003; pp 142–151.

[45] Frisch, M. J. ; Trucks, G. W. ; Schlegel, H. B. ; Scuseria, G. E. ; Robb, M. A. ; Cheeseman, J. R. ; Scalmani, G. ; Barone, V. ; Petersson, G. A. ; Nakatsuji, H. ; Li, X. ; Caricato, M. ; Marenich, A. V. ; Bloino, J. ; Janesko, B. G. ; Gomperts, R. ; Mennucci, B. ; Hratchian, H. P. ; Ortiz, J. V. ; Izmaylov, A. F. ; Sonnenberg, J. L. ; Williams–Young, D. ; Ding, F. ; Lipparini, F. ; Egidi, F. ; Goings, J. ; Peng, B. ; Petrone, A. ; Henderson, T. ; Ranasinghe, D. ; Zakrzewski, V. G. ; Gao, J. ; Rega, N. ; Zheng, G. ; Liang, W. ; Hada, M. ; Ehara, M. ; Toyota, K. ; Fukuda, R. ; Hasegawa, J. ; Ishida, M. ; Nakajima, T. ; Honda, Y. ; Kitao, O. ; Nakai, H. ; Vreven, T. ; Throssell, K. ; Montgomery, J. A. Jr. ; Peralta, J. E. ; Ogliaro, F. ; Bearpark, M. J. ; Heyd, J. J. ; Brothers, E. N. ; Kudin, K. N. ; Staroverov, V. N. ; Keith, T. A. ; Kobayashi, R. ; Normand, J. ; Raghavachari, K. ; Rendell, A. P. ; Burant, J. C. ; Iyengar, S. S. ; Tomasi, J. ; Cossi, M. ; Millam, J. M. ; Klene, M. ; Adamo, C. ; Cammi, R. ; Ochterski, J. W. ; Martin, R. L. ; Morokuma, K. ; Farkas, O. ; Foresman, J. B. ; Fox, D. J. Gaussian 16, Revision C.01; Gaussian Inc.: Wallingford CT, 2016.

[46] Spackman M. A., Jayatilaka D. (2009). Hirshfeld
Surface Analysis. CrystEngComm.

[47] Aydin F., Arslan N. B. (2022). Synthesis, Crystal
Structure and Cyclic Voltammetric
Behavior of N–Aroyl–N′–(4′–Cyanophenyl)­Thioureas. Molbank.

[48] Singh D. P., Pratap S., Gupta S. K., Butcher R. J. (2013). Structural and Spectral
Speciation on Methyl 2-(3-(Furan-2-Carbonyl)­Thioureido)­Benzoate: A
Comparative Experimental and Theoretical Study. J. Mol. Struct..

[49] Hamad A. R., Ahmed K. M., Omer R. A., Azeez Y. H., Kareem R. O., Othman K. A., Amin A. A. (2025). Synthesis,
Characterization and Computational
Study of Thiourea-Based Dihydropyrimidine Derivatives: A Focus on
Adsorption and Reactivity. J. Mol. Struct..

[50] Kosar B., Albayrak C. (2011). Spectroscopic Investigations
and Quantum Chemical Computational
Study of (E)–4–Methoxy–2–[(p–Tolylimino)­Methyl]­Phenol. Spectrochim. Acta, Part A.

[51] Spackman M. A., McKinnon J. J., Jayatilaka D. (2008). Electrostatic
Potentials Mapped on
Hirshfeld Surfaces Provide Direct Insight into Intermolecular Interactions
in Crystals. CrystEngComm.

[52] Dennington, R. ; Keith, T. A. ; Millam, J. M. GaussView, Version 6; Semichem Inc.: Shawnee Mission, KS, USA, 2016.

[53] Daina A., Michielin O., Zoete V. (2017). SwissADME: A Free Web
Tool to Evaluate
Pharmacokinetics, Drug–Likeness and Medicinal Chemistry Friendliness
of Small Molecules. Sci. Rep..

[54] Pires D. E. V., Blundell T. L., Ascher D. B. (2015). PkCSM:
Predicting Small–Molecule
Pharmacokinetic and Toxicity Properties Using Graph–Based Signatures. J. Med. Chem..

[55] Lipinski C. A., Lombardo F., Dominy B. W., Feeney P. J. (1997). Experimental and
Computational Approaches to Estimate Solubility and Permeability in
Drug Discovery and Development Settings. Adv.
Drug Delivery Rev..

[56] Veber D. F., Johnson S. R., Cheng H.–Y., Smith B. R., Ward K. W., Kopple K. D. (2002). Molecular Properties
That Influence the Oral Bioavailability
of Drug Candidates. J. Med. Chem..

[57] Egan W. J., Merz K. M., Baldwin J. J. (2000). Prediction of Drug
Absorption Using Multivariate Statistics. J.
Med. Chem..

[58] Ghose A. K., Viswanadhan V. N., Wendoloski J. J. (1999). A Knowledge–Based Approach
in Designing Combinatorial or Medicinal Chemistry Libraries for Drug
Discovery. 1. A Qualitative and Quantitative Characterization of Known
Drug Databases. J. Comb. Chem..

[59] Muegge I., Heald S. L., Brittelli D. (2001). Simple Selection
Criteria for Drug–like
Chemical Matter. J. Med. Chem..

[60] Banerjee P., Eckert A. O., Schrey A. K., Preissner R. (2018). ProTox–II:
A Webserver for the Prediction of Toxicity of Chemicals. Nucleic Acids Res..

[61] Toprakçí M., Yelekçi K. (2005). Docking Studies
on Monoamine Oxidase–B Inhibitors:
Estimation of Inhibition Constants (K­(i)) of a Series of Experimentally
Tested Compounds. Bioorg. Med. Chem. Lett..

[62] Binda C., Newton–Vinson P., Hubálek F., Edmondson D. E., Mattevi A. (2002). Structure of Human Monoamine Oxidase B, a Drug Target
for the Treatment of Neurological Disorders. Nat. Struct. Biol..

[63] Wojciechowska A., Bregier-Jarzębowska R., Komarnicka U. K., Szuster-Ciesielska A., Sułek M., Bojarska-Junak A., Ramadan R. M., Jezierska J. (2025). Solution Structure,
Oxidative DNA
Damage, Biological Activity and Molecular Docking of Ternary Copper­(II)
L–Argininato Complexes. Biochimie.

[64] Morris G. M., Huey R., Lindstrom W., Sanner M. F., Belew R. K., Goodsell D. S., Olson A. J. (2009). AutoDock4
and AutoDockTools4: Automated
Docking with Selective Receptor Flexibility. J. Comput. Chem..

[65] Lakey J. H., Craig P. A., Murray–Rust J. (2002). Theory and
Simulation Macromolecular
Assemblages. Curr. Opin. Struct. Biol..

[66] Albobaledi Z., Tarahhomi A., Khaleghian A., van der Lee A., Excoffier G. (2024). Structural
Investigation, DNA/Mpox/COVID–19
Molecular Docking, and Biological Assays of Two Novel Co­(II) and Cu­(II)
Complexes Derived from Tri– and Bi–Dentate Pyridyl–Functionalized
Phosphoramide Ligand [Ph]­P­(O)­[NH–2Py]­2. Inorg. Chem. Commun..

[67] Pacureanu L., Bora A., Crisan L. (2023). New Insights on the
Activity and
Selectivity of MAO–B Inhibitors through in Silico Methods. Int. J. Mol. Sci..

[68] BIOVIA, Dassault Systèmes . Discovery Studio Visualizer; Dassault Systèmes: San Diego, 2016.

[69] Ariffin A., Rahman N. A., Yehye W. A., Alhadi A. A., Kadir F. A. (2014). PASS–Assisted
Design, Synthesis and Antioxidant Evaluation of New Butylated Hydroxytoluene
Derivatives. Eur. J. Med. Chem..

[70] Rana M. S., Rayhan N. M. A., Emon M. S. H., Islam M. T., Rathry K., Hasan M. M., Islam
Mansur M. M., Srijon B. C., Islam M. S., Ray A., Rakib M. A., Islam A., Kudrat–E–Zahan M., Hossen M. F., Asraf M. A. (2024). Antioxidant Activity of Schiff Base
Ligands Using the DPPH Scavenging Assay: An Updated Review. RSC Adv..

[71] Bughi D. H. C., Santos Y. L., Paveglio G. C., Silva S. M., Costa L. A. S., Iglesias B. A., Casagrande G. A., Pizzuti L. (2026). Synthesis, Photophysics,
and DPPH Assays of Novel Luminescent N–Thiocarbamoyl Pyrenyl
Pyrazolines. J. Photochem. Photobiol. A Chem..

[72] Huong D. Q., Van Bay M., Nam P. C. (2021). Antioxidant
Activity of Thiourea
Derivatives: An Experimental and Theoretical Study. J. Mol. Liq..

[73] Abosadiya H. M. (2020). Synthesis,
Crystal Structure and Antioxidant Evaluation of N–(4–Formylpiperazine–1–Carbonothioyl)­Benzamide. Eur. J. Chem..

[74] Alizada A., Arslan H. (2023). Experimental and Theoretical
Studies of a Thiourea
Derivative: 1–(4–Chloro–Benzoyl)–3–(2–Trifluoromethyl–Phenyl)­Thiourea. J. Mol. Struct..

[75] Gribble G. W., Bousquet F. P. (1971). Deshielding Effects
in the NMR Spectra of Ortho–Substituted
Anilides and Thioanilides. Tetrahedron.

[76] Binzet G., Kavak G., Külcü N., Özbey S., Flörke U., Arslan H. (2013). Synthesis and Characterization of
Novel Thiourea Derivatives and Their Nickel and Copper Complexes. J. Chem..

[77] Ozer C. K., Solmaz U., Arslan H. (2021). Crystal Structure, Hirshfeld Surface
Analysis, and DFT Studies of N–(2–Chlorophenylcarbamothioyl)­Cyclohexanecarboxamide. Eur. J. Chem..

[78] Atiş M., Karipcin F., Sarıboğa B., Taş M., Çelik H. (2012). Structural, Antimicrobial and Computational
Characterization
of 1–Benzoyl–3–(5–Chloro–2–Hydroxyphenyl)­Thiourea. Spectrochim. Acta, Part A.

[79] Steinebach C., Ambrożak A., Dosa S., Beedie S. L., Strope J. D., Schnakenburg G., Figg W. D., Gütschow M. (2018). Synthesis,
Structural Characterization, and Antiangiogenic Activity of Polyfluorinated
Benzamides. ChemMedChem.

[80] Qu H., Liang P., Shi Q., Xiao S., Huang P., Wang H., Zhou Z., Liu L. (2025). Hypervalent Iodine-Mediated
Highly Mono- and Di-Selective Etherification of 8-Aminoquinolines
at the C5 and C6 Positions. Org. Biomol. Chem..

[81] Saunders C., Khaled M. B., Weaver J. D., Tantillo D. J. (2018). Prediction
of 19F NMR Chemical Shifts for Fluorinated Aromatic Compounds. J. Org. Chem..

[82] Limban C., Chifiriuc M. C., Caproiu M. T., Dumitrascu F., Ferbinteanu M., Pintilie L., Stefaniu A., Vlad I. M., Bleotu C., Marutescu L. G., Nuta D. C. (2020). New Substituted
Benzoylthiourea Derivatives: From Design to Antimicrobial Applications. Molecules.

[83] Abosadiya H. M. A. (2024). Synthesis,
Characterization, Crystal Structure and Antioxidant Activity of N–(3–Chloropropionyl)–N’–(4–Methoxyphenyl)­Thiourea. Eur. J. Chem..

[84] Abosadiya H. M., Anouar E. H., Hasbullah S. A., Yamin B. M. (2015). Synthesis, X–Ray,
NMR, FT–IR, UV/Vis, DFT and TD–DFT Studies of N–(4–Chlorobutanoyl)–N’–(2–,
3– and 4–Methylphenyl)­Thiourea Derivatives. Spectrochim. Acta, Part A.

[85] Saeed A., Ejaz S. A., Khalid A., Channar P. A., Aziz M., Wani T. A., Zargar S., Hassan S., Ismail H., Khalid D., Hashmi M. Z., Hökelek T., Aborode A. T. (2022). Facile Synthesis, Crystal Structure,
Biological Evaluation,
and Molecular Modeling Studies of N–((4–Acetyl Phenyl)
Carbamothioyl) Pivalamide as the Multitarget–Directed Ligand. Front. Chem..

[86] Khairul W. M., Wahab F. F. A., Soh S. K. C., Shamsuddin M., Daud A. I. (2020). Palladium­(II)–Pivaloyl Thiourea Complexes: Synthesis,
Characterisation and Their Catalytic Activity in Mild Sonogashira
Cross–Coupling Reaction. Chem. Phys.
Lett..

[87] Ritchie R. K., Spedding H. (1970). A Spectroscopic Study
of Thiourea Derivatives—II. Spectrochim.
Acta, Part A.

[88] Uysal M. E., Solmaz U., Arslan H. (2024). Ruthenium­(III) Acyl
Thiourea Complex:
A Catalyst for Transfer Hydrogenation of Nitroarenes. Polyhedron.

[89] Allen F. H., Kennard O., Watson D. G., Brammer L., Orpen A. G., Taylor R. (1987). Tables of Bond Lengths Determined
by X–Ray and
Neutron Diffraction. Part 1. Bond Lengths in Organic Compounds. J. Chem. Soc., Perkin Trans. 2.

[90] Gumus I., Solmaz U., Binzet G., Keskin E., Arslan B., Arslan H. (2019). Supramolecular Self–Assembly of New Thiourea
Derivatives Directed by Intermolecular Hydrogen Bonds and Weak Interactions:
Crystal Structures and Hirshfeld Surface Analysis. Res. Chem. Intermed..

[91] Kadir M. A., Yamin B. M., Yusof M. S. M. (2012). N–(2,2–Dimethyl–Propano–Yl)–N’–(2–Meth–Oxy–Phen–Yl)­Thio–Urea. Acta Crystallogr., Sect. E:Struct. Rep. Online.

[92] Solmaz U., Arslan H. (2022). Spectral, Crystallographic,
Theoretical, and Catalytic
Activity Studies of the PdII Complexes in Different Coordination Modes
of Benzoylthiourea Ligand. J. Mol. Struct..

[93] Nygaard L., Bojesen I., Pedersen T., Rastrup–Andersen J. (1968). Structure
of Fluorobenzene. J. Mol. Struct..

[94] Uysal M. E., Solmaz U., Arslan H. (2023). Ru­(II) and
Ru­(III) Complexes Containing *N*-acylthiourea Ligands:
Supramolecular Structures and Synthons,
Reduction, and Reaction Pathway of Aromatic Nitro Compounds. Appl. Organomet. Chem..

[95] Keskin E., Solmaz U., Gumus I., Arslan H. (2022). Di– and Tetra–Nuclear
Oxorhenium­(V) Complexes of Benzoylthiourea Derivative Ligands: Synthesis,
Structural Characterization, and Catalytic Applications. Polyhedron.

[96] Ashfaq M., Tahir M. N., Muhammad S., Munawar K. S., Ali A., Bogdanov G., Alarfaji S. S. (2021). Single–Crystal Investigation,
Hirshfeld Surface Analysis, and DFT Study of Third–Order NLO
Properties of Unsymmetrical Acyl Thiourea Derivatives. ACS Omega.

[97] Sow S., Thiam M., Odame F., Thiam E. I., Diouf O., Ellena J., Gaye M., Tshentu Z. (2024). Crystal Structure of
1–(1,3–Benzo–Thia–Zol–2–Yl)–3–(4–Bromo–Benzo–Yl)­Thio–Urea. Acta Crystallogr. Sect. E Crystallogr. Commun..

[98] Fun H.–K., Quah C. K., Nayak P. S., Narayana B., Sarojini B. K. (2012). 1–(3–Chloro–Phen–Yl)­Thio–Urea. Acta Crystallogr. Sect. E Struct. Rep. Online.

[99] Desiraju, G. R. The Supramolecular Synthon in Crystal Engineering. In Stimulating Concepts in Chemistry; Wiley–VCH Verlag GmbH & Co. KGaA: Weinheim, FRG, 2005; pp 293–306 10.1002/3527605746.ch19.

[100] Benaissa H., Adarsh N. N., Robeyns K., Zakrzewski J. J., Chorazy S., Hooper J. G. M., Sagan F., Mitoraj M. P., Wolff M., Radi S., Garcia Y. (2021). Exploring “Triazole–Thiourea”
Based Ligands for the Self–Assembly of Photoluminescent Hg­(II)
Coordination Compounds. Cryst. Growth Des..

[101] Qiao L., Huang J., Hu W., Zhang Y., Guo J., Cao W., Miao K., Qin B., Song J. (2017). Synthesis,
Characterization, and in Vitro Evaluation and in Silico Molecular
Docking of Thiourea Derivatives Incorporating 4-(Trifluoromethyl)­Phenyl
Moiety. J. Mol. Struct..

[102] Eccles K. S., Morrison R. E., Maguire A. R., Lawrence S. E. (2014). Crystal
Landscape of Primary Aromatic Thioamides. Cryst.
Growth Des..

[103] Saeed A., Khurshid A., Bolte M., Fantoni A. C., Erben M. F. (2015). Intra-
and Intermolecular Hydrogen Bonding and Conformation
in 1-Acyl Thioureas: An Experimental and Theoretical Approach on 1-(2-Chlorobenzoyl)­Thiourea. Spectrochim. Acta, Part A.

[104] Al-Wahaibi L. H., Grandhi D. S., Tawfik S. S., Al-Shaalan N. H., Elmorsy M. A., El-Emam A. A., Percino M. J., Thamotharan S. (2021). Probing the
Effect of Halogen Substituents (Br, Cl, and F) on the Non-Covalent
Interactions in 1-(Adamantan-1-Yl)-3-Arylthiourea Derivatives: A Theoretical
Study. ACS Omega.

[105] Saeed A., Bolte M., Erben M. F., Pérez H. (2015). Intermolecular
Interactions in Crystalline 1-(Adamantane-1-Carbonyl)-3-Substituted
Thioureas with Hirshfeld Surface Analysis. CrystEngComm.

[106] Eccles K. S., Morrison R. E., Sinha A. S., Maguire A. R., Lawrence S. E. (2015). Investigating
C=S···I Halogen
Bonding for Cocrystallization with Primary Thioamides. Cryst. Growth Des..

[107] Shestimerova T. A., Medved’ko A. V., Bykov M. A., Kalinin M. A., Metlin M. T., Taydakov I. V., Gontcharenko V. E., Wei Z., Dikarev E. V., Vatsadze S. Z., Shevelkov A. V. (2025). A New Supramolecular
Tecton: The Crucial Impact of the Polycation Charge and Geometry of
H–Bonds on the Structure and Properties of Halometallates in
the Solid State. Dalton Trans..

[108] Bourne S. A., Hallale O., Koch K. R. (2005). Hydrogen–Bonding
Networks in a Bipodal Acyl–Thiourea and Its NiII 2:2 Metallamacrocyclic
Complex. Cryst. Growth Des..

[109] Gimeno M. C., Herrera R. P. (2016). Hydrogen Bonding
Networks in Chiral
Thiourea Organocatalysts: Evidence on the Importance of the Aminoindanol
Moiety. Cryst. Growth Des..

[110] Paisner K., Zakharov L. N., Doxsee K. M. (2010). A Robust
Thiourea
Synthon for Crystal Engineering. Cryst. Growth
Des..

[111] Sun H., Mei H., Xie C., Lu Z., Han J., Pan Y. (2010). Modified Thiourea
Used as the Ligand for the Synthesis of Novel Coordination
Polymers: Based on Its Significant Conformational Behavior and H–Bonding
Preference. Inorg. Chem. Commun..

[112] Reddy D. S., Ovchinnikov Y. E., Shishkin O. V., Struchkov Y. T., Desiraju G. R. (1996). Supramolecular Synthons in Crystal Engineering. 3.
Solid State Architecture and Synthon Robustness in Some 2,3–Dicyano–5,6–Dichloro–1,4–Dialkoxybenzenes1. J. Am. Chem. Soc..

[113] Perpétuo G. J., Janczak J. (2013). Supramolecular Hydrogen–Bonding
Networks in the 1–(Diaminomethylenene)­Thiouron–1–Ium
4–Hydroxybenzoate, 3,4–Dihydroxybenzoate and 3,4,5–Trihydroxybenzoate
Monohydrate Crystals. J. Mol. Struct..

[114] Pramanik A., Das G. (2011). Interplay of Solvent
in Flexible
Behaviour of Cyclohexane Dinapthyl Bis–Thiourea System: Conformational
Aspects. Supramol. Chem..

[115] Day G. M., Trask A. V., Motherwell W. D. S., Jones W. (2006). Investigating the Latent Polymorphism of Maleic Acid. Chem. Commun..

[116] Gumus I., Solmaz U., Binzet G., Keskin E., Arslan B., Arslan H. (2018). Hirshfeld Surface Analyses
and Crystal
Structures of Supramolecular Self–Assembly Thiourea Derivatives
Directed by Non–Covalent Interactions. J. Mol. Struct..

[117] McKinnon J. J., Spackman M. A., Mitchell A. S. (2004). Novel Tools
for
Visualizing and Exploring Intermolecular Interactions in Molecular
Crystals. Acta Crystallogr., Sect. B:Struct.
Sci..

[118] Qiao L., Zhang Y., Hu W., Guo J., Cao W., Ding Z., Guo Z., Fan A., Song J., Huang J. (2017). Synthesis, Structural Characterization
and Quantum Chemical Calculations
on 1-(Isomeric Methylbenzoyl)-3-(4-Trifluoromethylphenyl)­Thioureas. J. Mol. Struct..

[119] Leclerc N., Chávez P., Ibraikulov O. A., Heiser T., Lévêque P. (2016). Impact of
Backbone
Fluorination on π–Conjugated Polymers in Organic Photovoltaic
Devices: A Review. Polymers.

[120] Fuhrer T. J., Houck M., Iacono S. T. (2021). Fluoromaticity:
The Molecular Orbital Contributions of Fluorine Substituents to the
π–Systems of Aromatic Rings. ACS
Omega.

[121] Morad R., Akbari M., Maaza M. (2023). Theoretical Study of
Chemical Reactivity Descriptors of Some Repurposed Drugs for COVID–19. MRS Adv..

[122] Oladipo S. D., Luckay R. C., Olalekan S. O., Badeji A. A., Matinise N., Tshikhudo F. (2025). Investigating
the Inhibitory Potential
of Halogenated Quinoline Derivatives against MAO-A and MAO–B:
Synthesis, Crystal Structure, Density Functional Theory, and Molecular
Dynamics Simulations. ACS Omega.

[123] Mohan N., Suresh C. H. (2014). A Molecular Electrostatic Potential
Analysis of Hydrogen, Halogen, and Dihydrogen Bonds. J. Phys. Chem. A.

[124] Lipinski C. A., Lombardo F., Dominy B. W., Feeney P. J. (2001). Experimental
and Computational Approaches to Estimate Solubility and Permeability
in Drug Discovery and Development Settings. Adv. Drug Delivery Rev..

[125] Giacomini K. M., Huang S.–M., Tweedie D. J., Benet L. Z., Brouwer K. L. R., Chu X., Dahlin A., Evers R., Fischer V., Hillgren K. M., Hoffmaster K. A., Ishikawa T., Keppler D., Kim R. B., Lee C. A., Niemi M., Polli J. W., Sugiyama Y., Swaan P. W., Ware J. A., Wright S. H., Yee S. W., Zamek–Gliszczynski M. J., Zhang L. (2010). Membrane Transporters
in Drug Development. Nat. Rev. Drug Discovery.

[126] Valerio Jr L. G. (2009). In Silico Toxicology
for the Pharmaceutical
Sciences. Toxicol. Appl. Pharmacol..

[127] Raies A. B., Bajic V. B. (2016). In Silico Toxicology: Computational
Methods for the Prediction of Chemical Toxicity: Computational Methods
for the Prediction of Chemical Toxicity. WIREs
Comput. Mol. Sci..

[128] Morris G. M., Goodsell D. S., Halliday R. S., Huey R., Hart W. E., Belew R. K., Olson A. J. (1998). Automated Docking
Using a Lamarckian Genetic Algorithm and an Empirical Binding Free
Energy Function. J. Comput. Chem..

[129] Kitchen D. B., Decornez H., Furr J. R., Bajorath J. (2004). Docking and
Scoring in Virtual Screening for Drug Discovery: Methods and Applications. Nat. Rev. Drug Discovery.

[130] Sadybekov A. V., Katritch V. (2023). Computational Approaches
Streamlining
Drug Discovery. Nature.

[131] Wilcken R., Zimmermann M. O., Lange A., Joerger A. C., Boeckler F. M. (2013). Principles and Applications
of Halogen Bonding in Medicinal
Chemistry and Chemical Biology. J. Med. Chem..

[132] de Menezes B. B., Frescura L. M., Duarte R., Villetti M. A., da Rosa M. B. (2021). A Critical Examination
of the DPPH Method: Mistakes
and Inconsistencies in Stoichiometry and IC50 Determination by UV–Vis
Spectroscopy. Anal. Chim. Acta.

[133] Shalaby M. A., Fahim A. M., Rizk S. A. (2023). Antioxidant
Activity
of Novel Nitrogen Scaffold with Docking Investigation and Correlation
of DFT Stimulation. RSC Adv..

[134] Costea T. (2020). Antioxidant Activity and Drug Profile
of Several Thiourea
Derivatives of 2–Thiophene Carboxylic Acid. Farmacia.

[135] Gillis E. P., Eastman K. J., Hill M. D., Donnelly D. J., Meanwell N. A. (2015). Applications of Fluorine in Medicinal
Chemistry. J. Med. Chem..

[136] Todorov L., Saso L., Kostova I. (2023). Antioxidant
Activity
of Coumarins and Their Metal Complexes. Pharmaceuticals.

[137] Pietruś W., Kurczab R., Warszycki D., Bojarski A. J., Bajorath J. (2023). Isomeric Activity
Cliffs–A
Case Study for Fluorine Substitution of Aminergic G Protein–Coupled
Receptor Ligands. Molecules.

[138] Ur Rahman F., Shah A. B., Muhammad M., Khan E., Ataya F. S., Batiha G. E.–S. (2024). Antioxidant,
Antibacterial, Enzyme
Inhibition and Fluorescence Characteristics of Unsymmetrical Thiourea
Derivatives. Heliyon.

[139] Pinheiro A. C., Wille A. P. B., Nunes I. J., Ferreira W. V., da Silva A. F., Kapavarapu R. K., Dias R. F. C., Cairo R. R., Stevens A. M. P., Saffi J., da Rocha V. M. E., Wilhelm E. A. (2026). Spectroscopic
and Theoretical Elucidation of Acylthiourea Derivatives with Antioxidant
and Low–Toxicity Profiles. J. Mol. Struct..

