# Supplementary material for: Integrated Experimental and Computational Investigation of Fluorinated Benzoylthiourea Derivatives: Structural Characterization, Hirshfeld Surface Analysis, Antioxidant Activity, and MAO–B Inhibitory Potential for Parkinson’s Disease
Source: ACS Omega. 2026 Sep 28;11(40):61549–73. doi: 10.1021/acsomega.6c07002 (PMC13670878; doi:10.1021/acsomega.6c07002)
Supplement: Supplementary file 1 [file ao6c07002_si_004.pdf]

SUPPORTING INFORMATION

Integrated experimental and computational investigation of fluorinated benzoylthiourea derivatives: Structural characterization, Hirshfeld surface analysis, antioxidant activity, and MAO–B inhibitory potential for Parkinson's disease

Lana Gul [a], Ummuhan Solmaz [b] and Hakan Arslan [a] *

[a] Mersin University, Faculty of Science, Department of Chemistry, Mersin, 33343, Türkiye
[b] Mersin University, Technical Science Vocational School, Department of Chemistry and Chemical Processing Technologies, Mersin, 33343, Türkiye
* Email: hakan.arslan@mersin.edu.tr

TABLE OF CONTENTS **Page**

Figure S1. ^{1}H NMR spectrum of *N*–(benzyl(methyl)carbamothioyl)–2–fluorobenzamide (FBTU1). 3
Figure S2. ^{1}H NMR spectrum of *N*–(benzyl(methyl)carbamothioyl)–3–fluorobenzamide (FBTU2). 3
Figure S3. ^{1}H NMR spectrum of *N*–(benzyl(methyl)carbamothioyl)–4–fluorobenzamide (FBTU3). 4
Figure S4. ^{13}C NMR spectrum of *N*–(benzyl(methyl)carbamothioyl)–2–fluorobenzamide (FBTU1). 4
Figure S5. ^{13}C NMR spectrum of *N*–(benzyl(methyl)carbamothioyl)–3–fluorobenzamide (FBTU2). 5
Figure S6. ^{13}C NMR spectrum of *N*–(benzyl(methyl)carbamothioyl)–4–fluorobenzamide (FBTU3). 5
Figure S7. ^{19}F NMR spectrum of *N*–(benzyl(methyl)carbamothioyl)–2–fluorobenzamide (FBTU1). 6
Figure S8. ^{19}F NMR spectrum of *N*–(benzyl(methyl)carbamothioyl)–3–fluorobenzamide (FBTU2). 6
Figure S9. ^{19}F NMR spectrum of *N*–(benzyl(methyl)carbamothioyl)–4–fluorobenzamide (FBTU3). 7
Figure S10. FT–IR spectrum of *N*–(benzyl(methyl)carbamothioyl)–2–fluorobenzamide (FBTU1). 8
Figure S11. FT–IR spectrum of *N*–(benzyl(methyl)carbamothioyl)–3–fluorobenzamide (FBTU2). 8
Figure S12. FT–IR spectrum of *N*–(benzyl(methyl)carbamothioyl)–4–fluorobenzamide (FBTU3). 8
Figure S13. UV–Vis spectrum of *N*–(benzyl(methyl)carbamothioyl)–2–fluorobenzamide (FBTU1). 9
Figure S14. UV–Vis spectrum of *N*–(benzyl(methyl)carbamothioyl)–3–fluorobenzamide (FBTU2). 9
Figure S15. UV–Vis spectrum of *N*–(benzyl(methyl)carbamothioyl)–4–fluorobenzamide (FBTU3). 9
Figure S16. Two–dimensional fingerprint plots and percentage contributions of intermolecular contacts for compound FBTU1. 10
Figure S17. Two–dimensional fingerprint plots and percentage contributions of intermolecular contacts for compound FBTU2. 11
Figure S18. Two–dimensional fingerprint plots and percentage contributions of intermolecular contacts for compound FBTU3. 12
Figure S19. Energy framework diagrams showing Coulombic (red), dispersion (green), and total (blue) interaction energies projected along the *a*–, *b*–, and *c*–axes for compound FBTU1. 13
Figure S20. Energy framework diagrams showing Coulombic (red), dispersion (green), and total (blue) interaction energies projected along the *a*–, *b*–, and *c*–axes for compound FBTU2. 14
Figure S21. Energy framework diagrams showing Coulombic (red), dispersion (green), and total (blue) interaction energies projected along the *a*–, *b*–, and *c*–axes for compound FBTU3. 15
Figure S22. Optimized geometries of compounds FBTU1 (a), FBTU2 (b), and FBTU3 (c) obtained from DFT calculations together with atom numbering. 16

TABLE OF CONTENTS	Page
**Figure S23.** Three–dimensional binding poses and two–dimensional interaction diagrams of ligands FBTU1 (a), FBTU2 (b), and FBTU3 (c) in the active site of the 1GOS protein.	17
**Table S1.** Bond lengths (Å) for FBTU1–FBTU3.	18
**Table S2.** Bond angles (°) for FBTU1–FBTU3.	18
**Table S3.** Color–coded visualization of intermolecular interacting molecular pairs together with their corresponding interaction energy components in the crystal structures of compound FBTU1.	19
**Table S4.** Color–coded visualization of intermolecular interacting molecular pairs together with their corresponding interaction energy components in the crystal structures of compound FBTU2.	19
**Table S5.** Color–coded visualization of intermolecular interacting molecular pairs together with their corresponding interaction energy components in the crystal structures of compound FBTU3.	19
**Table S6.** Mulliken atomic charge distributions of compounds FBTU1–FBTU3 obtained from DFT calculation.	20
**Table S7.** Molecular docking interactions of ligands FBTU1–FBTU3 within the active site of the 1GOS enzyme.	21

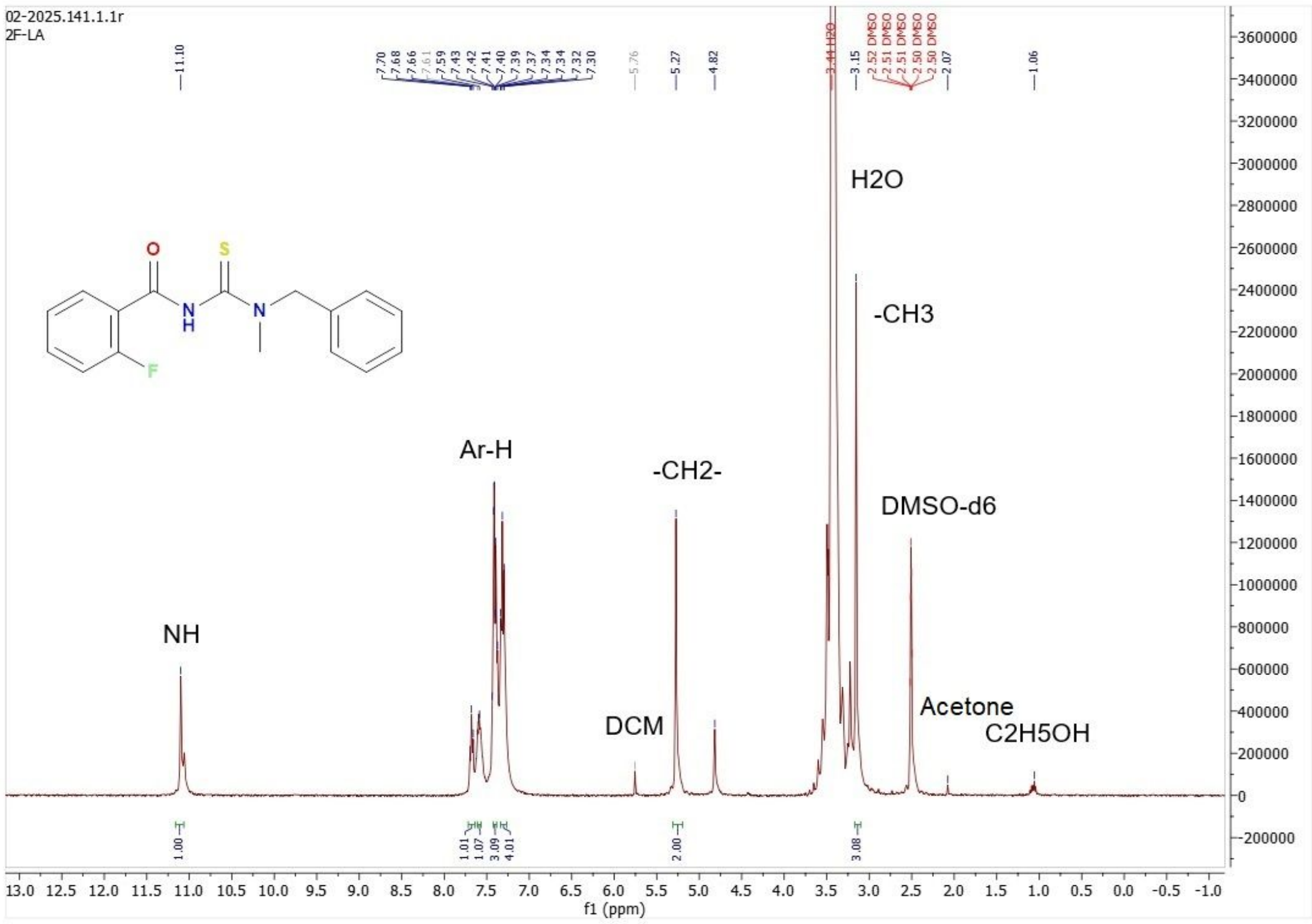

Figure S1. ^{1}H NMR spectrum of *N*–(benzyl(methyl)carbamothioyl)–2–fluorobenzamide (FBTU1).

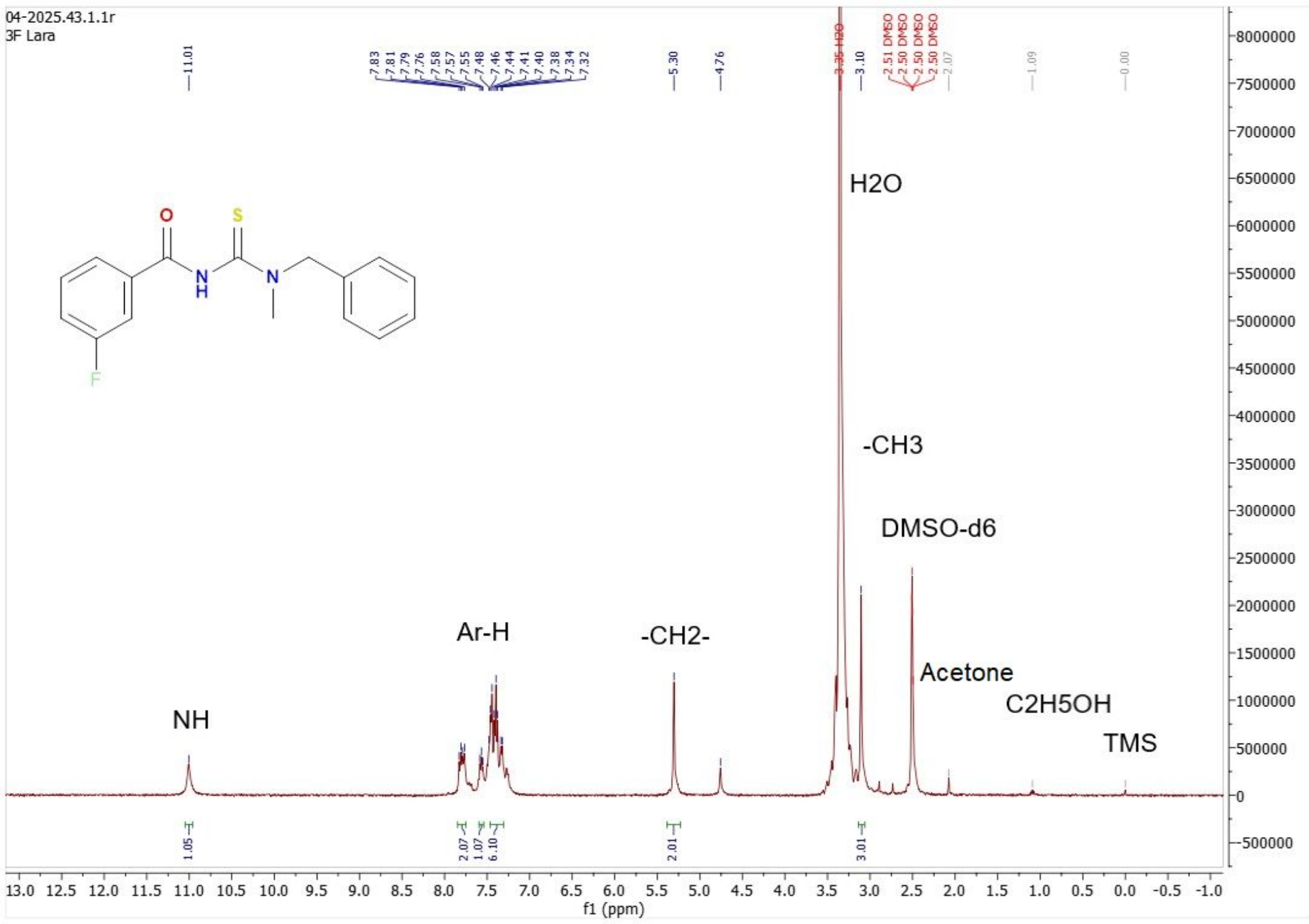

Figure S2. ^{1}H NMR spectrum of *N*–(benzyl(methyl)carbamothioyl)–3–fluorobenzamide (FBTU2).

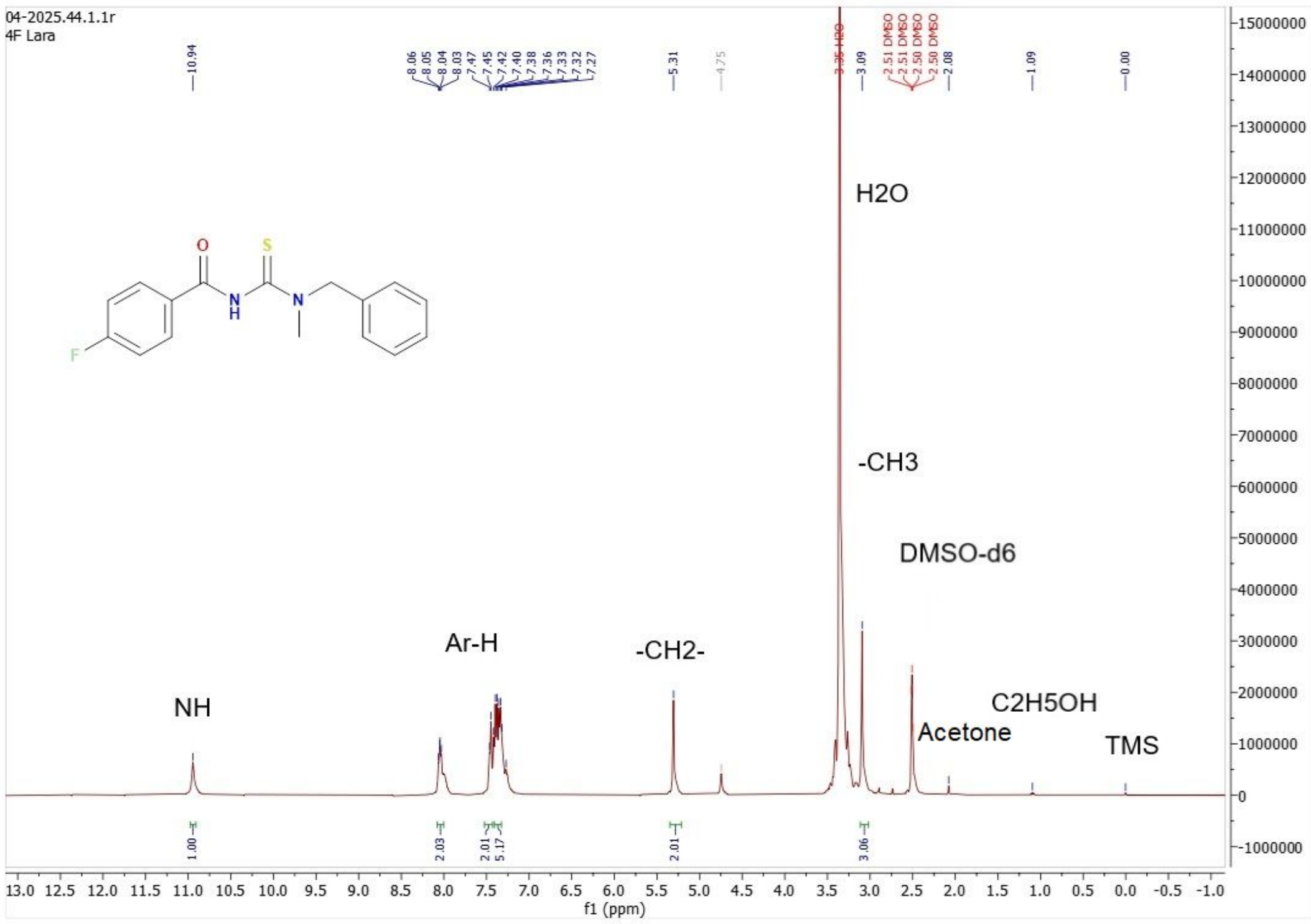

Figure S3. ^{1}H NMR spectrum of *N*–(benzyl(methyl)carbamothioyl)–4–fluorobenzamide (FBTU3).

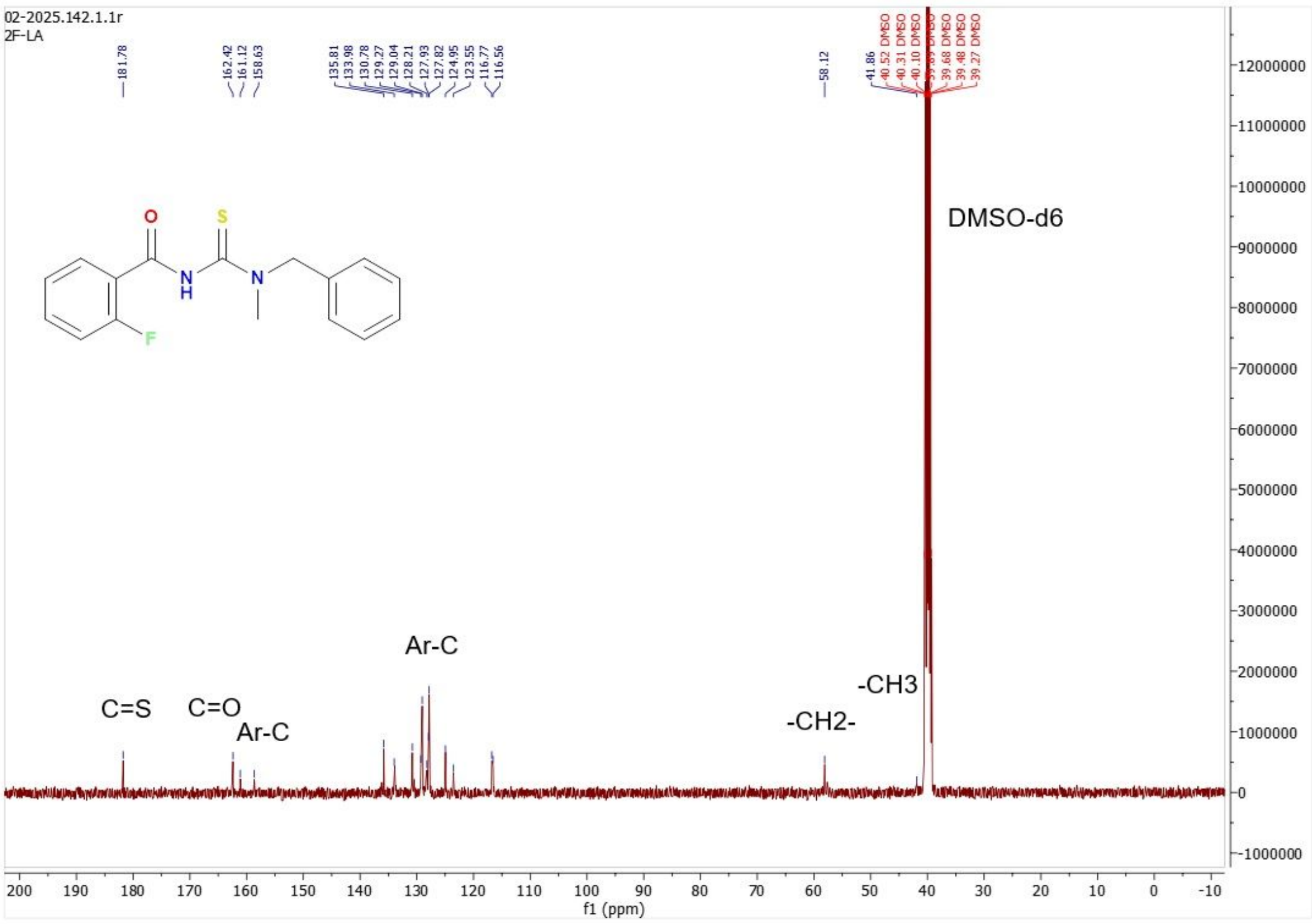

Figure S4. ^{13}C NMR spectrum of *N*–(benzyl(methyl)carbamothioyl)–2–fluorobenzamide (FBTU1).

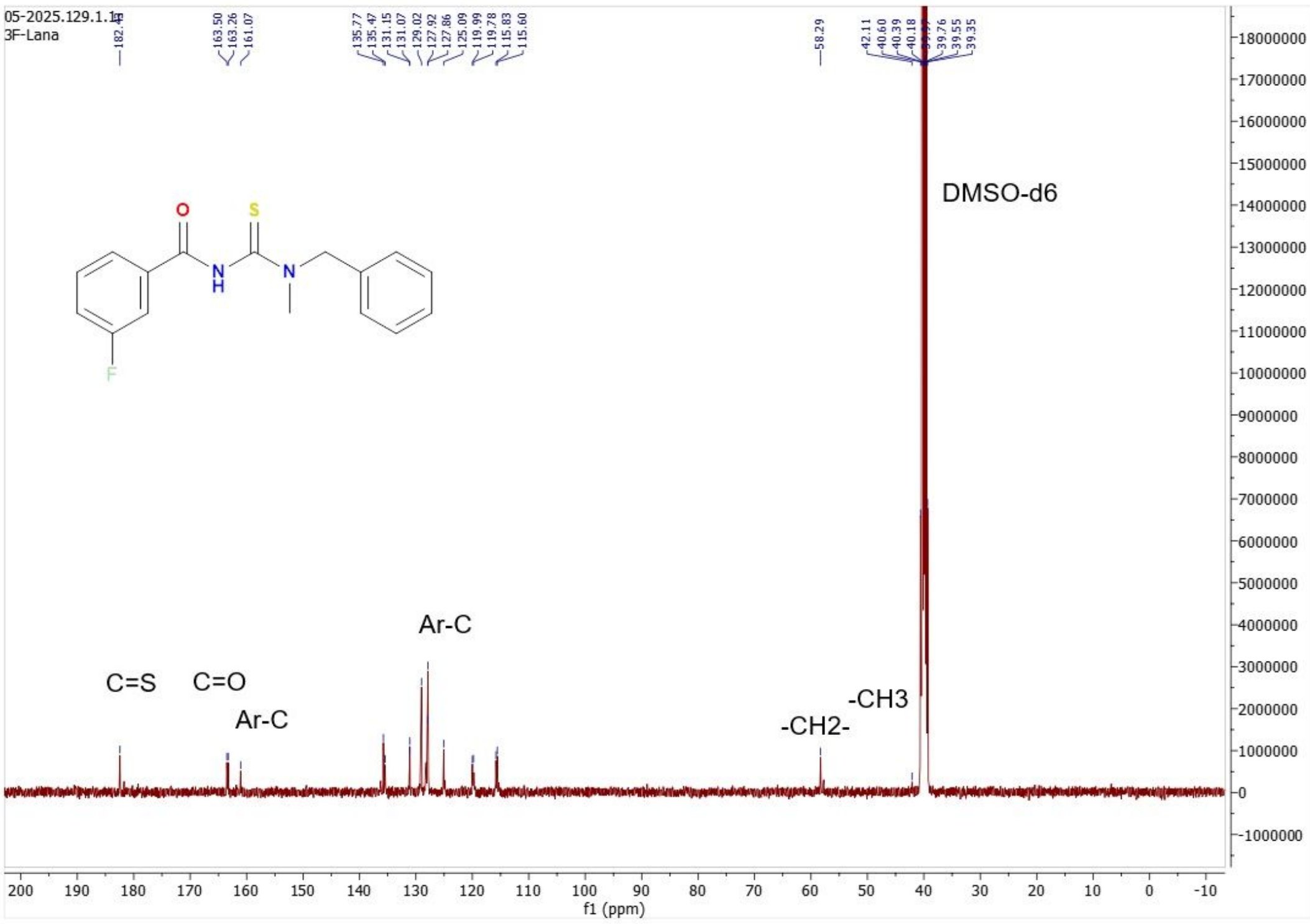

Figure S5. ^{13}C NMR spectrum of *N*–(benzyl(methyl)carbamothioyl)–3–fluorobenzamide (FBTU2).

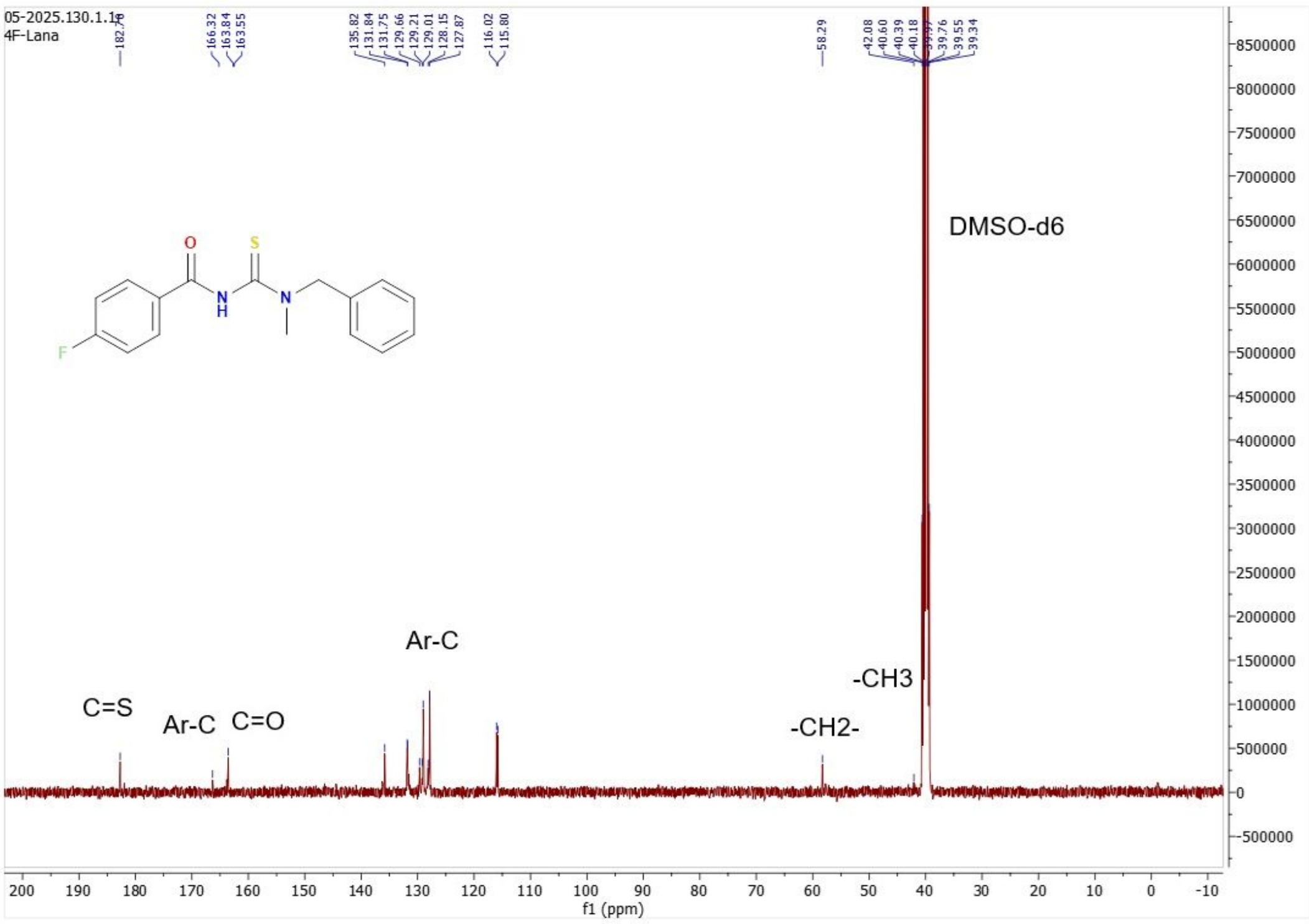

Figure S6. ^{13}C NMR spectrum of *N*–(benzyl(methyl)carbamothioyl)–4–fluorobenzamide (FBTU3).

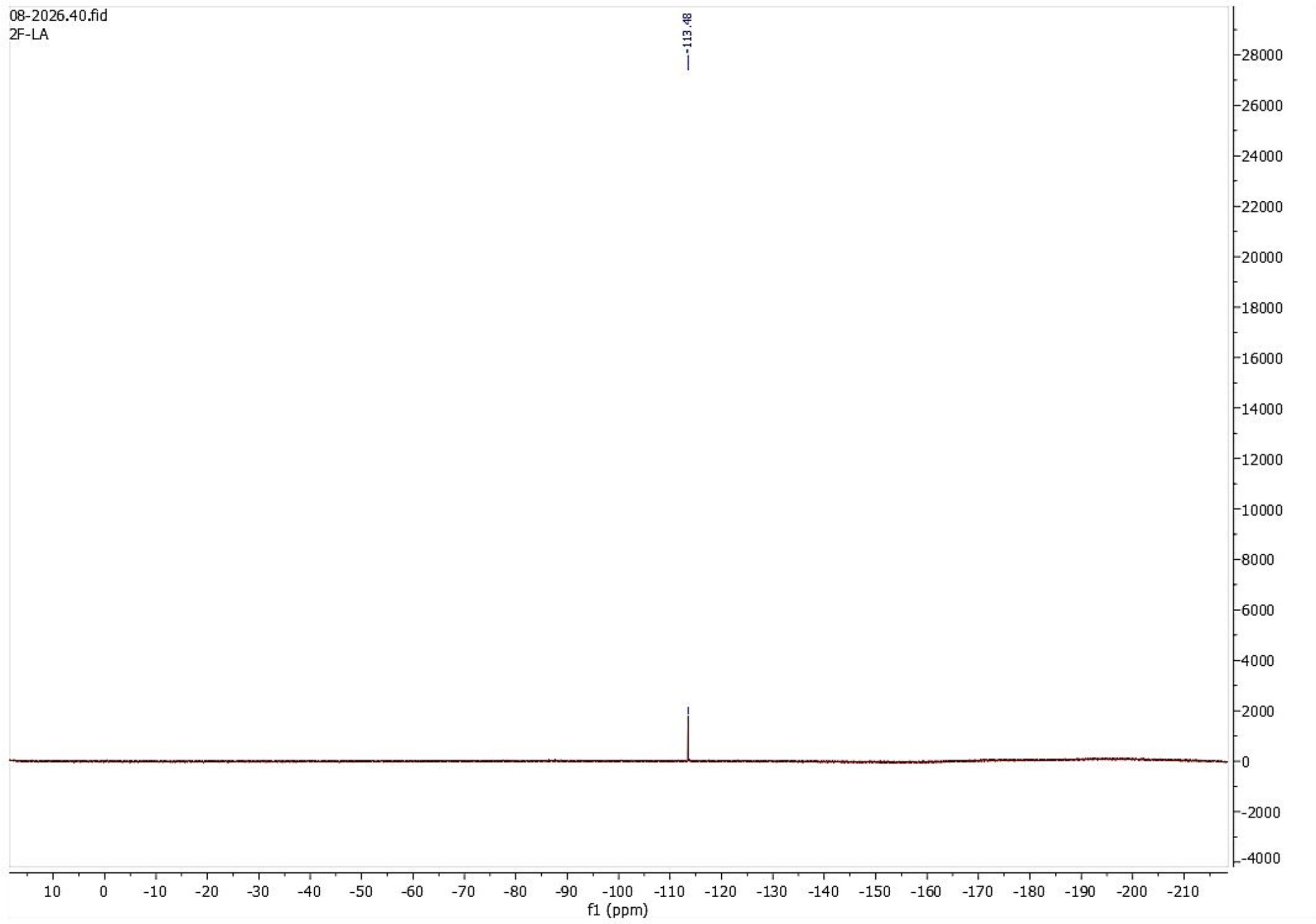

Figure S7. ^{19}F NMR spectrum of *N*–(benzyl(methyl)carbamothioyl)–2–fluorobenzamide (FBTU1).

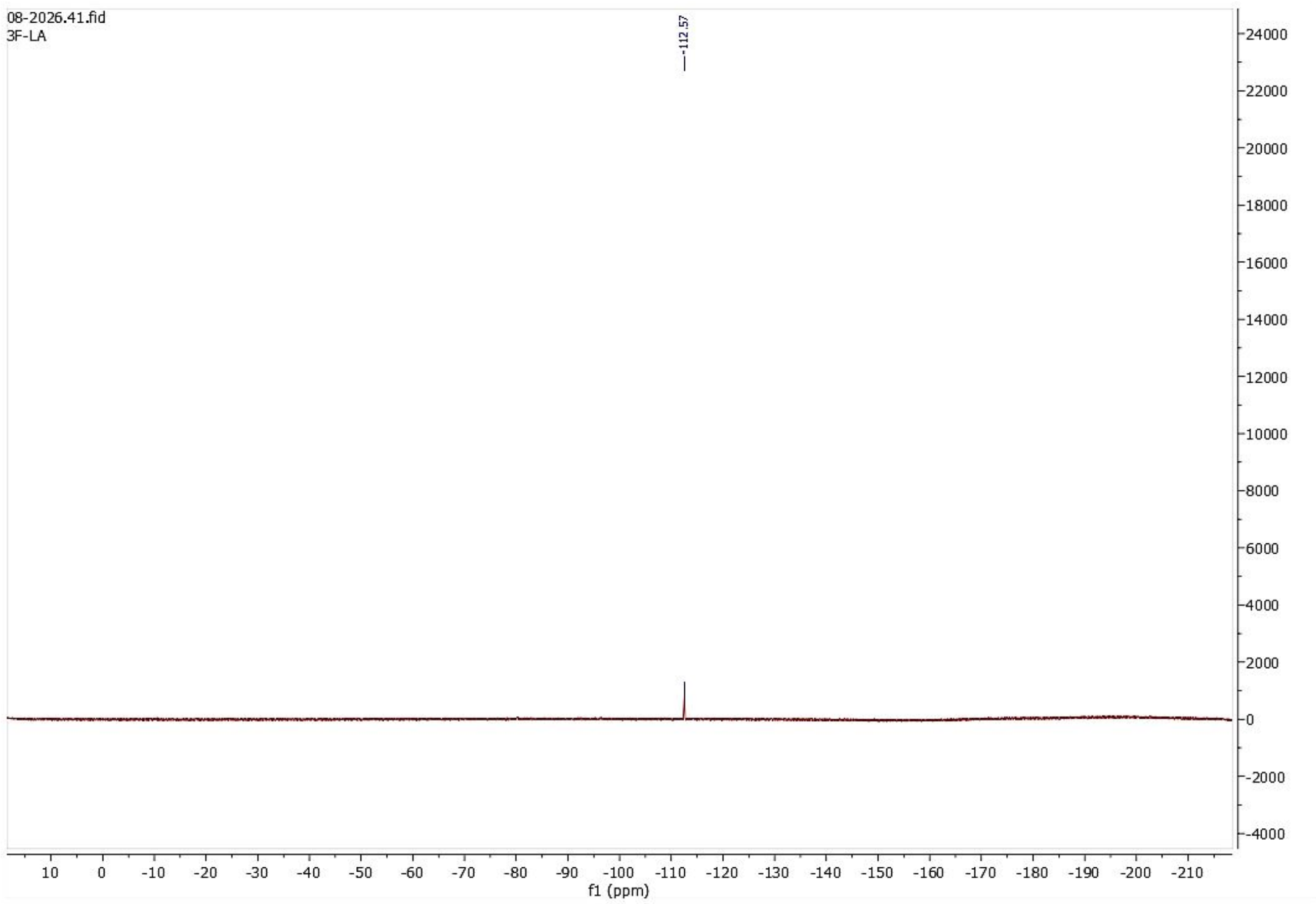

Figure S8. ^{19}F NMR spectrum of *N*–(benzyl(methyl)carbamothioyl)–3–fluorobenzamide (FBTU2).

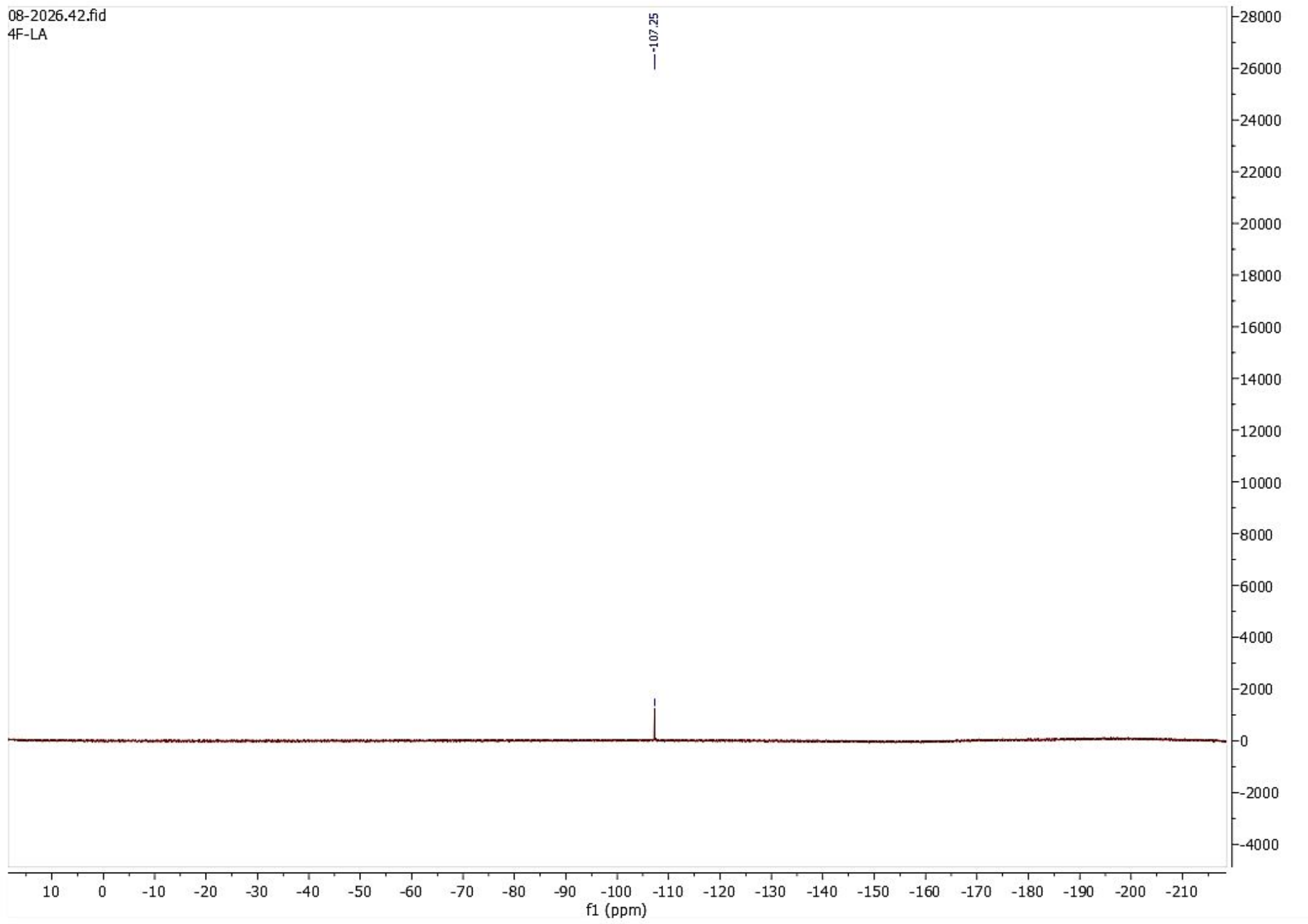

Figure S9. ^{19}F NMR spectrum of *N*–(benzyl(methyl)carbamothioyl)–4–fluorobenzamide (FBTU3).

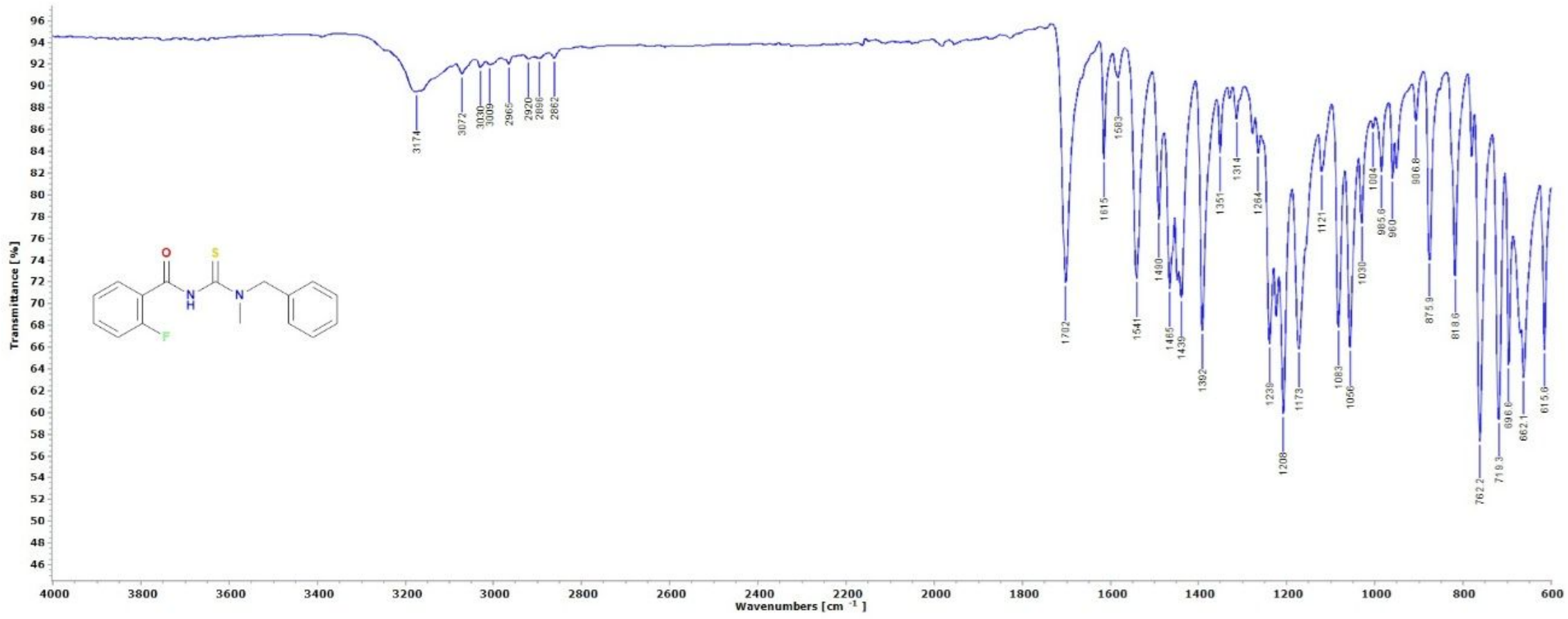

Figure S10. FT–IR spectrum of *N*–(benzyl(methyl)carbamothioyl)–2–fluorobenzamide (FBTU1).

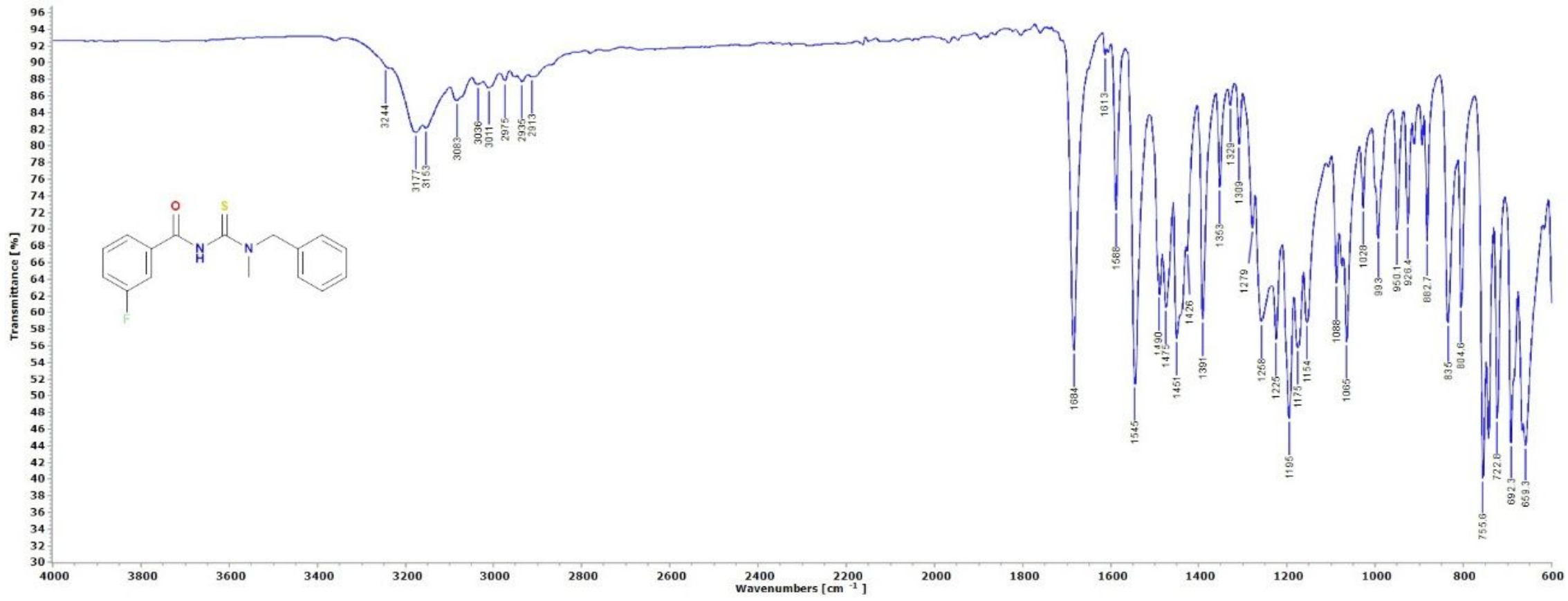

Figure S11. FT–IR spectrum of *N*–(benzyl(methyl)carbamothioyl)–3–fluorobenzamide (FBTU2).

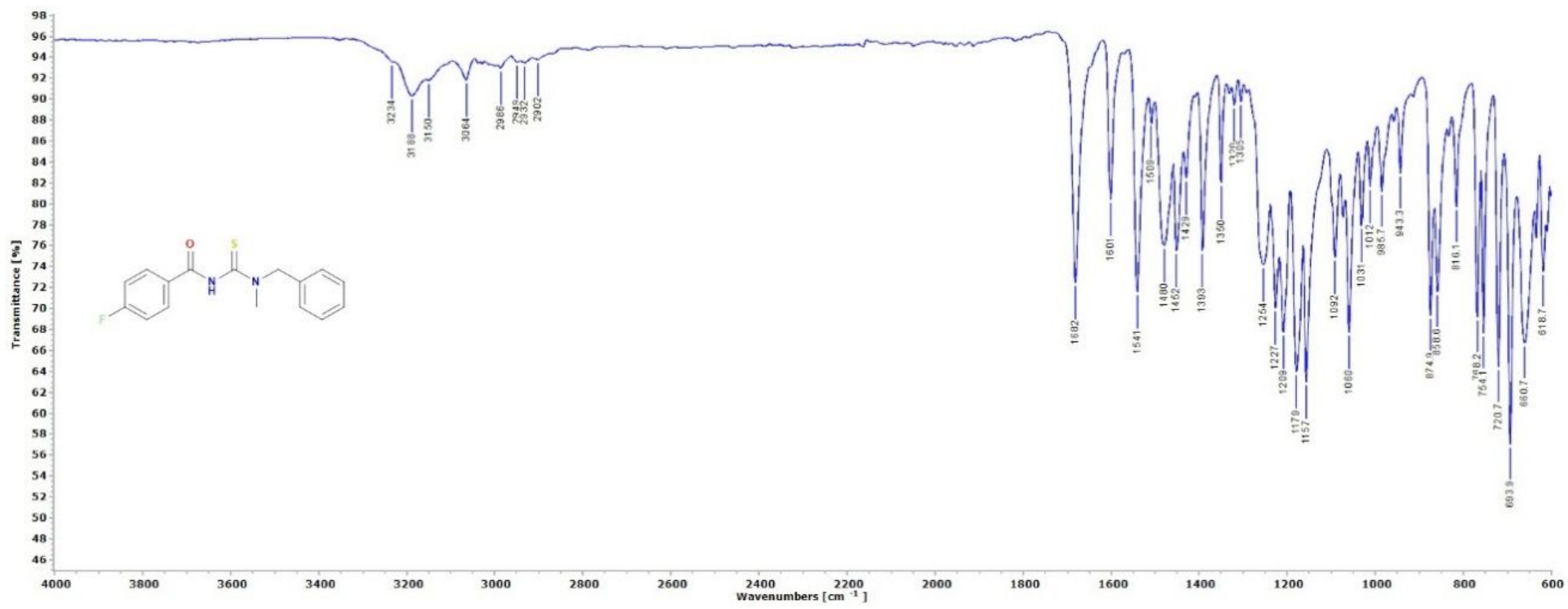

Figure S12. FT–IR spectrum of *N*–(benzyl(methyl)carbamothioyl)–4–fluorobenzamide (FBTU3).

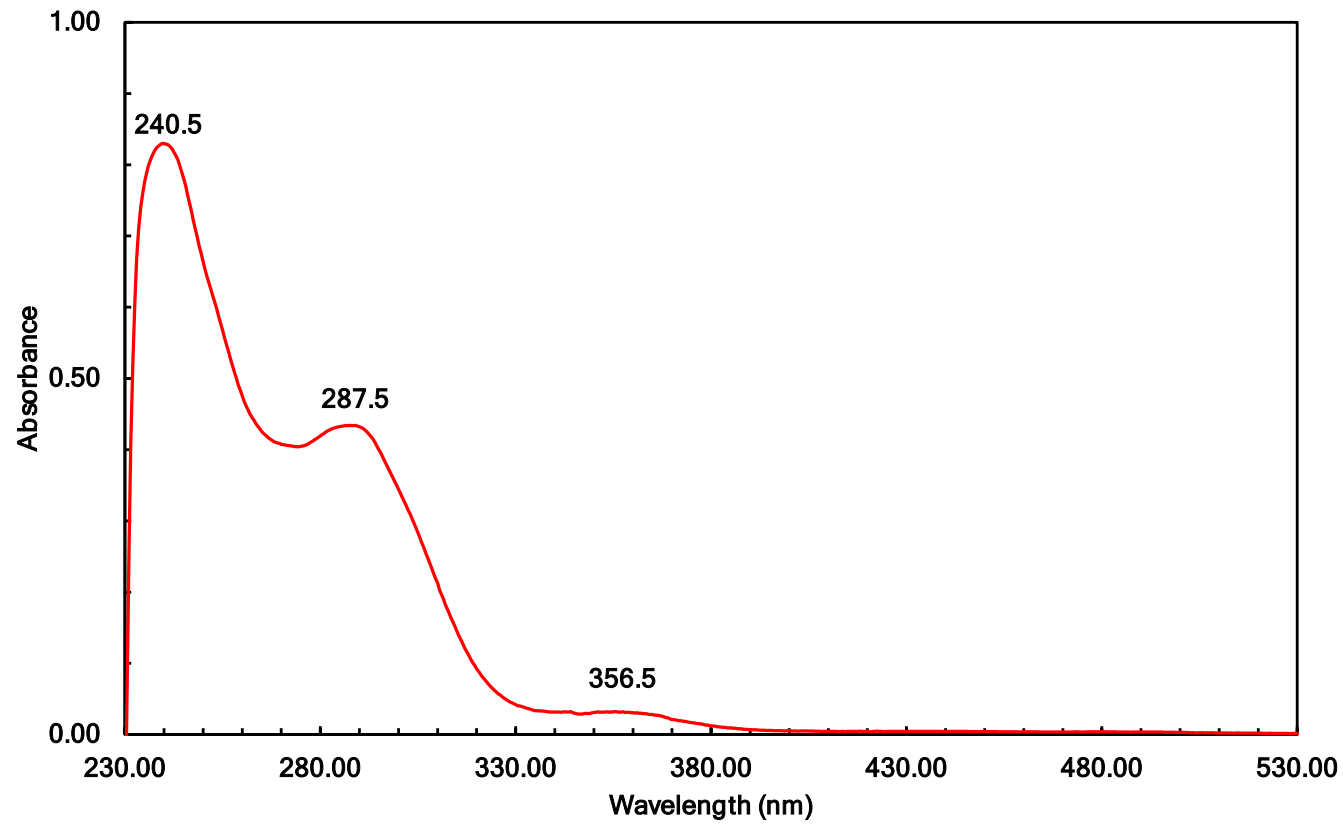

Figure S13. UV–Vis spectrum of *N*–(benzyl(methyl)carbamothioyl)–2–fluorobenzamide (FBTU1).

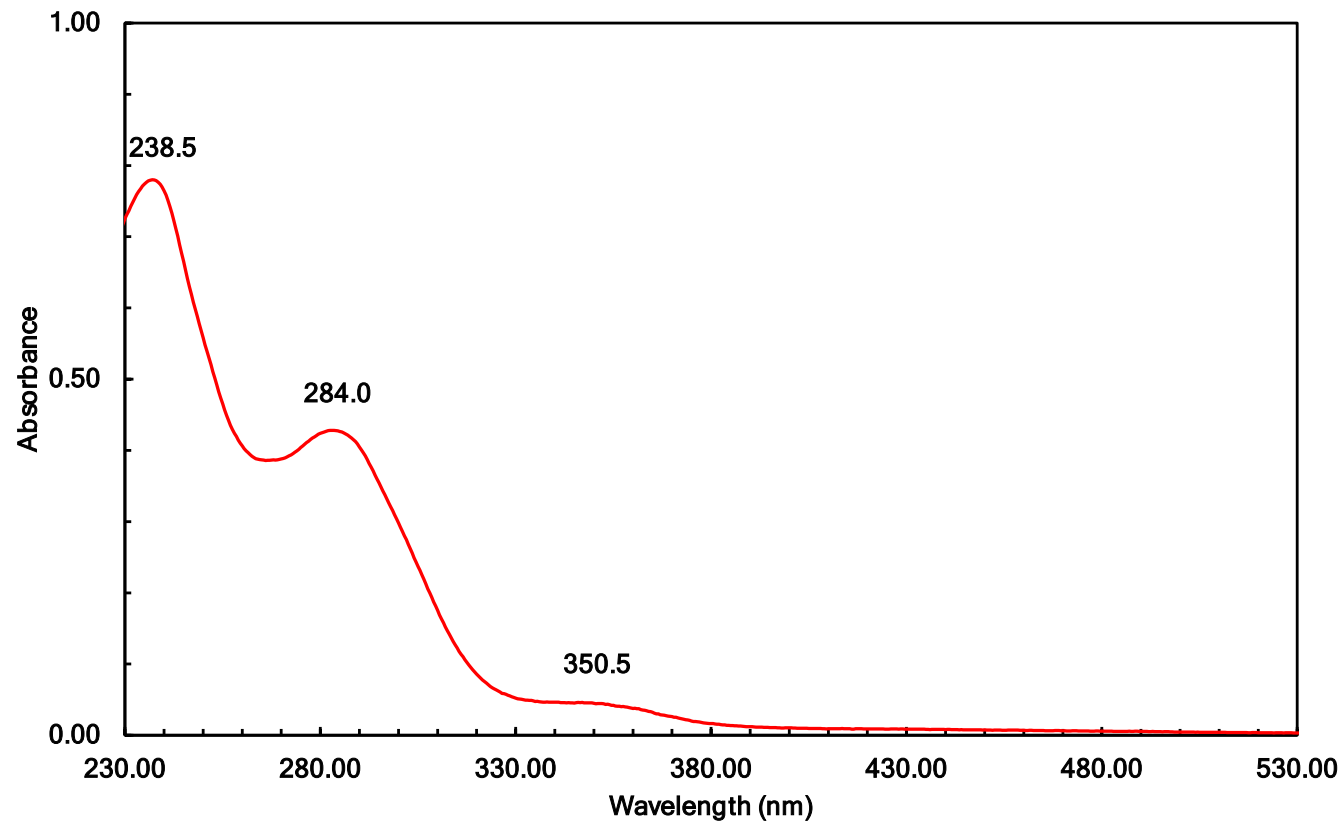

Figure S14. UV–Vis spectrum of *N*–(benzyl(methyl)carbamothioyl)–3–fluorobenzamide (FBTU2).

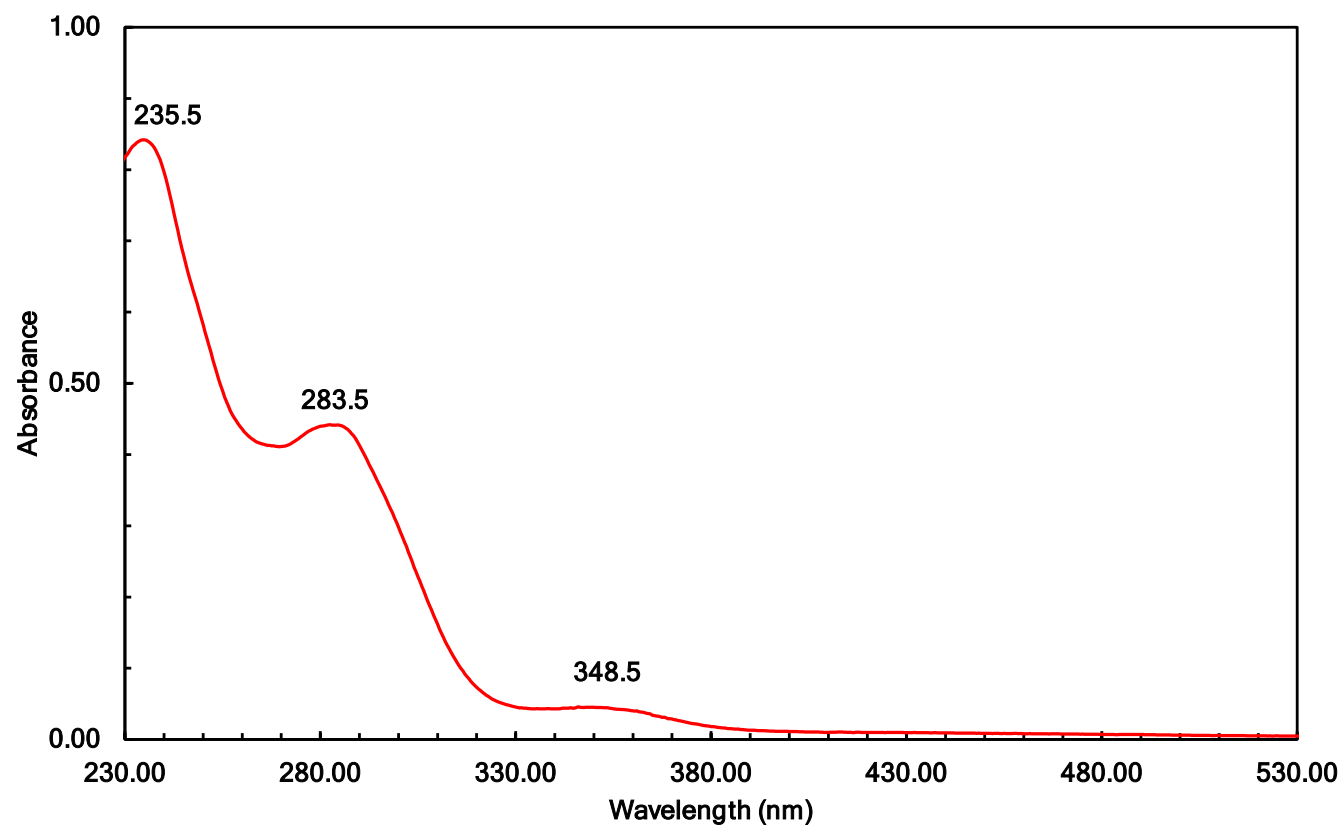

Figure S15. UV–Vis spectrum of *N*–(benzyl(methyl)carbamothioyl)–4–fluorobenzamide (FBTU3).

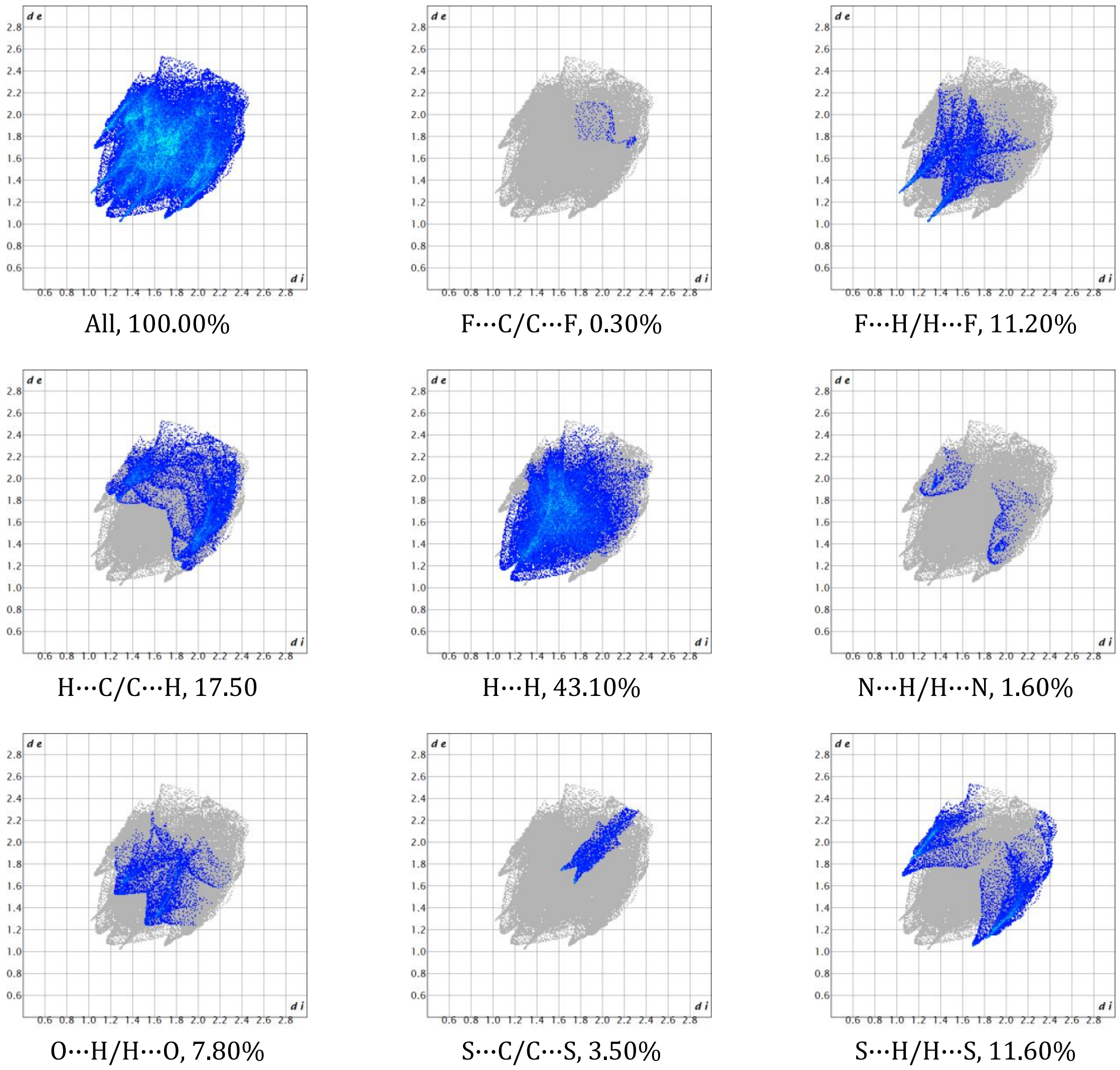

Figure S16. Two–dimensional fingerprint plots and percentage contributions of intermolecular contacts for compound FBTU1.

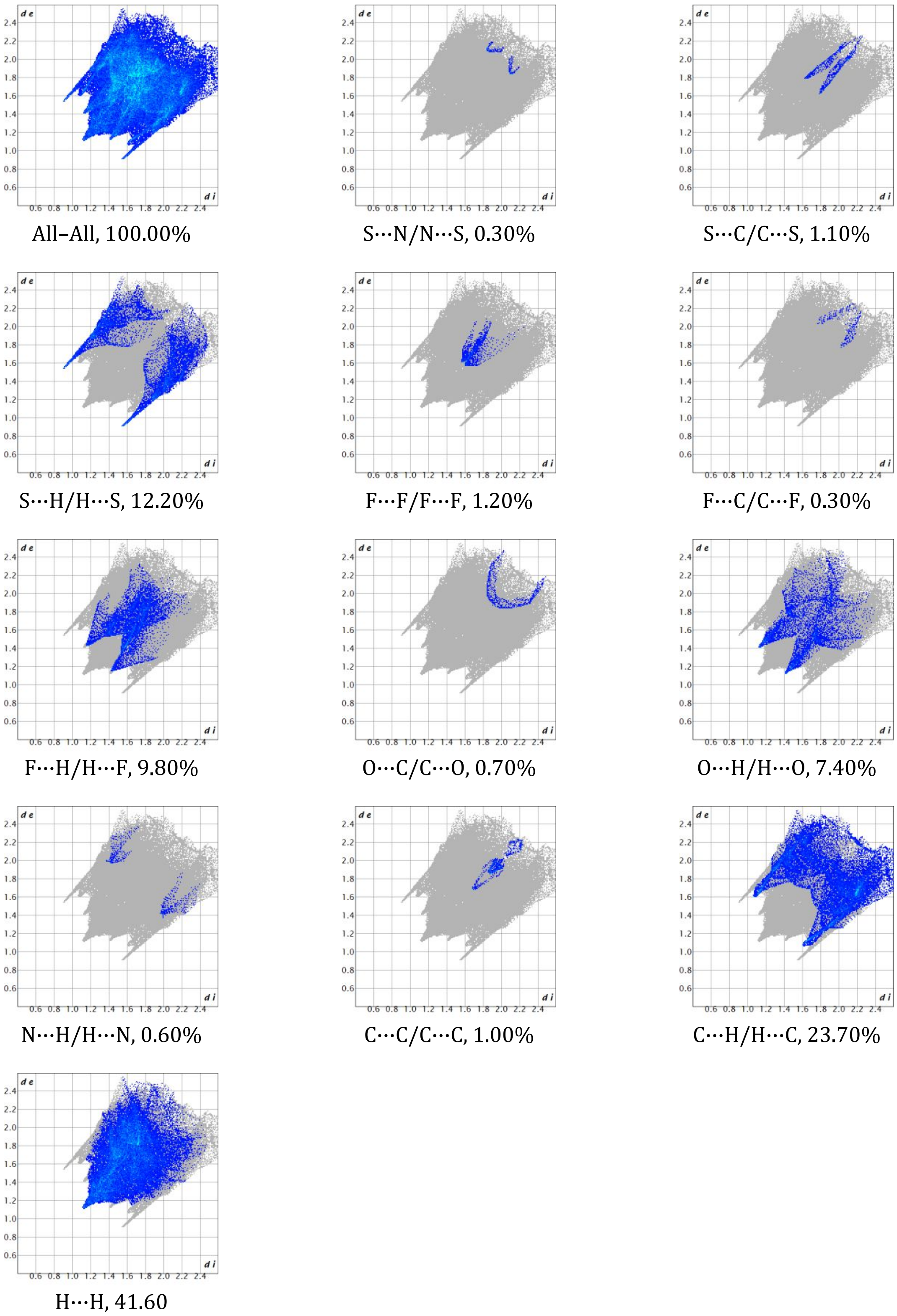

Figure S17. Two–dimensional fingerprint plots and percentage contributions of intermolecular contacts for compound FBTU2.

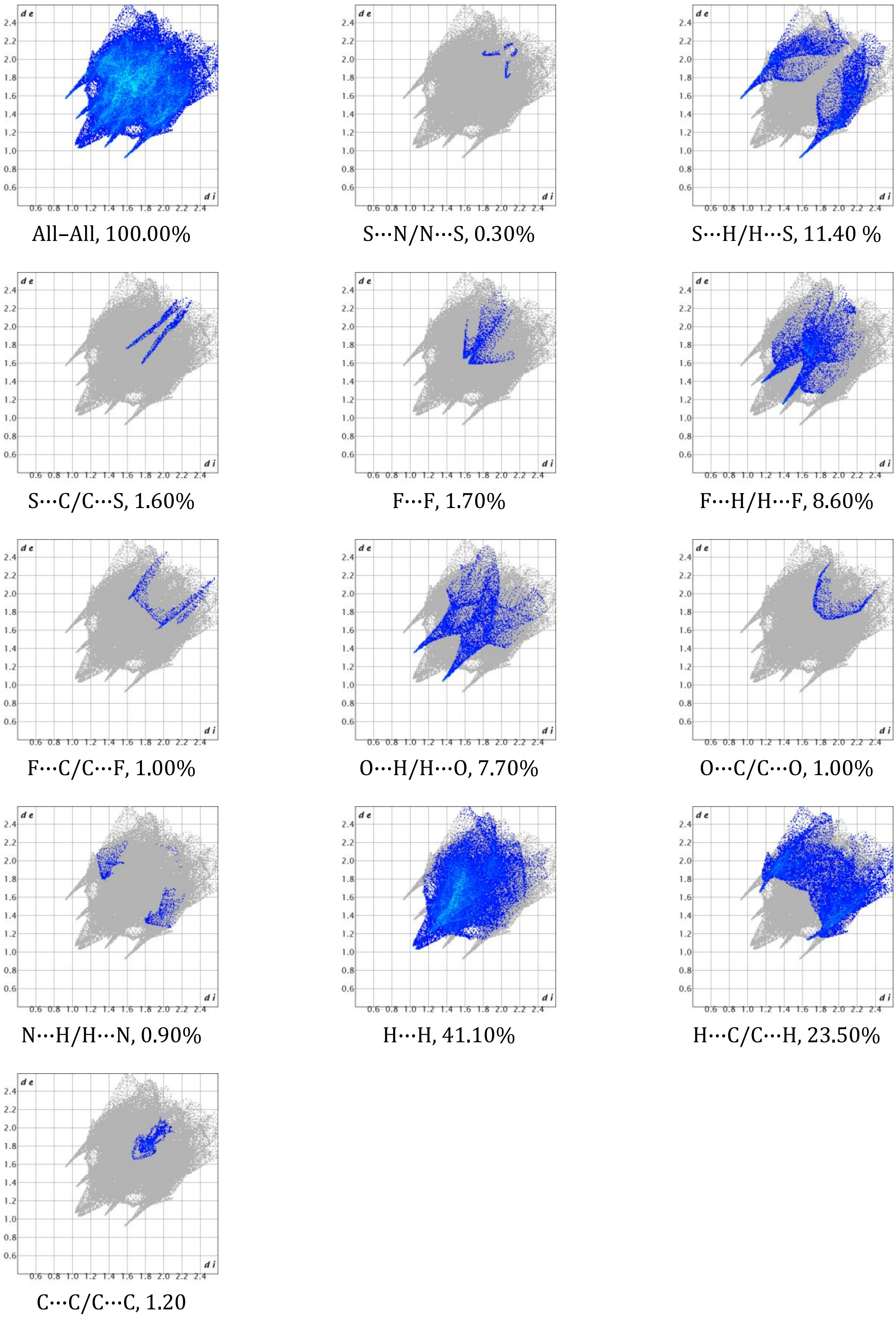

Figure S18. Two–dimensional fingerprint plots and percentage contributions of intermolecular contacts for compound FBTU3.

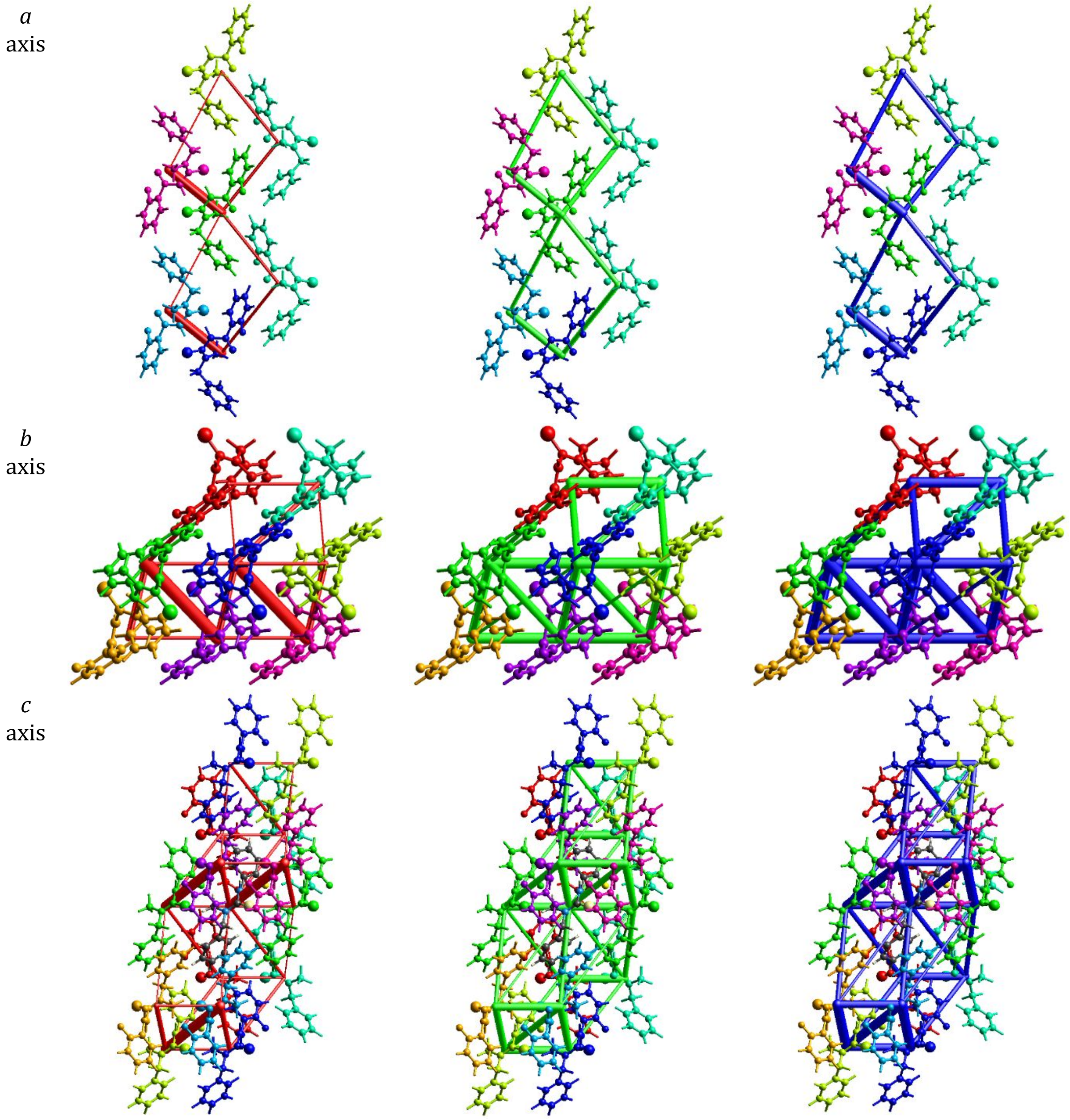

Figure S19. Energy framework diagrams showing Coulombic (red), dispersion (green), and total (blue) interaction energies projected along the *a*–, *b*–, and *c*–axes for compound FBTU1.

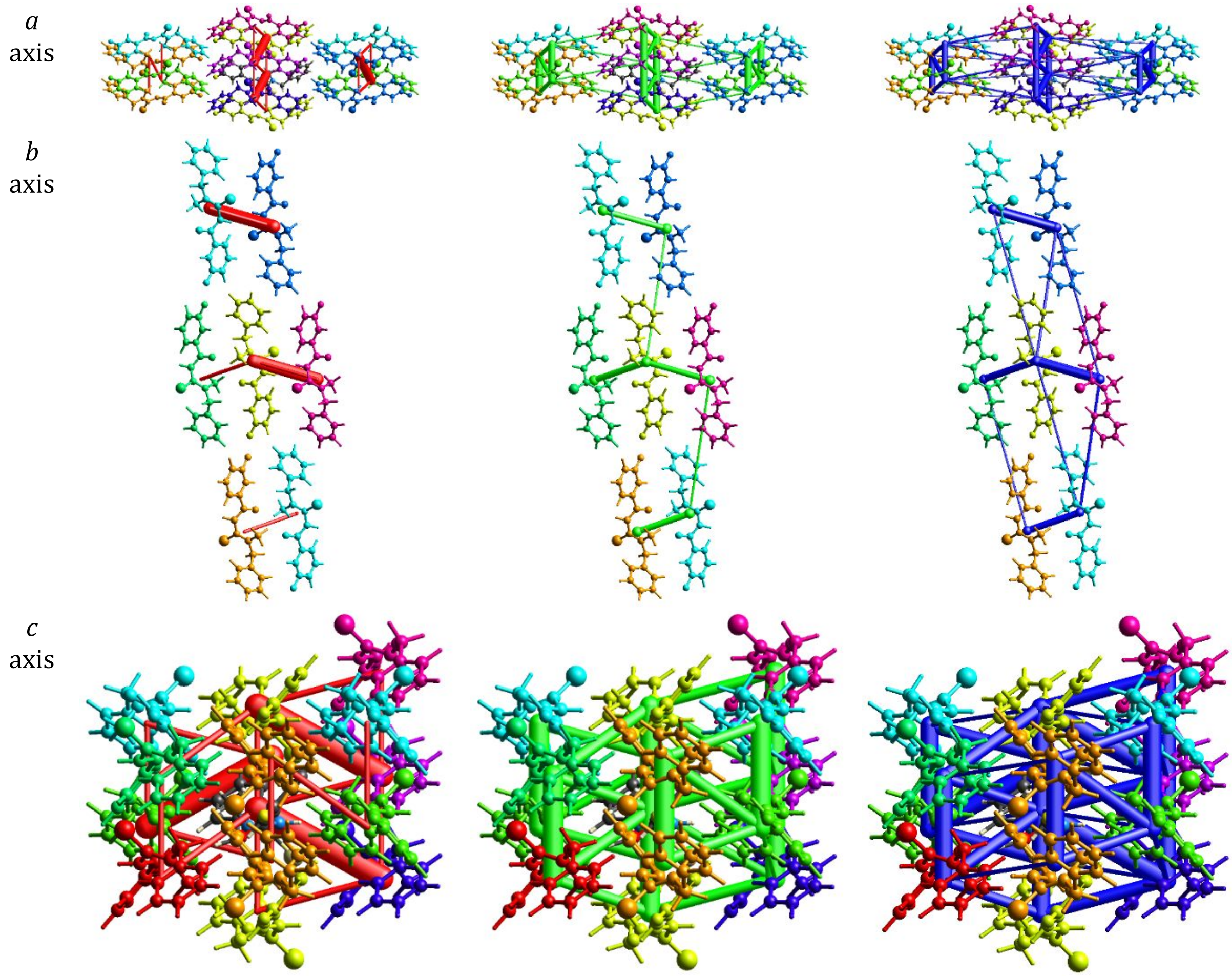

Figure S20. Energy framework diagrams showing Coulombic (red), dispersion (green), and total (blue) interaction energies projected along the *a*–, *b*–, and *c*–axes for compound FBTU2.

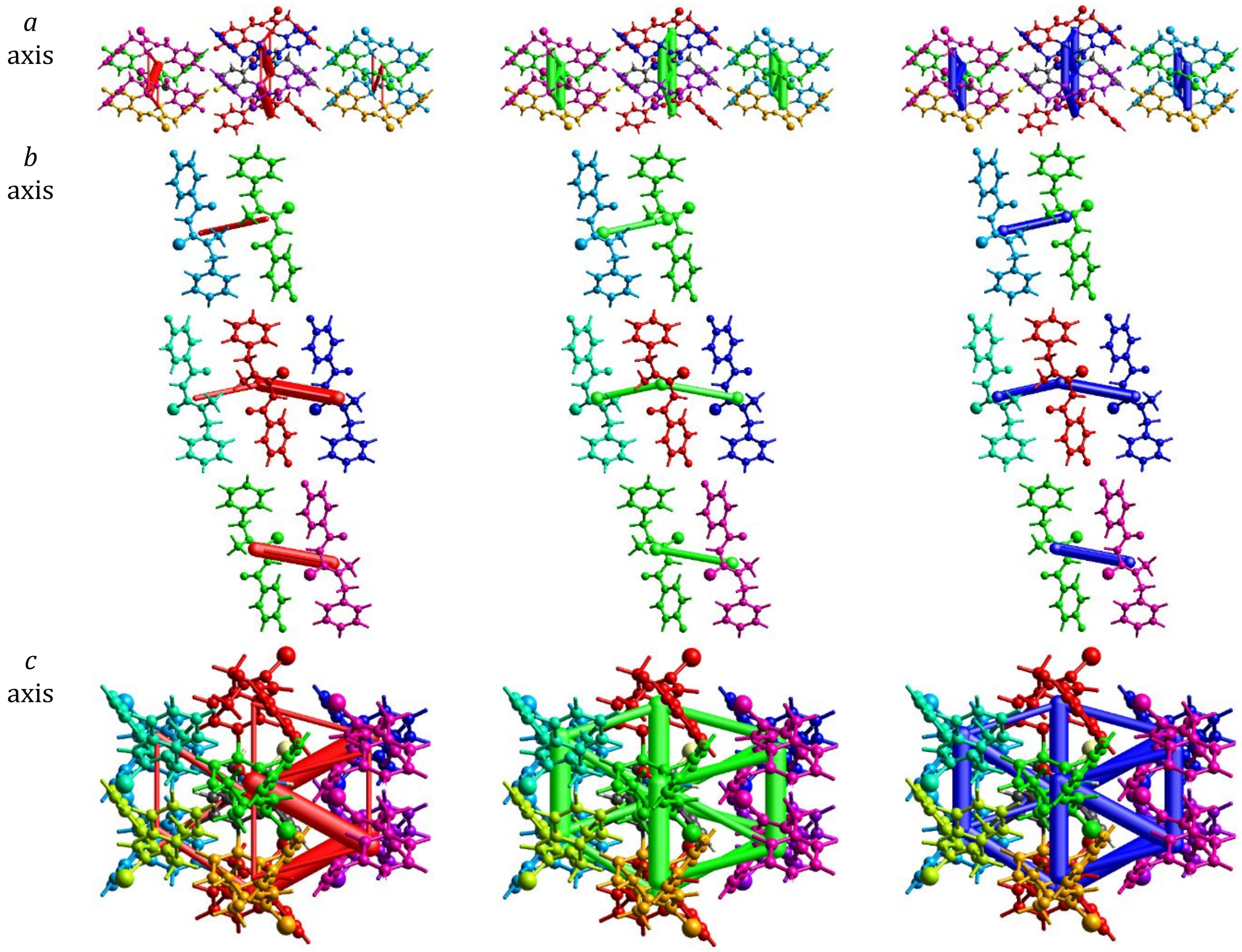

Figure S21. Energy framework diagrams showing Coulombic (red), dispersion (green), and total (blue) interaction energies projected along the *a*–, *b*–, and *c*–axes for compound FBTU3.

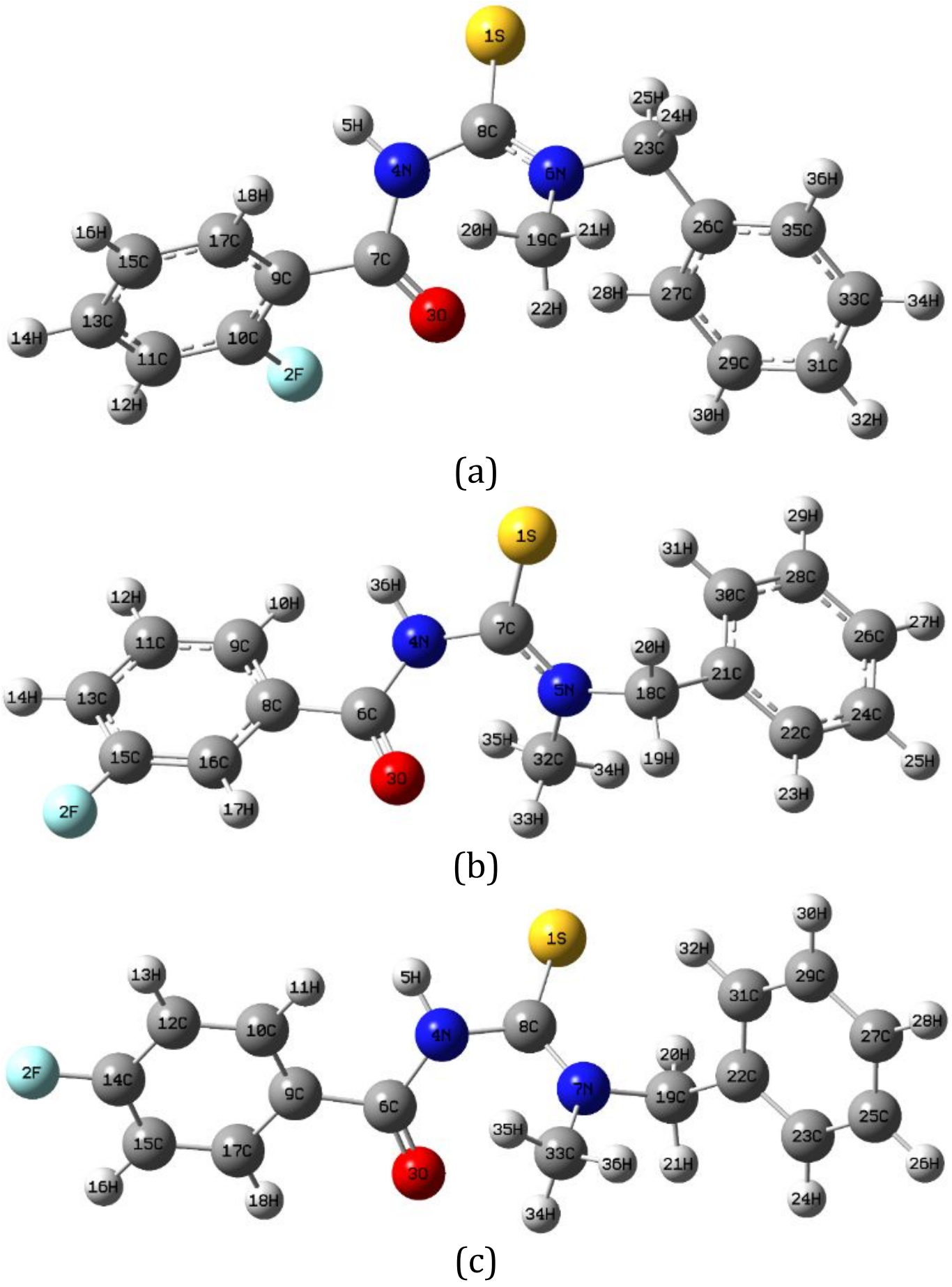

Figure S22. Optimized geometries of compounds FBTU1 (a), FBTU2 (b), and FBTU3 (c) obtained from DFT calculations together with atom numbering.

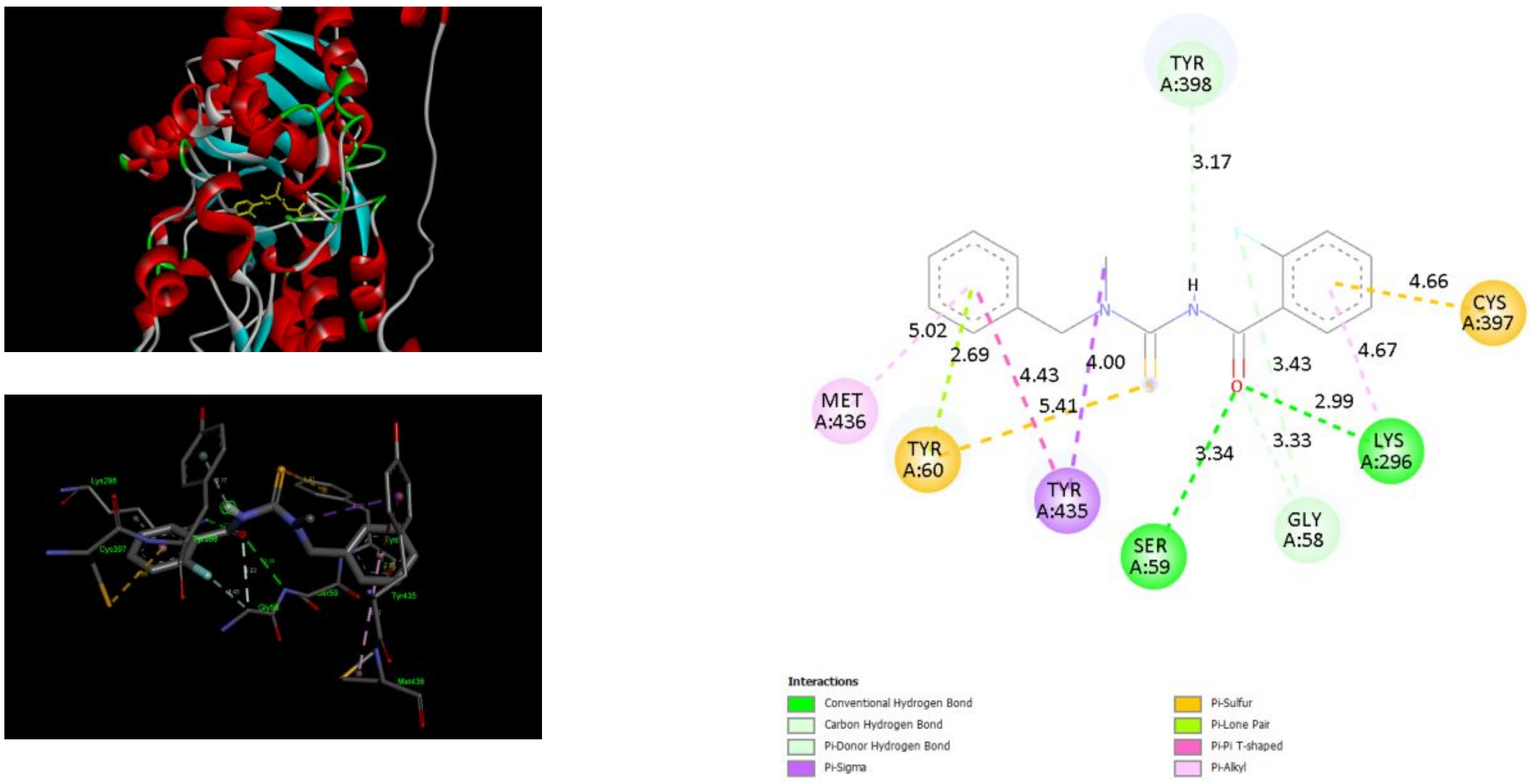

(a)

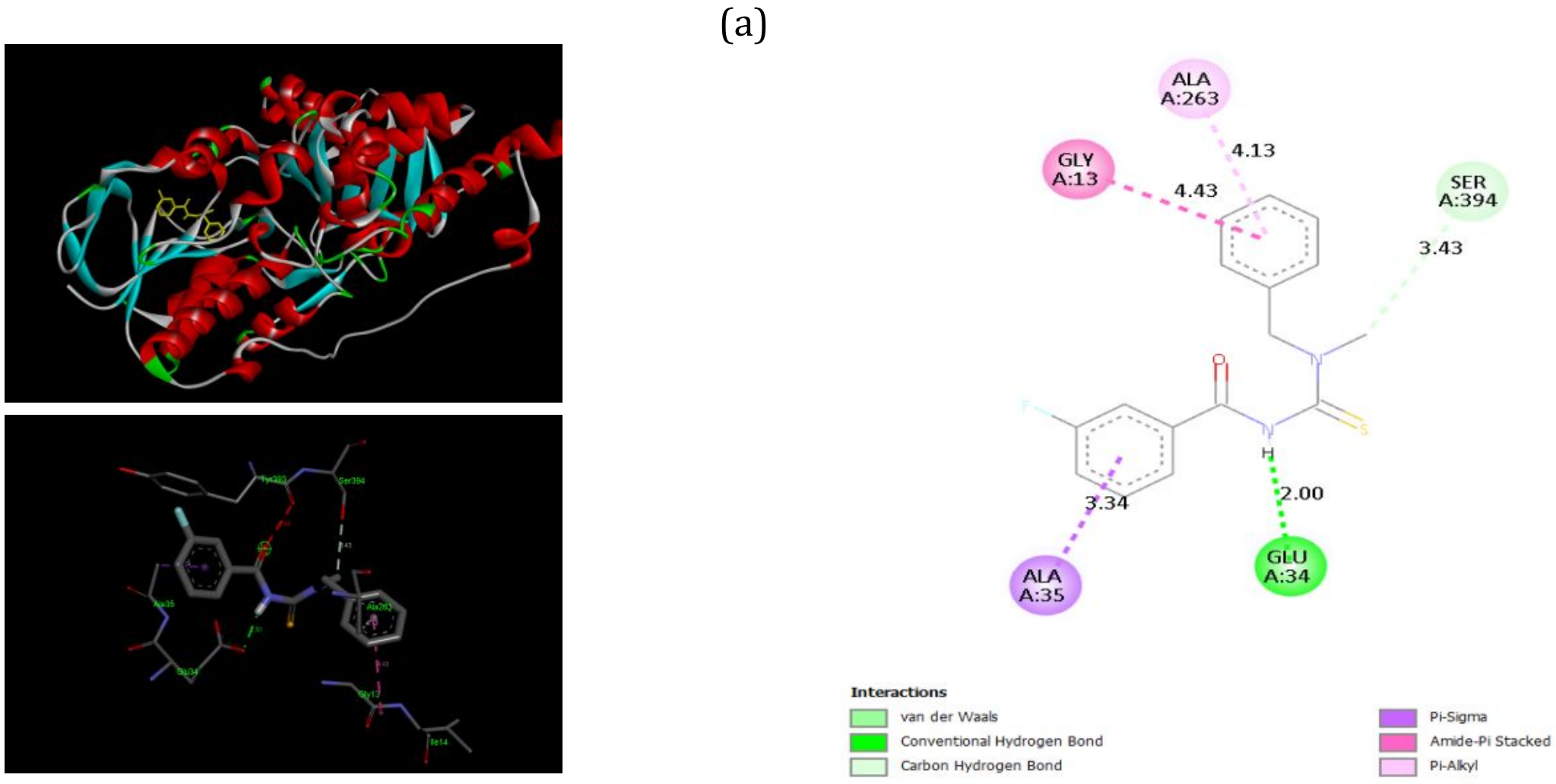

(b)

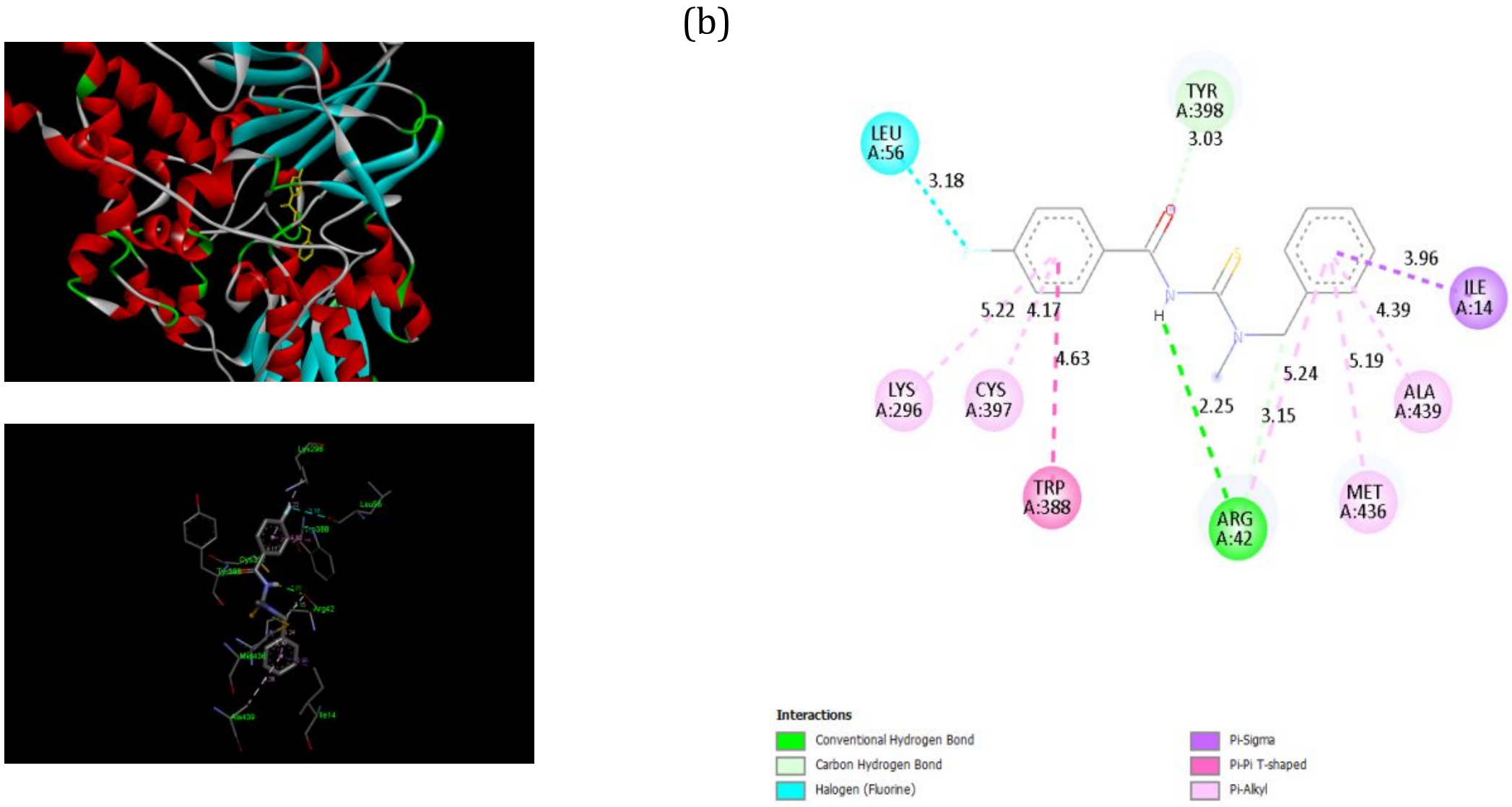

(c)

Figure S23. Three–dimensional binding poses and two–dimensional interaction diagrams of ligands FBTU1 (a), FBTU2 (b), and FBTU3 (c) in the active site of the 1GOS protein.

Table S1. Bond lengths (Å) for FBTU1–FBTU3.

Atom	Atom	Length	Atom	Atom	Length	Atom	Atom	Length	Atom	Atom	Length
FBTU1											
S1	C2	1.6744(19)	C1	C3	1.494(3)	C6	C7	1.376(4)	C12	C13	1.382(3)
F1	C4	1.351(2)	C3	C4	1.374(3)	C7	C8	1.386(3)	C13	C14	1.355(4)
O1	C1	1.212(2)	C3	C8	1.385(3)	C4	C5	1.371(3)	C14	C15	1.356(3)
N1	C1	1.381(2)	N2	C2	1.321(2)	C10	C11	1.503(3)	C15	C16	1.384(3)
N1	C2	1.406(2)	N2	C9	1.467(2)	C11	C12	1.372(3)			
N2	C10	1.475(2)	C5	C6	1.369(3)	C11	C16	1.375(3)			
FBTU2											
S1	C2	1.678(2)	C1	C3	1.486(3)	C5	C6	1.374(4)	C11	C12	1.371(4)
F1	C7	1.357(3)	C3	C4	1.383(3)	C6	C7	1.355(4)	C12	C13	1.362(4)
O1	C1	1.217(3)	C3	C8	1.388(3)	C7	C8	1.366(4)	C13	C14	1.367(4)
N1	C1	1.373(3)	N2	C2	1.327(3)	C9	C10	1.513(3)	C14	C15	1.380(4)
N1	C2	1.398(3)	N2	C9	1.468(3)	C10	C11	1.381(3)			
N2	C16	1.464(3)	C4	C5	1.385(4)	C10	C15	1.368(3)			
FBTU3											
S1	C2	1.6820(17)	C1	C3	1.480(3)	C5	C6	1.373(3)	C11	C12	1.388(3)
F1	C6	1.355(3)	N2	C2	1.329(2)	C6	C7	1.363(4)	C12	C13	1.374(4)
O1	C1	1.222(2)	N2	C9	1.478(2)	C7	C8	1.383(3)	C13	C14	1.366(4)
N1	C1	1.384(2)	N2	C16	1.466(2)	C9	C10	1.511(3)	C14	C15	1.384(3)
N1	C2	1.403(2)	C3	C4	1.391(3)	C10	C11	1.384(3)			
C4	C5	1.382(3)	C3	C8	1.397(3)	C10	C15	1.376(3)			

Table S2. Bond angles (°) for FBTU1–FBTU3.

Atom	Atom	Atom	Angle	Atom	Atom	Atom	Angle	Atom	Atom	Atom	Angle
FBTU1											
C1	N1	C2	124.55(16)	N2	C2	S1	125.24(14)	C5	C6	C7	120.7(2)
C2	N2	C9	123.42(16)	N2	C2	N1	116.19(17)	C6	C7	C8	120.3(2)
C2	N2	C10	121.41(16)	C4	C3	C1	121.80(17)	C3	C8	C7	119.9(2)
C9	N2	C10	114.94(16)	C4	C3	C8	117.66(18)	N2	C10	C11	111.56(16)
O1	C1	N1	123.63(17)	C8	C3	C1	120.54(17)	C12	C11	C10	121.47(19)
O1	C1	C3	123.75(17)	F1	C4	C3	118.80(17)	C12	C11	C16	117.9(2)
N1	C1	C3	112.61(16)	F1	C4	C5	117.75(19)	C16	C11	C10	120.67(18)
N1	C2	S1	118.49(14)	C5	C4	C3	123.4(2)	C11	C12	C13	121.0(2)
C6	C5	C4	118.0(2)	C14	C13	C12	120.4(2)	C13	C14	C15	119.5(2)
C14	C15	C16	120.6(2)	C11	C16	C15	120.6(2)				
FBTU2											
C1	N1	C2	124.5(2)	N2	C2	N1	116.6(2)	C6	C7	F1	118.8(3)
C2	N2	C9	121.67(19)	C4	C3	C1	123.6(2)	C6	C7	C8	122.9(3)
C2	N2	C16	124.2(2)	C4	C3	C8	119.4(2)	C7	C8	C3	118.8(3)
C16	N2	C9	114.16(19)	C8	C3	C1	117.0(2)	N2	C9	C10	112.68(19)
O1	C1	N1	122.5(2)	C3	C4	C5	119.8(3)	C11	C10	C9	120.5(2)
O1	C1	C3	121.9(2)	C6	C5	C4	120.6(3)	C15	C10	C9	121.3(2)
N1	C1	C3	115.6(2)	C7	C6	C5	118.5(3)	C15	C10	C11	118.2(2)
N1	C2	S1	118.90(17)	F1	C7	C8	118.3(3)	C12	C11	C10	120.7(3)
N2	C2	S1	124.47(18)	C13	C12	C11	120.6(3)	C12	C13	C14	119.4(3)
C13	C14	C15	120.2(3)	C10	C15	C14	120.9(2)				
FBTU3											
C1	N1	C2	125.70(15)	C4	C3	C8	119.00(19)	C7	C6	C8	118.4(2)
O1	C1	N1	122.05(18)	C8	C3	C1	118.96(17)	C7	C8	C3	120.6(2)
O1	C1	C3	123.02(17)	C5	C4	C3	120.49(19)	N2	C9	C10	111.11(14)
N1	C1	C3	114.94(16)	C6	C5	C4	118.5(2)	C11	C10	C9	121.44(18)
C2	N2	C9	122.33(15)	F1	C6	C5	118.5(2)	C15	C10	C9	120.16(17)
C2	N2	C16	123.66(16)	F1	C6	C7	118.5(2)	C15	C10	C11	118.4(2)
C16	N2	C9	113.92(15)	C7	C6	C5	123.0(2)	C10	C11	C12	120.3(2)
N1	C2	S1	118.32(13)	C13	C12	C11	120.4(2)	C14	C13	C12	119.6(2)
N2	C2	S1	125.03(14)	C13	C14	C15	120.1(2)	C10	C15	C14	121.2(2)
N2	C2	N1	116.58(15)	C4	C3	C1	122.05(16)				

Table S3. Color–coded visualization of intermolecular interacting molecular pairs together with their corresponding interaction energy components in the crystal structures of compound FBTU1.

Color	N	Symmetry code	R	E_{ele}	E_{pol}	E_{dis}	E_{rep}	E_{tot}
	2	−x, y+1/2, −z+1/2	8.53	−5.286	−1.824	−27.182	11.718	−23.371
	1	−x, −y, −z	12.98	−2.171	−0.557	−12.005	0.000	−13.162
	2	x, y, z	14.08	1.213	−0.274	−6.429	0.000	−4.519
	2	x, y, z	5.78	−10.420	−3.513	−40.887	24.041	−34.370
	2	−x, y+1/2, −z+1/2	9.49	−13.548	−3.214	−17.883	16.051	−22.361
	1	−x, −y, −z	10.52	−6.331	−1.044	−22.037	6.122	−22.874
	2	x, y, z	12.84	2.714	−0.661	−8.792	0.000	−5.276
	1	−x, −y, −z	6.15	−21.209	−8.273	−36.252	24.734	−44.836
	1	−x, −y, −z	6.84	−41.962	−9.528	−32.782	62.819	−41.159
**Total**				−97.000	−28.888	−204.249	145.485	−211.928

Table S4. Color–coded visualization of intermolecular interacting molecular pairs together with their corresponding interaction energy components in the crystal structures of compound FBTU2.

Color	N	Symmetry code	R	E_{ele}	E_{pol}	E_{dis}	E_{rep}	E_{tot}
	1	−x, −y, −z	5.90	−16.127	−5.038	−34.918	29.261	−33.110
	2	−x+1/2, y+1/2, −z+1/2	15.10	−1.901	−0.206	−3.975	0.000	−5.624
	2	x, y, z	4.93	−13.133	−4.237	−57.294	36.412	−44.421
	2	x+1/2, −y+1/2, z+1/2	14.64	−3.372	−0.310	−7.492	0.000	−10.319
	1	−x, −y, −z	5.72	−3.952	−1.313	−32.057	9.950	−26.918
	2	x+1/2, −y+1/2, z+1/2	15.38	0.013	−0.439	−5.607	0.000	−5.193
	2	−x+1/2, y+1/2, −z+1/2	13.62	−2.671	−0.554	−12.002	0.000	−13.686
	1	−x, −y, −z	6.21	−57.490	−10.171	−26.589	71.853	−47.080
	1	−x, −y, −z	6.04	−15.958	−4.677	−31.557	23.359	−33.384
	1	−x, −y, −z	9.12	1.735	−0.318	−2.810	0.045	−0.821
**Total**				−112.856	−27.263	−214.301	170.880	−220.556

Table S5. Color–coded visualization of intermolecular interacting molecular pairs together with their corresponding interaction energy components in the crystal structures of compound FBTU3.

Color	N	Symmetry code	R	E_{ele}	E_{pol}	E_{dis}	E_{rep}	E_{tot}
	2	x, y, z	4.90	−12.990	−5.191	−63.387	37.432	−49.650
	2	x, −y+1/2, z+1/2	14.22	−2.730	−0.257	−7.926	0.000	−9.979
	1	−x, −y, −z	6.12	−0.592	−1.186	−31.766	13.035	−21.114
	2	x, −y+1/2, z+1/2	13.49	1.489	−0.611	−8.445	0.000	−6.231
	1	−x, −y, −z	6.41	−22.171	−6.681	−30.165	25.575	−38.855
	2	−x, y+1/2, −z+1/2	14.44	−1.062	−0.334	−9.549	0.000	−9.685
	1	−x, −y, −z	6.10	−56.628	−10.124	−27.370	67.248	−49.659
	1	−x, −y, −z	5.79	−15.353	−4.444	−36.185	34.060	−29.992
	2	−x, y+1/2, −z+1/2	14.83	1.688	−0.074	−3.710	0.000	−1.501
**Total**				−108.349	−28.902	−218.503	177.350	−216.666

Table S6. Mulliken atomic charge distributions of compounds FBTU1–FBTU3 obtained from DFT calculation.

FBTU1		FBTU2		FBTU3	
**Atom**	**Charge**	**Atom**	**Charge**	**Atom**	**Charge**
S1	−0.2332	S1	−0.2454	S1	−0.2482
F2	−0.2001	F2	−0.2251	F2	−0.2210
O3	−0.3229	O3	−0.3387	O3	−0.3415
N4	−0.3994	N4	−0.4076	N4	−0.4090
H5	0.2727	N5	−0.3190	H5	0.2672
N6	−0.3422	C6	0.4253	C6	0.4214
C7	0.4062	C7	0.1430	N7	−0.3191
C8	0.1349	C8	−0.1845	C8	0.1439
C9	−0.2148	C9	−0.0530	C9	−0.1934
C10	0.2859	H10	0.1024	C10	−0.0444
C11	−0.1177	C11	−0.0928	H11	0.1066
H12	0.1184	H12	0.1101	C12	−0.1258
C13	−0.0717	C13	−0.1058	H13	0.1182
H14	0.1114	H14	0.1192	C14	0.2556
C15	−0.0917	C15	0.2501	C15	−0.1230
H16	0.1056	C16	−0.0644	H16	0.1202
C17	−0.0486	H17	0.1293	C17	−0.0174
H18	0.1121	C18	−0.0684	H18	0.1230
C19	−0.1685	H19	0.1291	C19	−0.0680
H20	0.1271	H20	0.1928	H20	0.1929
H21	0.1253	C21	−0.1293	H21	0.1283
H22	0.1626	C22	−0.0671	C22	−0.1292
C23	−0.0040	H23	0.0871	C23	−0.0672
H24	0.1276	C24	−0.0913	H24	0.0869
H25	0.1903	H25	0.0957	C25	−0.0915
C26	−0.1692	C26	−0.0866	H26	0.0955
C27	−0.0411	H27	0.0968	C27	−0.0866
H28	0.1248	C28	−0.0938	H28	0.0966
C29	−0.1023	H29	0.0974	C29	−0.0942
H30	0.0989	C30	−0.0438	H30	0.0973
C31	−0.0853	H31	0.1231	C31	−0.0429
H32	0.0946	C32	−0.1725	H32	0.1231
C33	−0.0943	H33	0.1531	C33	−0.1720
H34	0.0935	H34	0.1366	H34	0.1527
C35	−0.0695	H35	0.1296	H35	0.1294
H36	0.0844	H36	0.2682	H36	0.1360

Table S7. Molecular docking interactions of ligands FBTU1–FBTU3 within the active site of the 1GOS enzyme.

No	Protein residue (Chain:Res:Atom)	Ligand moiety	Distance (Å)	Interaction type	Subtype	Protein role	Ligand role
FBTU1							
1	A:SER59:N	O1	3.338	Hydrogen bond	Conventional hydrogen bond	H–Donor	H–Acceptor
2	A:LYS296:NZ	O1	2.991	Hydrogen bond	Conventional hydrogen bond	H–Donor	H–Acceptor
3	A:GLY58:CA	O1	3.329	Hydrogen bond	Carbon hydrogen bond	H–Donor	H–Acceptor
4	A:GLY58:CA	F1	3.434	Hydrogen bond; Halogen	Carbon hydrogen bond; Halogen (Fluorine)	H–Donor; Halogen Acceptor	H–Acceptor; Halogen
5	A:TYR398	H1	3.175	Hydrogen bond	Pi–Donor hydrogen bond	Pi–Orbitals	H–Donor
6	A:TYR435	C9	3.995	Hydrophobic	Pi–Sigma	Pi–Orbitals	C–H
7	A:CYS397:SG	Aromatic ring (2–F)	4.663	Non–covalent interaction	Pi–Sulfur	Sulfur	Pi–Orbitals
8	A:TYR60	S1	5.412	Non–covalent interaction	Pi–Sulfur	Pi–Orbitals	Sulfur
9	A:TYR60:O	Aromatic ring (–CH_2–)	2.685	Non–covalent interaction	Pi–Lone Pair	Lone Pair	Pi–Orbitals
10	A:TYR435	Aromatic ring (–CH_2–)	4.432	Hydrophobic	Pi–Pi T–shaped	Pi–Orbitals	Pi–Orbitals
11	A:LYS296	Aromatic ring (2–F)	4.668	Hydrophobic	Pi–Alkyl	Alkyl	Pi–Orbitals
12	A:MET436	Aromatic ring (–CH_2–)	5.016	Hydrophobic	Pi–Alkyl	Alkyl	Pi–Orbitals
FBTU2							
1	A:GLU34:OE1	H1	2.000	Hydrogen bond	Conventional hydrogen bond	H–Acceptor	H–Donor
2	A:SER394:OG	C16	3.431	Hydrogen bond	Carbon hydrogen bond	H–Acceptor	H–Donor
3	A:ALA35:CB	Aromatic ring (3–F)	3.342	Hydrophobic	Pi–Sigma	C–H	Pi–Orbitals
4	A:GLY13:C,O;ILE14:N	Aromatic ring (–CH_2–)	4.429	Hydrophobic	Amide–Pi stacked	Amide	Pi–Orbitals
5	A:ALA263	Aromatic ring (–CH_2–)	4.129	Hydrophobic	Pi–Alkyl	Alkyl	Pi–Orbitals
FBTU3							
1	A:ARG42:O	H1	2.247	Hydrogen bond	Conventional hydrogen bond	H–Acceptor	H–Donor
2	A:TYR398:CA	O1	3.031	Hydrogen bond	Carbon hydrogen bond	H–Donor	H–Acceptor
3	A:ARG42:O	C9	3.150	Hydrogen bond	Carbon hydrogen bond	H–Acceptor	H–Donor
4	A:LEU56:O	F1	3.183	Halogen	Halogen (Fluorine)	Halogen Acceptor	Halogen
5	A:ILE14:CD1	Aromatic ring (–CH_2–)	3.959	Hydrophobic	Pi–Sigma	C–H	Pi–Orbitals
6	A:TRP388	Aromatic ring (4–F)	4.626	Hydrophobic	Pi–Pi T–shaped	Pi–Orbitals	Pi–Orbitals
7	A:LYS296	Aromatic ring (4–F)	5.215	Hydrophobic	Pi–Alkyl	Alkyl	Pi–Orbitals
8	A:CYS397	Aromatic ring (4–F)	4.172	Hydrophobic	Pi–Alkyl	Alkyl	Pi–Orbitals
9	A:ARG42	Aromatic ring (–CH_2–)	5.244	Hydrophobic	Pi–Alkyl	Alkyl	Pi–Orbitals
10	A:MET436	Aromatic ring (–CH_2–)	5.193	Hydrophobic	Pi–Alkyl	Alkyl	Pi–Orbitals
11	A:ALA439	Aromatic ring (–CH_2–)	4.394	Hydrophobic	Pi–Alkyl	Alkyl	Pi–Orbitals
